# Causal effects between personality and psychiatric traits and lung cancer: a bidirectional two-sample Mendelian randomization and bibliometric study

**DOI:** 10.3389/fpsyt.2024.1338481

**Published:** 2024-09-12

**Authors:** Siyuan Chen, Zhijuan Du, Yuhui Qin, Yanan Li, Yu Pan, Yu Qiao, Juan Chen, Zhengyang Hou, Shuai Jin, Haitao Tao, Heying Yu, Jiapei Qin, Mingzhen Zhu, Zhijie Wang, Zhefeng Liu

**Affiliations:** ^1^ State Key Laboratory of Molecular Oncology, CAMS Key Laboratory of Translational Research on Lung Cancer, Department of Medical Oncology, National Cancer Center/National Clinical Research Center for Cancer/Cancer Hospital, Chinese Academy of Medical Sciences and Peking Union Medical College, Beijing, China; ^2^ Department of Medical Oncology, Senior Department of Oncology, The Fifth Medical Center of PLA General Hospital, Beijing, China; ^3^ Medical School of Chinese PLA, Beijing, China; ^4^ Department of Medical Psychology, The First Medical Center of PLA General Hospital, Beijing, China; ^5^ Department of Medical Oncology, The First Affiliated Hospital of Bengbu Medical College, Bengbu, China; ^6^ Department of Anesthesia Operating Room, Shandong Provincial Hospital Affiliated to Shandong First Medical University, Jinan, Shandong, China; ^7^ Department of Psychology, School of Social Development and Public Policy, Fudan University, Shanghai, China; ^8^ Department of Medical Oncology, The Third Medical Center of PLA General Hospital, Beijing, China; ^9^ Department of Thoracic Surgery, Affiliated Hospital of Jining Medical University, Jining, China

**Keywords:** psychiatric, personality, lung cancer, Mendelian randomization, bibliometric

## Abstract

**Introduction:**

The causality between personality and psychiatric traits and lung cancer (LC) remains unclear. Therefore, we aimed to elucidate the causality between these traits and LC.

**Methods:**

Bidirectional two-sample Mendelian randomization (MR) and bibliometric approaches were conducted to estimate the causality between personality (neuroticism, extraversion, agreeableness, conscientiousness, and openness) and psychiatric (schizophrenia, attention-deficit/hyperactivity disorder [ADHD], major depressive disorder, autism spectrum disorder, bipolar disorder, insomnia, and anxiety) traits and LC and its subtypes (lung squamous cell carcinoma, lung adenocarcinoma, and small cell LC). Summary data of these traits were extracted from large datasets (17,375–462,341 participants). Inverse variance weighting was used as the primary MR analysis, with supplementary models, including MR-Egger and weighted medians. Sensitivity analyses were conducted to detect pleiotropy. Bibliometric data were retrieved from the Web of Science Core Collection, Scopus, and PubMed. The main mapping techniques adopted were co-word, collaboration, and citation analyses.

**Results:**

Schizophrenia was associated with an increased risk of LC (odds ratio [OR] = 1.077, 95% confidence interval [CI] = 1.030–1.126, *P* = 0.001). Moreover, LC increased the risk of ADHD (OR = 1.221, 95% CI = 1.096–1.362, *P* < 0.001). No significant bidirectional associations were observed between other mental traits and LC and its subtypes. Causality, psychiatry, and psychiatric comorbidity are emerging keywords. Research dynamics and landscapes were revealed.

**Conclusion:**

This study suggests that schizophrenia is a risk factor for LC and that LC is a risk factor for ADHD. Furthermore, causality, psychiatry, and psychiatric comorbidity have become emerging research trends in related fields.

## 1 Introduction

Lung cancer (LC) is the second most common cancer and the primary cause of cancer-related deaths worldwide, with approximately 2.2 million new cases and 1.8 million deaths reported annually (1). LC subtypes mainly include lung squamous cell carcinoma (LUSC), lung adenocarcinoma (LUAD), and small cell lung cancer (SCLC), each of which has distinct risk factors and biological characteristics (2).

Personality and psychiatric traits are involved in carcinogenesis (2). LC risk is associated with neuroticism and extraversion (3). However, other studies did not confirm these findings (4). Other personality dimensions within the Big Five trait taxonomy (5), including agreeableness, conscientiousness, and openness to experience, are associated with health outcomes (6). Patients with schizophrenia may have a higher LC risk than the general population (7). A previous meta-analysis supported the protective effect of schizophrenia on LC (8). However, another meta-analysis concluded that the association lacked certainty (9). Studies on the incidence of LC in patients with other psychiatric traits, including attention-deficit/hyperactivity disorder (ADHD), major depressive disorder (MDD), autism spectrum disorder (ASD), bipolar disorder (BD), and insomnia, are also inconclusive (10–12). These traits can influence LC development by affecting health behaviors, including smoking (13). The associations of these traits with smoking have been demonstrated, and the causal inference may be confounded by a lack of adjustment for these known risk factors (14).

Personalities can change during major life events, such as developing LC (15). A higher prevalence of psychological and psychiatric illnesses has been reported among patients with LC, which may be associated with the stigma surrounding smoking (16). However, the causal relationship between LC and personality and psychiatric traits remains poorly understood (16).

Mendelian randomization (MR) is an epidemiological methodology that uses genetic variants, including single-nucleotide polymorphisms (SNPs), as instrumental variables (IVs) to estimate the causality between exposure and outcome (17). MR analysis constitutes a natural randomized controlled trial that assesses causality between exposure and outcome at the genetic level while excluding reverse causality (17). Two-sample MR facilitates the utilization of summary statistics from genome-wide association studies (GWASs) without directly analyzing individual-level data (18). Bibliometrics uses information on words, authors, or citations shared between articles to describe the structure of the scientific literature and demonstrate the influence of a study through data on the number and nature of citations it receives (19). Researchers can utilize changes in publication networks to document and visualize the development of a scientific field (19).

Notably, previous MR studies have investigated the causal effects of neuroticism and insomnia on LC and found that both are positively associated with an increased LC risk (12, 20, 21). However, previous findings reported in rigorously designed prospective cohort studies did not draw that conclusion (4, 22–24). Thus, we conducted this study which aimed to elucidate whether there is a forward or reverse causal association between personality and psychological traits and LC and its subtypes and to further elucidate the nature of this potential association. We also present an overview of the research dynamics and landscape using science mapping.

## 2 Materials and methods

### 2.1 Study design

**Figure 1:**
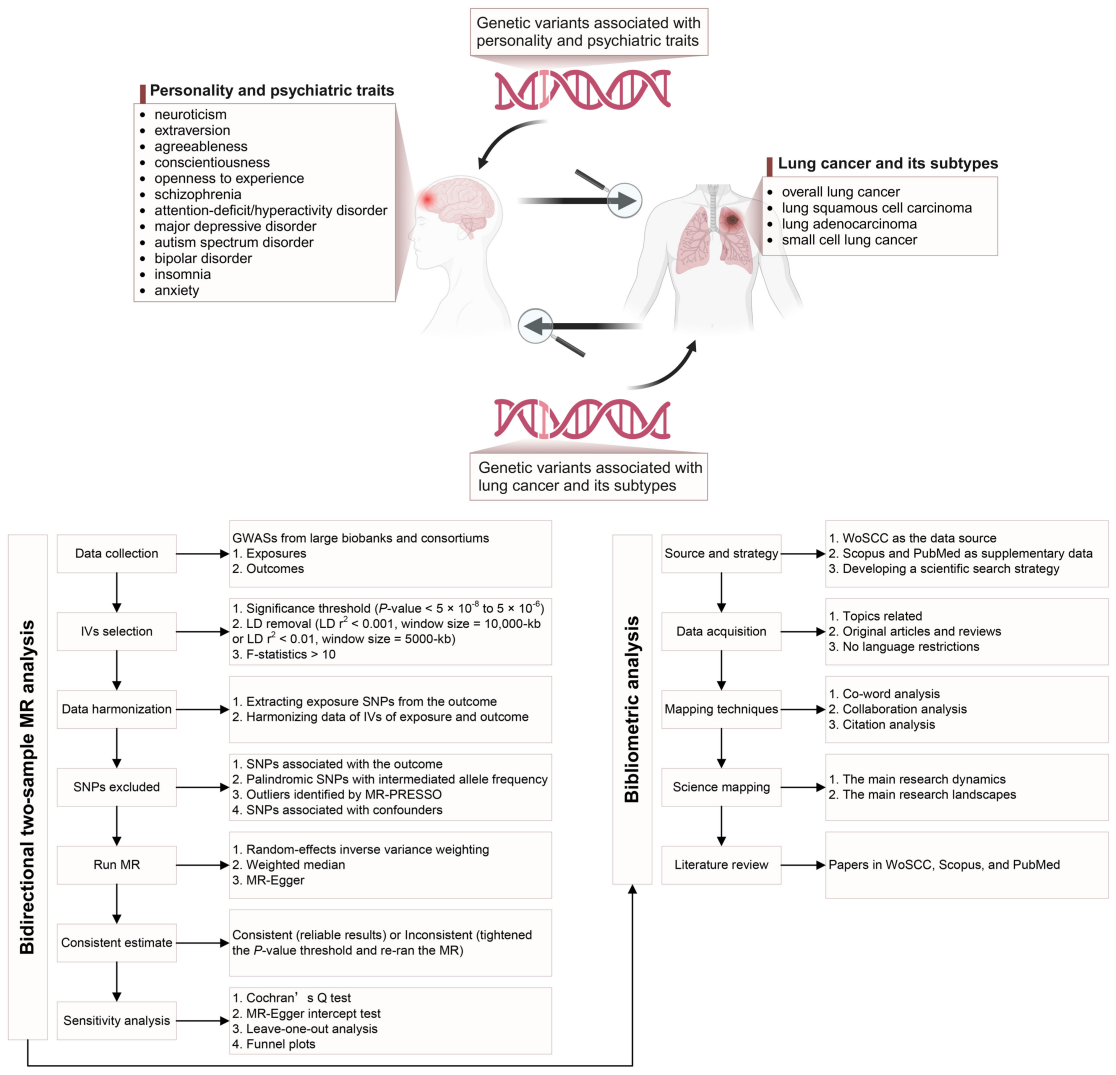
Overview of the study design [created with BioRender.com].

The overall study design is presented in **Figure 1**. We conducted a two-sample MR study to assess the causal relationships between genetically predicted personality (neuroticism, extraversion, agreeableness, conscientiousness, and openness) and psychiatric (schizophrenia, ADHD, MDD, ASD, BD, and insomnia) traits and LC (including its subtypes: LUSC, LUAD, and SCLC) in forward and reverse directions. Genetic variants, specifically SNPs, were used as IVs. The causal inference of MR relies on the following three core assumptions: relevance, independence, and exclusion restriction (**Supplementary Figure S1**) (25). Horizontal pleiotropy exists when the last two assumptions are unmet (26). We performed a bibliometric analysis that enabled us to better understand the scientific literature on the association between these traits and LC, thereby providing complementary viewpoints for MR studies through science mapping and a scientific literature review. The following three techniques were used for bibliometric mapping to quantify the networks and connections among relevant publications: (i) co-word analysis, which identifies the most commonly used or co-occurring terms to reveal critical concepts in the research field; (ii) collaboration analysis, which examines the co-occurrence of countries, institutions, and authors in a group of articles; and (iii) citation analysis, which evaluates the frequency and interconnections of article citations (27).

### 2.2 Bidirectional two-sample MR analysis

#### 2.2.1 Data sources

SNPs for neuroticism were obtained from the UK Biobank and the MRC Integrative Epidemiology Unit (MRC-IEU), which included 374,323 individuals of European ancestry (28). Summary statistics for extraversion, agreeableness, conscientiousness, and openness were selected from the MRC-IEU and Genetics of Personality Consortium, which included 17,375 individuals of European descent (28). Schizophrenia GWAS data from the Psychiatric Genomics Consortium (PGC) were used to extract the IVs (n = 320,404; 76,755 cases and 243,649 controls) (29). Genetic instruments for ADHD (n = 55,374; 20,183 cases and 35,191 controls), MDD (n = 173,005; 59,851 cases and 113,154 controls), ASD (n = 46,351; 18,382 cases and 27,969 controls), and BD (n = 51,710; 20,352 cases and 31,358 controls) were extracted from the PGC (30). The IVs for insomnia and anxiety were acquired from the MRC-IEU database, comprising data from 462,341 and 484,598 individuals of European ancestry, respectively (28, 30, 31). Summary-level GWAS data for instrumental SNPs associated with LC and its subtypes were extracted from Transdisciplinary Research in Cancer of the Lung and the International Lung Cancer Consortium. The participants were patients with LC with European ancestry (n = 85,716; 29,266 cases and 56,450 controls) (32). The GWAS analysis was adjusted for principal components and subsequently stratified based on histologic subtypes (**Supplementary Table S1**). The summarized statistics analyzed in this study were approved by the ethics committees of the original studies. The diagnosis of LC requires pathological or cytological confirmation. Detailed information, such as recruitment process and genetic data quality control, can be found in the original studies. The use of the summarized statistics analyzed in this study was approved by the ethics review committees of the original studies.

#### 2.2.2 Selection of genetic instruments

Independent genetic variants that were strongly associated with exposure were acquired using the following criteria: Typically, we selected instrumental SNPs using a *P*-value < 5 × 10^-8^ and linkage disequilibrium (LD) r^2^ < 0.001 within a 10,000-kb window. However, considering that some personality and psychiatric traits, such as extraversion, agreeableness, and conscientiousness, have been relatively less studied in GWASs, the number of SNPs meeting genome-wide significance is limited. Therefore, we utilized SNPs with a more relaxed threshold, and this set the minimum extraction criteria to *P* < 5 × 10^-6^ and LD r^2^ < 0.01 within a 5,000-kb window. We calculated the F-statistics for each genetic instrument to assess the strength of the genetic variants, considering an F-statistic > 10 indicative of a strong IV (33).

We extracted exposure SNPs from the summary statistics of outcomes and subsequently harmonized the associated SNP data of exposure and outcomes (28). SNPs associated with the outcome, as well as palindromic SNPs with intermediate allele frequencies, were discarded. Additionally, SNPs that exhibited a potential association with outliers identified using the MR pleiotropy residual sum and outlier test were excluded from the MR analysis. The selected SNPs were further examined using PhenoScanner—a platform that provides comprehensive genotype–phenotype correlation information (34). We investigated whether these SNPs were linked to LC risk factors, including tobacco smoking, environmental tobacco smoke exposure, obesity, and alcohol consumption, and excluded SNPs that exhibited genome-wide associations with these confounding factors (14).

#### 2.2.3 MR analyses

Forward MR analyses were performed to explore the causality of personality and psychiatric traits on LC and its subtypes. Reverse MR analyses were conducted to assess the effects of LC and its subtypes on these traits. We used random-effects inverse variance weighting (IVW), MR-Egger, and the weighted median as MR models. Among these models, IVW was selected as the primary method for assessing exposure–outcome effects. The IVW analysis can provide the most reliable estimates of causality and sensitivity to pleiotropy (35). Other approaches have been utilized as supplementary analyses, providing reliable estimates in various scenarios despite wider confidence intervals (CIs) and lower effectiveness (5). The weighted median approach enables a robust causal assessment, assuming that up to 50% of IVs are invalid (36). MR-Egger allows for pleiotropy across all IVs, although it requires that pleiotropy be independent of the variant–exposure association (37). Relevant scatter plots were plotted. In cases where only two significant SNPs were available for certain phenotypes, such as agreeableness, IVW was the only applicable model.

A Bonferroni-corrected *P*-value < 0.05/48 (48 is the product of the number of exposures and the number of outcome events) = 0.001 was considered statistically significant for multiple comparisons. A *P*-value threshold of 0.001 was deemed reliable. If the estimates were inconsistent across different MR models, the *P*-value threshold would need to be tightened, followed by re-running the MR analysis (18).

#### 2.2.4 Sensitivity analysis

Sensitivity analyses were conducted to detect possible heterogeneity and pleiotropy. Cochran’s Q test was used to estimate heterogeneity, with *P* < 0.05 indicating significance (38). However, as IVW contributed to the primary result, heterogeneity was considered acceptable (39). When heterogeneity is present, the IVW random effect model is employed (38, 39). Horizontal pleiotropy was assessed using the MR-Egger intercept test, with *P*-values < 0.05 suggesting a high level of pleiotropic bias that could influence causal estimation (18). Additionally, leave-one-out analysis was performed to determine whether a lone SNP strongly drives a specific causal inference. Moreover, funnel plots were used to evaluate probable directional pleiotropy.

Statistical analyses for the MR analysis were performed using the TwoSampleMR (version 0.5.6) package in R (version 4.2.3). We refer to the STROBE-MR guidelines for MR analysis (40).

### 2.3 Bibliometric analysis

#### 2.3.1 Data source, search strategy, and data acquisition

Data were obtained from the Science Citation Index Expanded and Social Sciences Citation Index of Web of Science Core Collection (WoSCC). The analysis of Scopus and PubMed was a supplementary view. To ensure the reliability of the results, we focused exclusively on site-specific cancers, as combining analyses across multiple cancer sites may lead to diluted effects (23). Therefore, we only included studies that focused on LC. We referred to the Medical Subject Headings and relevant review articles to identify appropriate search terms for accurate searches (41). Medical oncologists and psychologists (YH and YP) were consulted to provide supplementary information. The search terms used were “lung” and “cancer,” “tumor,” “neoplasm,” or “carcinoma,” combined with a list of relevant personality and psychiatric traits (**Supplementary Methods**).

We conducted a systematic literature search in the WoSCC from January 1900 to May 2023 and completed data acquisition on May 12, 2023, to avoid potential errors due to database updates. Guidelines, statements, and editorials were excluded. Only original articles and reviews were retained; no language restrictions were imposed. The validity of the bibliometric analysis using original articles and reviews of WoSCC was demonstrated (42). The data acquisition processes for Scopus and PubMed were similar to those for WoSCC.

#### 2.3.2 Science mapping and literature review

Bibliometric analysis results are always manifested through science mapping (27). We utilized the R package bibliometrix (version 4.1.2), VOSviewer (version 1.6.19), and CiteSpace (version 6.2.5) for data conversion, analysis, and visualization, respectively. The bibliometric mapping techniques adopted included co-word, collaboration, and citation analyses.

We extracted essential bibliographic data from the selected articles, including countries, institutions, authors, keywords, publication years, and total citations (TCs). Subsequently, science maps were generated to illustrate the research dynamics and landscapes on the associations between LC and personality and psychiatric traits. Keyword co-occurrence was used because it effectively reflects the study content and evolutionary trends (43) and thus provides a snapshot of existing studies. Furthermore, the articles’ growth trends and networks were established to visualize the research dynamics. The research landscape was presented through the country, institution, and author collaboration networks. We used a co-citation analysis to assess the significance of the cited articles. This analysis indicated the significance of scholars’ attachment to a cited article, where a greater frequency of citations indicated a higher relevance in developing a focal field (44).

We manually reviewed articles from WoSCC, Scopus, and PubMed by reading their titles, abstracts, and full texts to gain an in-depth understanding of relevant research. Any disagreements were discussed with expert authors until a consensus was reached. Based on bibliometric mapping and a literature review, we interpreted the MR results.

## 3 Results

### 3.1 Causal effects of personality and psychiatric traits on LC

#### 3.1.1 Selection of genetic instruments

To genetically predict neuroticism, we used a total of 116 SNPs. Additionally, we used four, two, five, and eight SNPs for extraversion, agreeableness, conscientiousness, and openness, respectively (**Supplementary Tables S2**–**S6**). Overall, 217 SNPs were used to predict schizophrenia, including 12, 18, 10, 16, 42, and 13 for ADHD, MDD, ASD, BD, insomnia, and anxiety, respectively (**Supplementary Tables S7**–**S13**). The F-statistics of the instrumental SNPs was > 10, indicating that the IVs had sufficient predictive strength for these traits.

After filtering out the SNPs with outliers, ambiguous palindromic sequences, and those potentially associated with outcomes and confounding factors, the remaining SNPs were used for forward MR analyses (**Supplementary Tables S14**–**S25**).

#### 3.1.2 MR estimates

**Figure 2:**
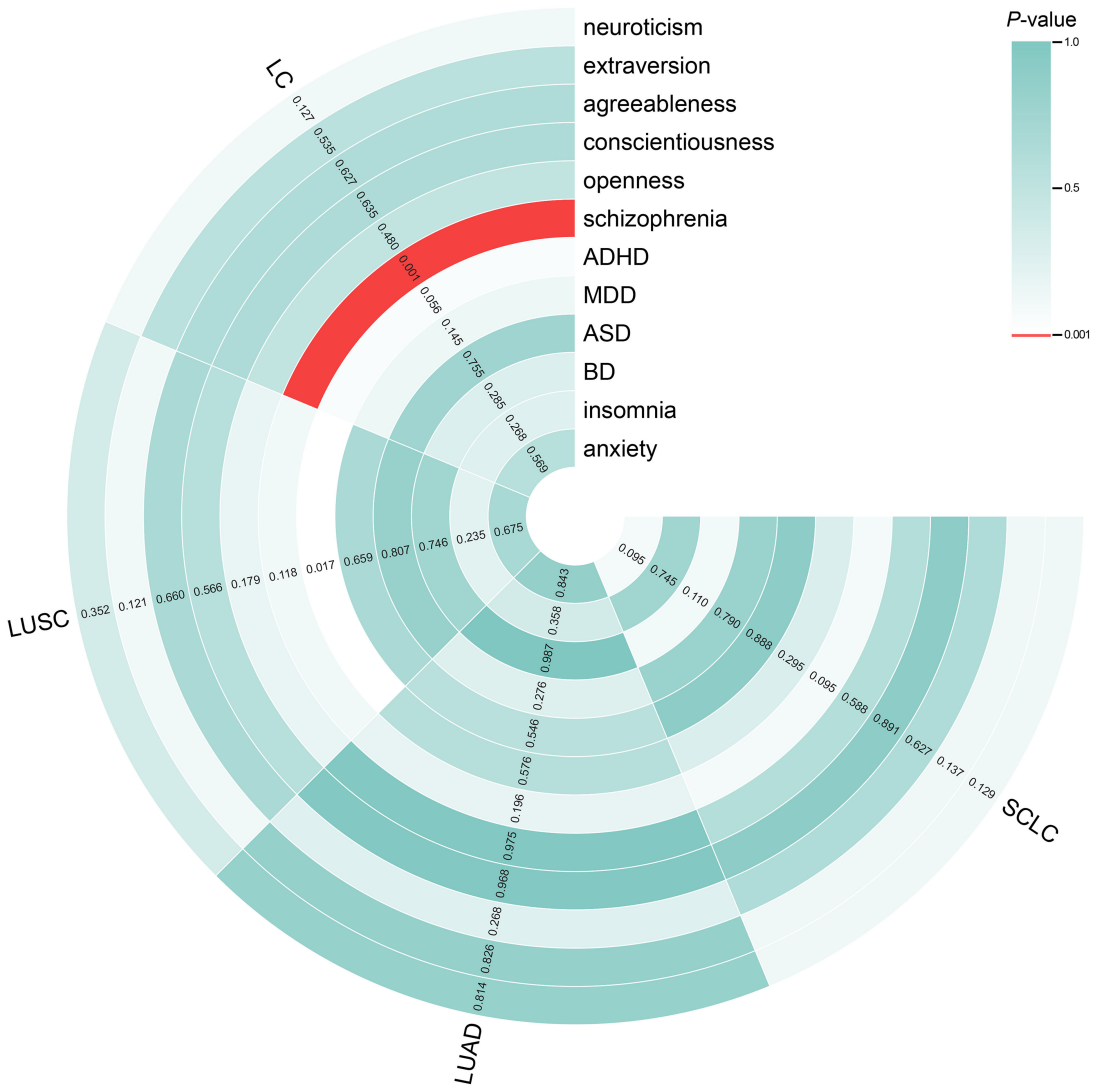
Circle heatmap for causal associations between genetically predicted personality and psychiatric traits and LC and its subtypes. The color of each block represents the inverse variance weighting (IVW)-derived *P*-values obtained from every IVW analysis. Blocks shown in red denote a statistically significant *P*-value of < 0.001, whereas green or white blocks denote non-significant *P*-values of > 0.001. IVW, inverse variance weighting; LC, lung cancer.

The results of the IVW analyses of the forward MR are shown in **Figure 2**. The analysis revealed a significant association between genetically proxied schizophrenia and increased risk of overall LC (odds ratio [OR] = 1.077, 95% CI = 1.030–1.126, *P* = 0.001). The consistent direction of estimations across other MR approaches further strengthened the confidence in the causal inference (**Supplementary Table S26**). However, no significant causal effects were found for other traits (neuroticism, extraversion, agreeableness, conscientiousness, openness, ADHD, MDD, ASD, BD, or insomnia) on the risk of LC or LC subtypes (**Figure 2**). The scatter plots (**Supplementary Figure S2**) displayed the potential impact of personality and psychiatric traits on LC and its subtypes, as observed using different MR models.

#### 3.1.3 Sensitivity analysis

Cochran’s Q test revealed heterogeneity in the causal inference between schizophrenia and LC risk (*P* < 0.05); however, this heterogeneity was acceptable because we used the IVW random effect model to obtain the main result. Although heterogeneity was detected in other results, it did not invalidate the MR estimates of the IVW analysis. Furthermore, the MR-Egger intercept test did not identify any pleiotropy (*P* > 0.05), indicating the absence of pleiotropic bias in certain contexts of heterogeneity. The related leave-one-out analyses and funnel plots are presented in **Supplementary Figures S3**, **S4**, respectively. The estimates revealed that the significant causal effect detected was not biased by specific SNPs.

### 3.2 Causal effects of LC on personality and psychiatric traits

#### 3.2.1 Selection of genetic instruments

Fifteen SNPs were used to genetically predict overall LC, whereas 7, 13, and 2 SNPs were used to predict LUSC, LUAD, and SCLC, respectively (**Supplementary Tables S27**–**S30**). Similar to forward MR analyses, the F-statistics of the SNPs exceeded the critical value of 10. After excluding SNPs with outliers, palindromic sequences, and those potentially associated with outcomes and confounding factors, the remaining SNPs were used for reverse MR analyses (**Supplementary Tables S31**–**S34**).

#### 3.2.2 MR estimates

**Figure 3:**
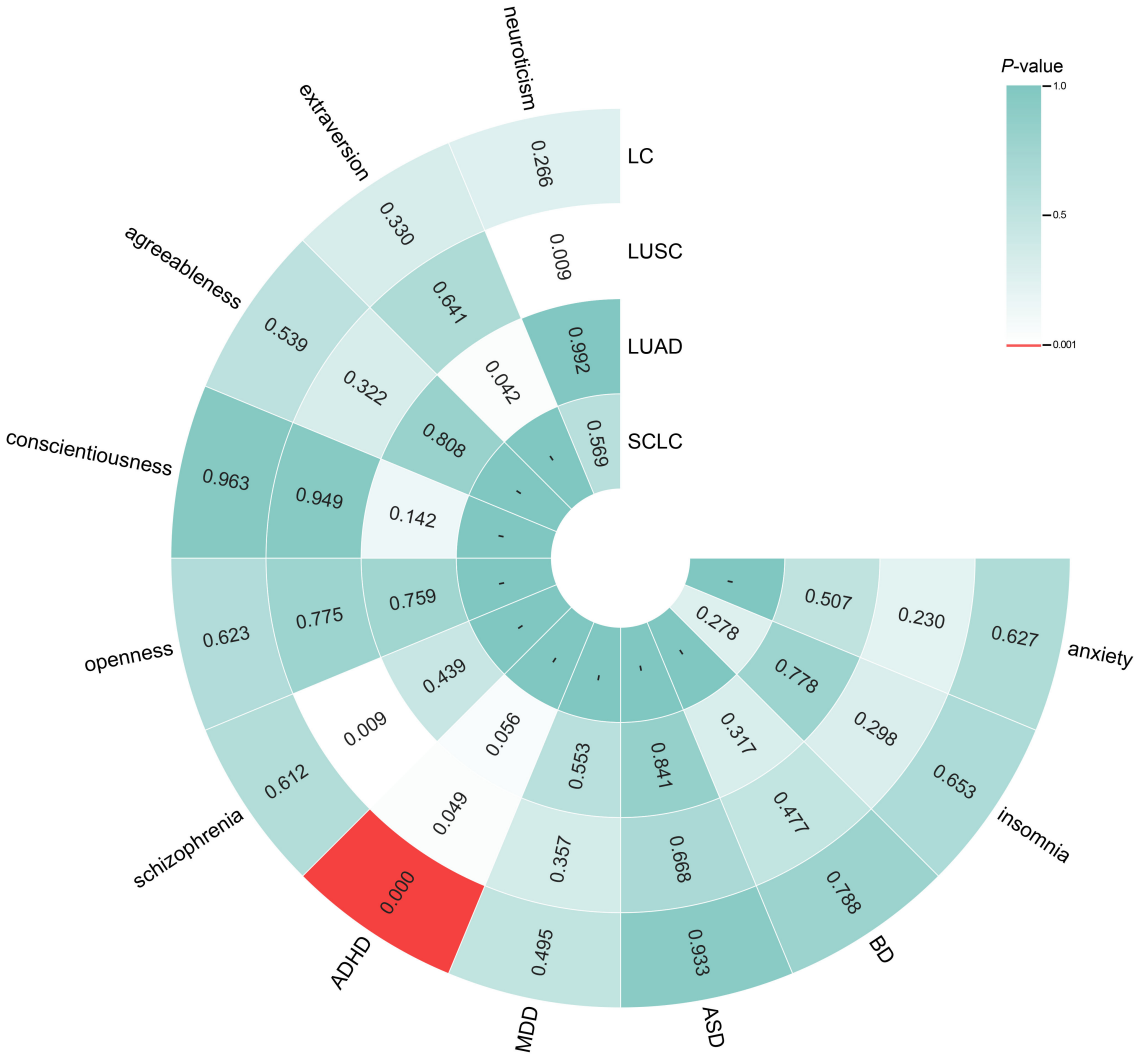
Circle heatmap for causal associations between genetically predicted lung cancer, its subtypes and personality and psychiatric traits. The color of each block represents the inverse variance weighting (IVW)-derived *P*-values obtained from every IVW analysis. Blocks shown in red denote a statistically significant *P*-value of < 0.001, whereas green or white blocks denote non-significant *P*-values > 0.001; a hyphen (-) indicates that the value is unavailable. IVW, inverse variance weighting; LC, lung cancer.

The findings of the IVW analyses on reverse MR are shown in **Figure 3**. The analysis revealed a significant causal relationship between genetically predicted overall LC and increased ADHD risk (OR = 1.221, 95% CI = 1.096–1.362, *P* < 0.001), which is consistent with other MR approaches (**Supplementary Table S35**). However, this model revealed no significant causal impact of overall LC, LUSC, LUAD, or SCLC on the risk of other traits (neuroticism, extraversion, agreeableness, conscientiousness, openness, schizophrenia, MDD, ASD, BD, insomnia or anxiety; **Figure 3**). The scatter plots (**Supplementary Figure S5**) revealed the potential influence of LC and its subtypes on personality and psychiatric traits, detected through different MR models.

#### 3.2.3 Sensitivity analysis

Cochran’s Q test detected no significant heterogeneity in the causal inference between overall LC and ADHD risk (*P* > 0.05). The MR-Egger intercept was < 0.05 only when examining the impact of overall LC on ASD; this suggests the potential presence of pleiotropy. The remaining MR-Egger intercept calculations yielded *P*-values > 0.05, indicating no significant horizontal pleiotropy. Relevant leave-one-out analyses and funnel plots are shown in **Supplementary Figures S6**, **S7**, respectively. The significant causal effect did not rely on a lone SNP.

### 3.3 Bibliometric analysis

#### 3.3.1 Output and time trend of publications

**Figure 4:**
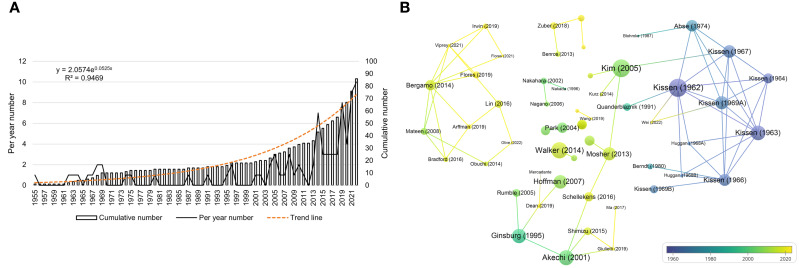
Visualization of publications. **(A)** is the annual distribution of publications. **(B)** is the overlay visualization map of the top-cited publications.

Overall, 84 articles and 2 reviews regarding the association between personality and psychiatric traits and LC were identified in the WOSCC database. As shown in **Figure 4A**, the number of articles increased over the past 60 years. The annual publication count ranged from 1 to 10, with the highest number of publications in 2022 (n=10). The total publication count of relevant articles exhibited exponential growth, as depicted by the formula in **Figure 4A**. A certain degree of citation relationship was found among the publications (**Figure 4B**), with the highest cited articles being those by Kissen (1962), Kim (2005), and Walker (2014), with a TC count of 120, 115, and 98, respectively.

#### 3.3.2 Collaboration analysis

**Figure 5:**
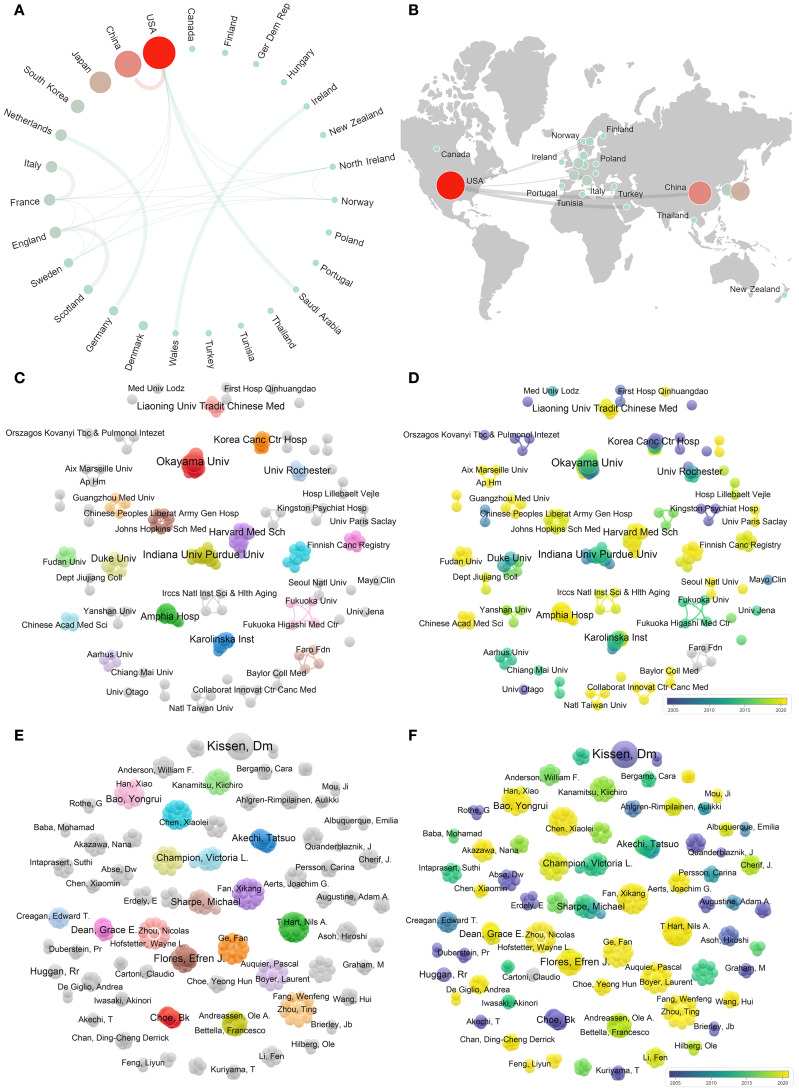
Visualization network of cooperation. **(A)** represents the cooperative relationships between countries; the size of the nodes indicates the number of publications, and the weight of the lines represents the closeness of collaboration. **(B)** represents a world map showing the cooperative relationships between continents. **(C)** represents the cooperative relationships between institutions, and the colors represent the clusters automatically calculated using VOSviewer. **(D)** shows the temporal trends in cooperative relationships among institutions; the colors represent the annual periods of publications, in which the color gradient from purple to yellow shows the annual occurrence time from 2005 to 2020. **(E)** represents the cooperative relationships between authors, with colors indicating the clusters automatically calculated using VOSviewer. **(F)** represents the temporal trends in cooperative relationships between authors, with colors representing the periods of annual publications, in which the color gradient from purple to yellow shows the annual occurrence time from 2005 to 2020.

The articles were sourced from 27 countries, 197 institutions, and 495 authors. The United States (n = 22), China (n = 15), and Japan (n = 10) contributed the most articles (**Supplementary Table S36**). **Figures 5A, B** show that there was a higher level of collaboration and communication between European and American countries, whereas African countries had limited involvement. The collaboration between the United States and China was found to be particularly close. As shown in **Figure 5C**, there were over 10 collaboration clusters among global institutions. Institutions, such as the University of Otago, Kingston Psychiatric Hospital, and Mayo Clinic, were early contributors to research on the association between personality and psychiatric traits and LC (**Figure 5D**). China Academy of Chinese Medical Sciences, Amphia Hospital, and Harvard Medical School have recently started focusing on related research. Four institutions contributed more than three articles, with Okayama University publishing the highest number of articles (n=4) and being cited 37 times (**Supplementary Table S37**). Multiple collaboration networks have been established among the authors (**Figure 5E**). Kissen published most articles (n=7), which had the highest number of citations (n = 433) (**Supplementary Table S38**). As shown in **Figure 5F**, the purple section indicates that this author has been involved in the field earlier, whereas Ge, Fan, Hart T, Nils A, and others have also conducted related research recently.

#### 3.3.3 Citation analysis

**Figure 6:**
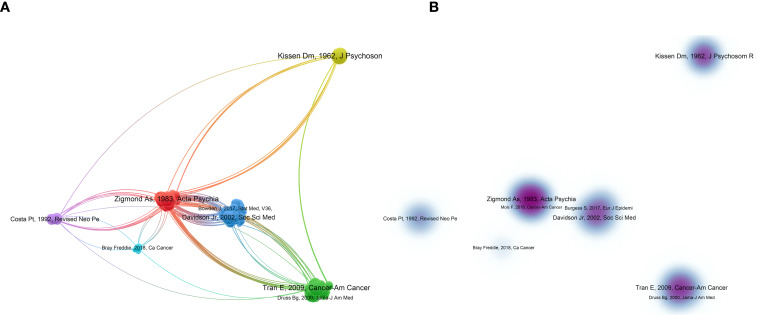
Visualization network of co-cited references.

Highly cited articles can provide information about the current understanding of a topic. VOSviewer was used to calculate 2467 references, as shown in **Figure 6**. The colors in **Figure 6A** represent the automatically calculated clusters, whereas those in B represent the density view of A. The top 10 most cited articles (**Supplementary Table S39**) were all articles, with three of them published in high-impact journals. The study “Personality in Male Lung Cancer Patients” had the highest number of co-citations.

#### 3.3.4 Keywords analysis

**Figure 7:**
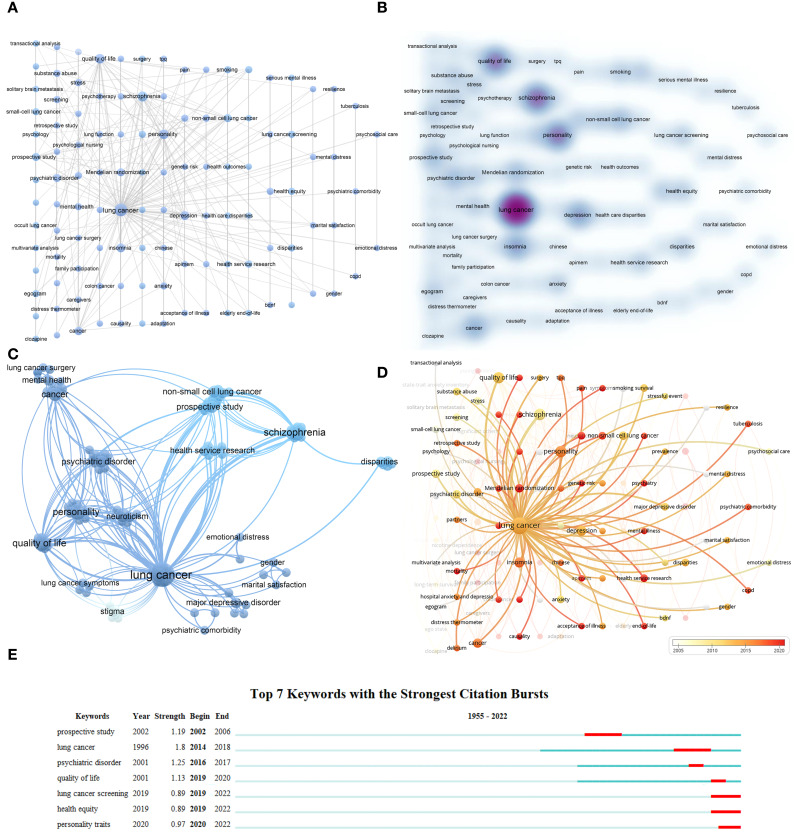
Visualization of keywords in research linking personality and psychiatric traits with lung cancer. **(A)** represents the visualization network with more than one occurrence. **(B)** represents the density plot corresponding to **(A)**. **(C)** is the clustered network view. **(D)** shows the temporal trend of the keyword “lung cancer” and its co-occurrence with other terms, with colors representing annual periods, from white to red indicating co-occurrence years from 2005 to 2020. **(E)** represents the top seven keywords with the strongest citation bursts in related research.

Keyword analysis can reveal research directions and hotspots. One hundred fifteen keywords were analyzed, as shown in **Figures 7A, B**. The top three keywords regarding co-occurrence frequency were “lung cancer” (n = 30), “schizophrenia” (n = 9), and “personality” (n = 8) (**Supplementary Table S40**). **Figure 7C** displays keyword clustering. By analyzing the connections between the “lung cancer” node and other nodes, we examined the temporal trends in research on the association between personality and psychiatric traits and LC, as depicted in **Figure 7D**. Recently, research has been focused on “Mendelian randomization,” “causality,” “psychiatry,” and “psychiatric comorbidity,” as indicated by the red nodes. Citespaces was used to identify burst keywords, which are considered indicators of research trends. The top seven keywords with the strongest citation bursts (**Figure 7E**) represented emerging hotspots in the field. The keywords “lung cancer” and “psychiatric disorders” had a simultaneous burst between 2016 and 2017. Recently, keywords such as “lung cancer screening,” “health equity,” and “personality traits” have been prominent.

Supplementary bibliometric analysis using Scopus and PubMed databases can be found in **Supplementary Figure S8**.

## 4 Discussion

To the best of our knowledge, no comprehensive large-scale MR study exploring the causal effects of personality and psychiatric traits on LC has been conducted, and no bibliometric analyses in related fields have been conducted. Therefore, we systematically assessed the causality of genetically predicted personality and psychiatric traits on LC and its subtypes, as well as the causality of genetically proxied LC and its subtypes on these traits. The results demonstrated a causal effect of genetically predicted schizophrenia on overall LC risk, along with a causal effect of genetically predicted overall LC on ADHD. Moreover, our results revealed research outputs, cooperation networks, emerging trends, and important areas using various bibliometric maps, such as keyword co-occurrence, providing a true “eagle’s-eye view” of the dynamics and landscapes of the relevant scientific literature. Studies have explored the association between schizophrenia and LC and yielded varied findings (7, 45). Notably, lower LC rates in patients with schizophrenia were reported as early as the 1980s (46). Several hypotheses have been proposed to explain these findings. First, the medication for schizophrenia may reduce lung carcinogenesis (47). Second, patients with schizophrenia die early of unnatural causes before the onset of smoking-mediated illness (48). Third, social isolation and hospitalization may protect patients with schizophrenia from stress-mediated cancers (49). Fourth, smoking is restricted in psychiatric wards (50). Protective and behavioral confounding factors in patients with schizophrenia may mask the associated LC risk. Indeed, schizophrenia may affect LC by altering neuroendocrine and immune functions (5). Cohort studies have demonstrated that patients with schizophrenia have a higher incidence rate of LC (7). No randomized controlled clinical trials have provided conclusive evidence thus far. However, considering the perspective of biopsychosocial models, schizophrenia may influence physiological health through specific mechanisms, potentially leading to the development of lung cancer (51). Our findings provide, for the first time, evidence from the MR analysis, which supports the notion that schizophrenia is associated with LC risk. Furthermore, for the first time, we hypothesized that LC was a risk factor for ADHD. Patients with LC are more prone to experiencing psychiatric disorders, and the cause may be related to cancer or cancer treatment (52). A retrospective cohort study also reported an association between ADHD and increased risk of colorectal cancer, suggesting a potential association between cancer and ADHD (53). However, owing to potential limitations arising from poor survival among patients with LC and ethical difficulties, large-scale prospective studies exploring the causal impact of LC on personality and psychiatric traits are lacking (54). The MR approach, as used in the present study, is unaffected by environmental factors and confounders, and the application of Bonferroni correction helped prevent false-positive results, both enhancing the credibility of our findings. Based on our positive results, we propose that LC screening may be necessary for patients with schizophrenia. Additionally, healthcare providers should remain vigilant for the potential occurrence of LC in patients with ADHD. Randomized controlled trials are necessary to further verify the causal relationship between LC and the risk of developing ADHD, as well as to investigate the causal relationship of schizophrenia on the risk of developing LC.

Notably, previous MR studies have revealed that neuroticism and insomnia are associated with an increased LC risk (20, 21). In this study, no genetically predicted causal relationship was observed between neuroticism and insomnia and LC. The potential factors underlying the differences between our results and those of previous studies include, first, the use of PhenoScanner to exclude SNPs associated with confounding factors. Potential outliers and confounders may influence the previously reported associations among LC, neuroticism, and insomnia. Additionally, we employed the Bonferroni correction method to reduce the potential for false positives (34). A prospective cohort study in the UK Biobank failed to identify an association between insomnia and higher risk of LC, which supports the findings of our study (55). Our results are consistent with those of previous studies reported in rigorously designed prospective cohorts (4). Previous studies have only focused on the causal relationship between a single mental trait and LC (20, 21). In contrast, our study included 12 psychiatric and personality traits, making it the most comprehensive study to date covering psychiatric and personality traits. These mental traits have reliable GWAS data. Additionally, we investigated the bidirectional relationship between these traits and LC. All these aspects increase the credibility and value of our research.

In the subgroup analysis, we identified no trait that could causally induce LC subtypes. Similarly, no LC subtype had a specific causal impact on these traits. The results of this study provide meaningful insights into the biopsychosocial models (51). Our results indicate that there is specificity in the causal relationship between psychiatric traits and LC.

The frequency of keywords indicates that schizophrenia and personality are the most researched traits in the relevant field. Other traits, such as ADHD, need more attention. The keyword co-occurrence network suggests that, recently, research trends have focused on causality, psychiatry, and psychiatric comorbidity, and MR has gained stature as an emerging methodology. Exploring the genetic mechanisms associated with risk factors, early prevention, and screening remain important topics. The collaborative networks of countries, institutions, and authors reveal that the United States, Okayama University, and Kissen, respectively, are highly influential contributors to this field. Both TC and co-citation analyses indicate that Kissen’s 1962 article is prominent (3).

The strength of this study is its application of the innovative combination of MR and bibliometric methods. The MR approach reduces bias due to confounding factors and reverse causality, thereby improving causal inference. We used GWASs with large sample sizes derived from large biobanks and consortia, thereby enabling the detection of light-to-moderate associations. Various MR methods were employed, and sensitivity analyses were performed to explore the possible biases due to pleiotropy. Our samples were restricted to those of European ancestry, thereby minimizing bias resulting from different genetic backgrounds. Bibliometric analysis provides an overview of research outputs, collaborative networks, and emerging trends, which reveals the scientific dynamics and landscapes in related fields. Exploring shared topics using an interdisciplinary approach may provide methodological insights and valuable perspectives for future research.

This study has some limitations. First, owing to the small number of GWASs on some personality and psychiatric traits, the exclusion criteria for SNPs were moderately relaxed to maximize the strength of the IVs; this relaxation of the exclusion criteria might have weakened the robustness of the results of some analyses. Although limited by the lack of SNPs in SCLC, we only investigated the causal effects of SCLC on extraversion and insomnia. The included populations were overwhelmingly European; therefore, these findings may not apply to other races. Additionally, the sample size of each lung cancer subtype may influence the outcomes, despite our selection of large-sample GWAS datasets for the analysis. Second, although various tools were used for science mapping, some secondary topics may have been omitted. There is a possibility of missing articles, although this does not affect the validity of the bibliometric analysis.

In conclusion, we found that genetically predicted schizophrenia is a risk factor for LC, whereas genetically predicted LC is associated with an increased risk of ADHD. No genetically predicted bidirectional causality was found between LC and its subtypes and neuroticism, extraversion, agreeableness, conscientiousness, openness, ADHD, MDD, ASD, BD, or insomnia. Science mappings provide a complementary perspective to MR analysis. Further studies should explore the potential biological mechanisms by which schizophrenia affects LC initiation and those by which LC increases the risk of ADHD.

## Data Availability

The original contributions presented in the study are included in the article/**Supplementary Material**. Further inquiries can be directed to the corresponding authors.
