# Supplementary material for: Causal effects between personality and psychiatric traits and lung cancer: a bidirectional two-sample Mendelian randomization and bibliometric study
Source: Front Psychiatry. 2024 Sep 12;15:1338481. doi: 10.3389/fpsyt.2024.1338481 (PMC11424467; doi:10.3389/fpsyt.2024.1338481)
Supplement: Supplementary file 1 [file Table1.docx]

**Contents**

**Supplementary Figures**

**Figure S1.** Core assumptions model of MR analysis……………………………………………….……2

**Figure S2.** Scatter plots of forward MR analysis from genetically predicted personality/psychiatric traits on LC/subtypes risk………….………………………………………………………………………...…3

**Figure S3.** Leave-one-out plots of forward MR analysis from genetically predicted personality/psychiatric traits on LC/subtypes risk………………………………………………………..9

**Figure S4.** Funnel plots of forward MR analysis from genetically predicted personality/psychiatric traits on LC/subtypes risk…….......…………………………………………………………………………...15

**Figure S5.** Scatter plots of reverse MR analysis from genetically predicted LC/subtypes on personality/psychiatric traits risk…………………………………………………………………….…21

**Figure S6.** Leave-one-out plots of reverse MR analysis from genetically predicted LC/subtypes on personality/psychiatric traits risk…...…………………………………………………………………...25

**Figure S7.** Funnel plots of reverse MR analysis from genetically predicted LC/subtypes on personality/psychiatric traits risk…….............……………………………………………………….....28

**Figure S8.** Supplementary science mapping using Scopus and PubMed databases……………………..................................................................................................................32

**Abbreviations:** ADHD, attention-deficit/hyperactivity disorder; ASD, autism spectrum disorder; BD, bipolar disorder; IV, instrumental variable; LC, lung cancer; LUAD, lung adenocarcinoma; LUSC, lung squamous cell carcinoma; MDD, major depressive disorder; MR, Mendelian randomization; SCLC, small cell lung cancer; SNP, single nucleotide polymorphism; TC, total citations; WoSCC, Web of Science Core Collection

**Figure S1.** Core assumptions model of MR analysis.


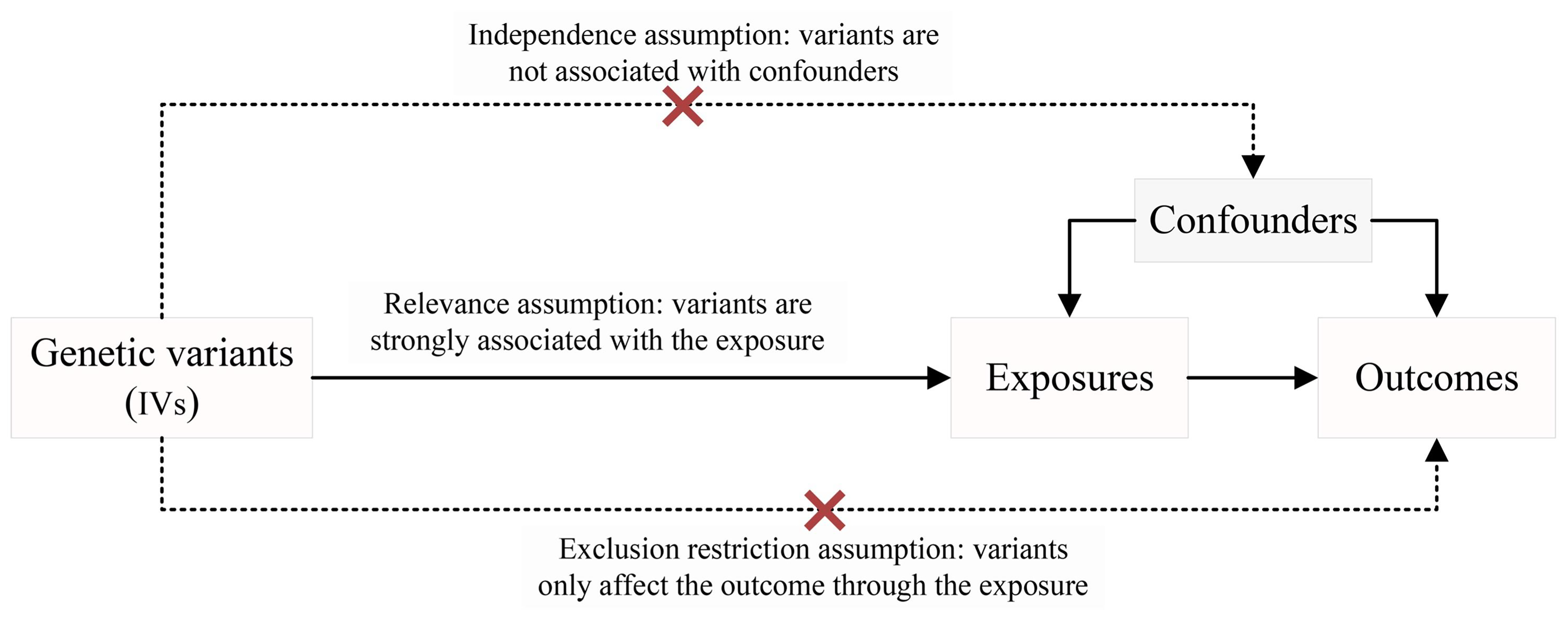


**Figure S2.** Scatter plots of forward MR analysis from genetically predicted personality/psychiatric traits on LC/subtypes risk.

**Figure S2-A.** Scatter plots from genetically predicted neuroticism on the risk of (a) LC; (b) LUSC; (c) LUAD; (d) SCLC.


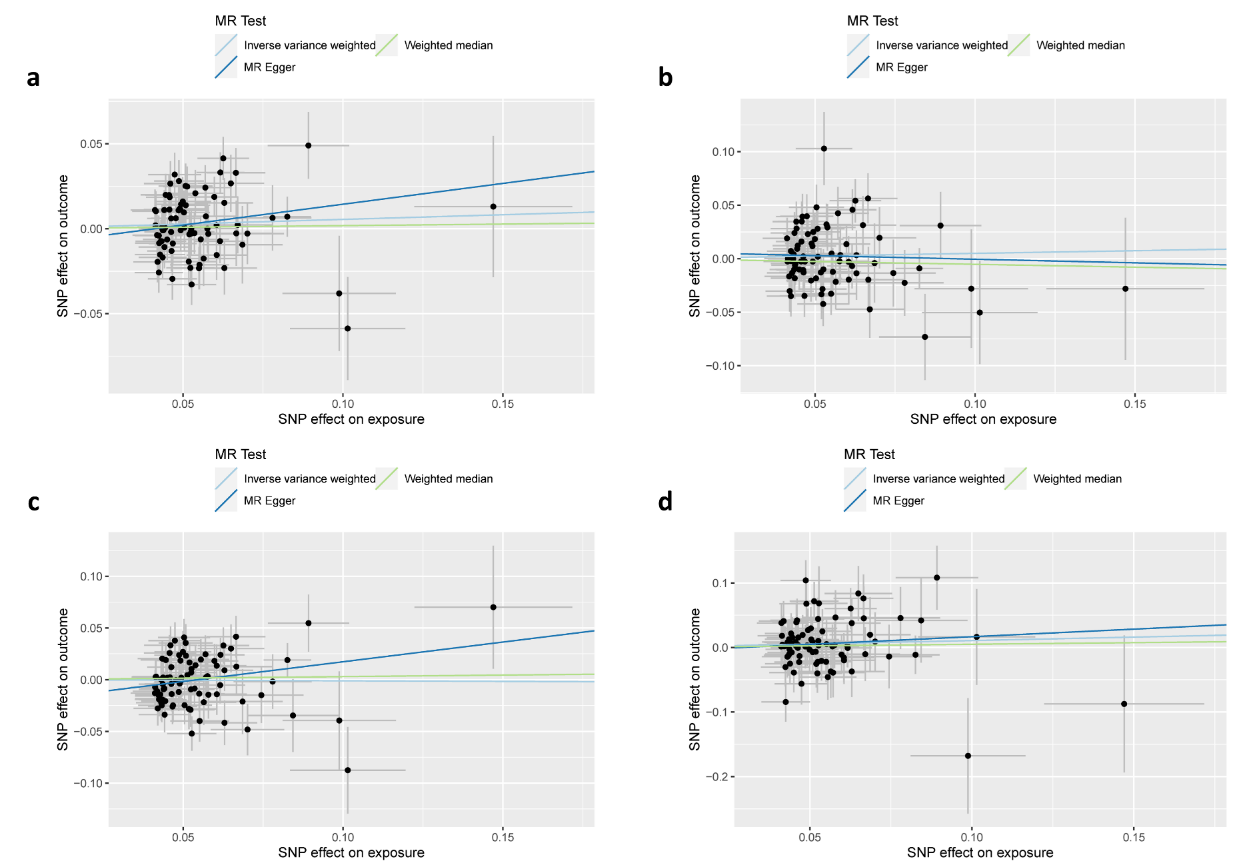


**Figure S2-B.** Scatter plots from genetically predicted extraversion on the risk of (a) LC; (b) LUSC; (c) LUAD; (d) SCLC.


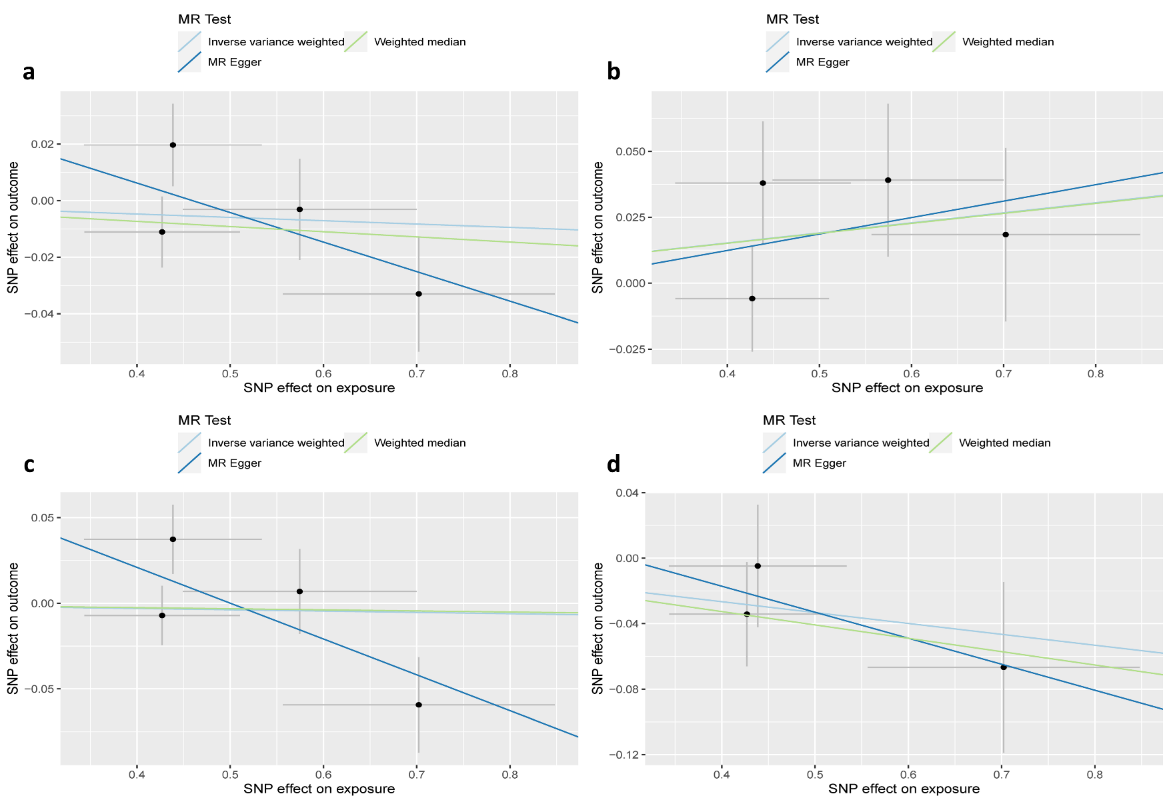


**Figure S2-C.** Scatter plots from genetically predicted agreeableness on the risk of (a) LC; (b) LUSC; (c) LUAD; (d) SCLC.


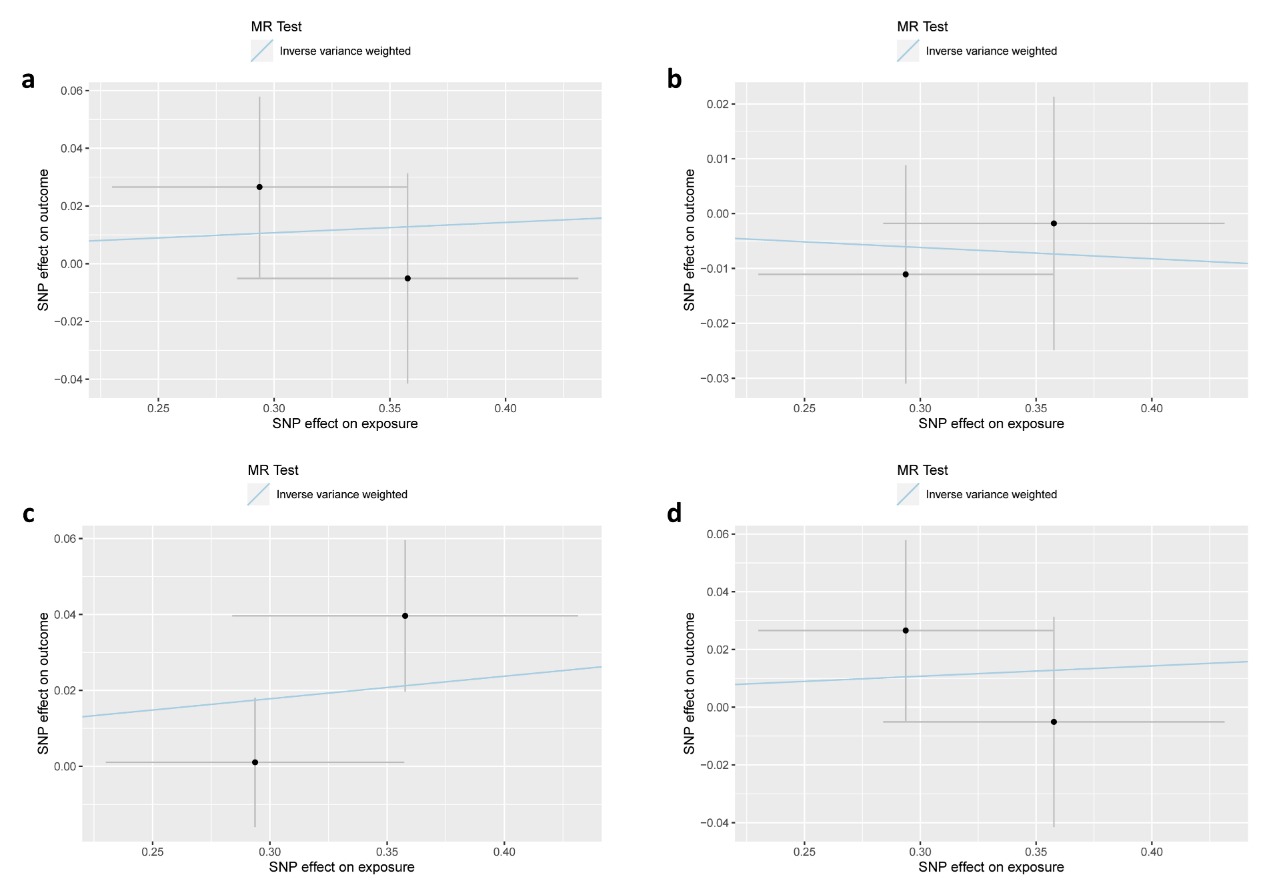


**Figure S2-D.** Scatter plots from genetically predicted conscientiousness on the risk of (a) LC; (b) LUSC; (c) LUAD; (d) SCLC.


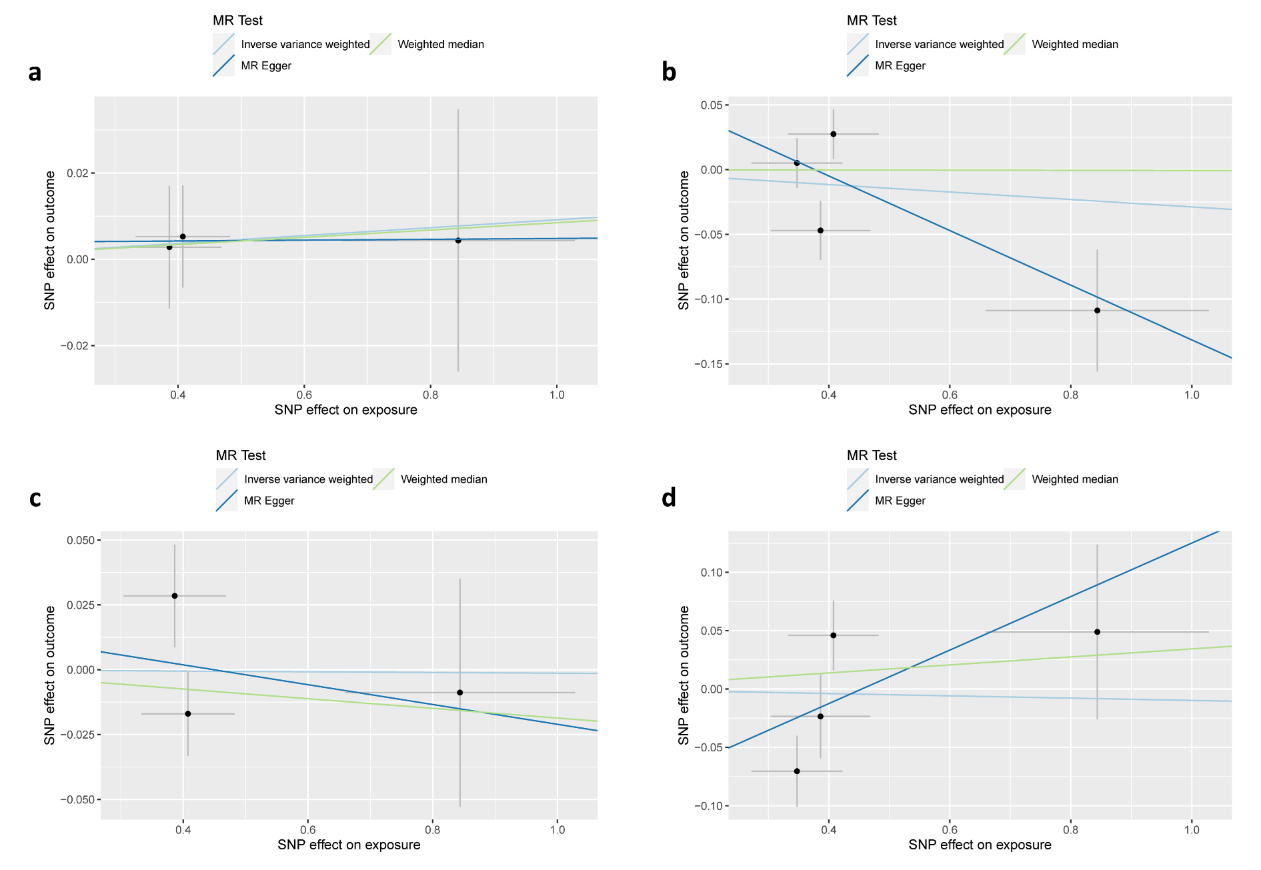


**Figure S2-E.** Scatter plots from genetically predicted openness on the risk of (a) LC; (b) LUSC; (c) LUAD; (d) SCLC.


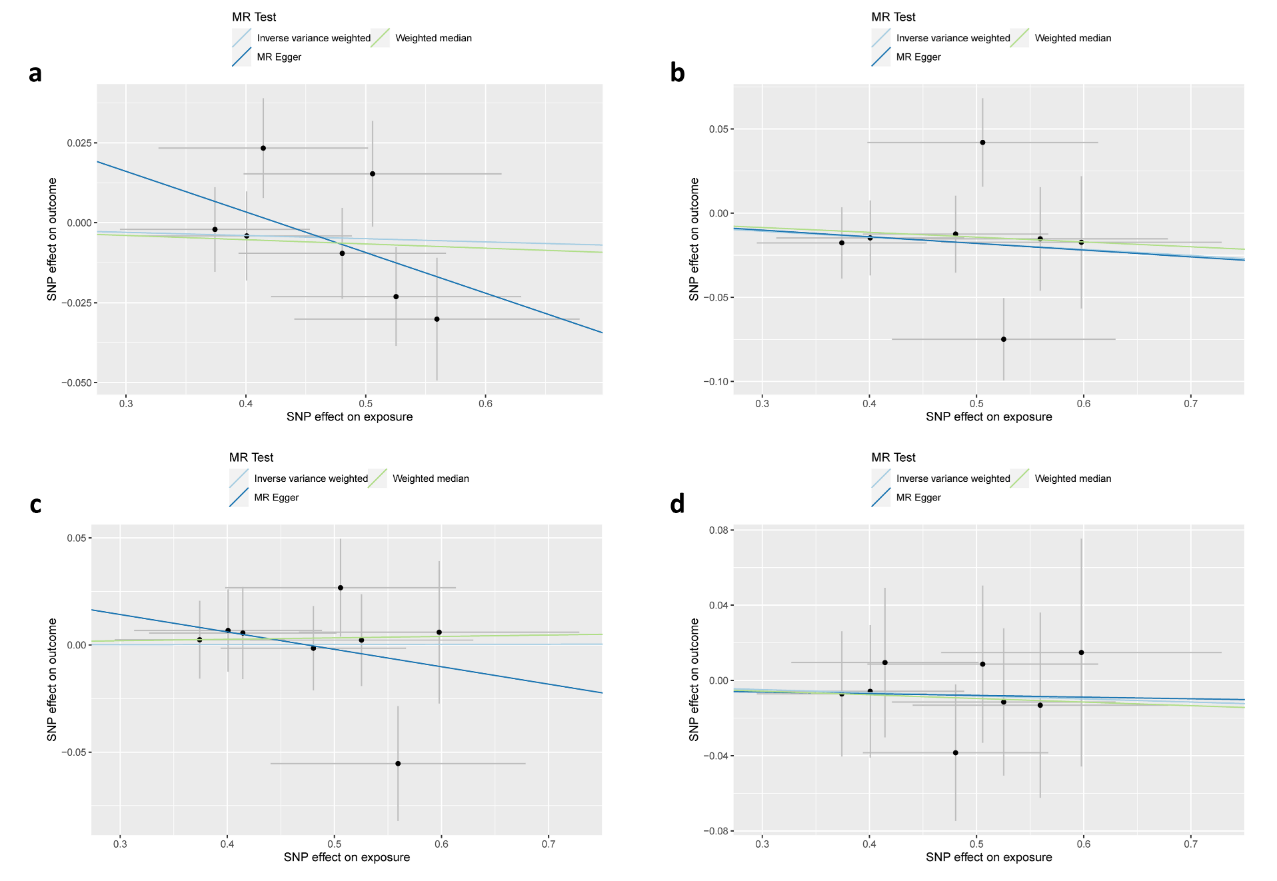


**Figure S2-F.** Scatter plots from genetically predicted schizophrenia on the risk of (a) LC; (b) LUSC; (c) LUAD; (d) SCLC.


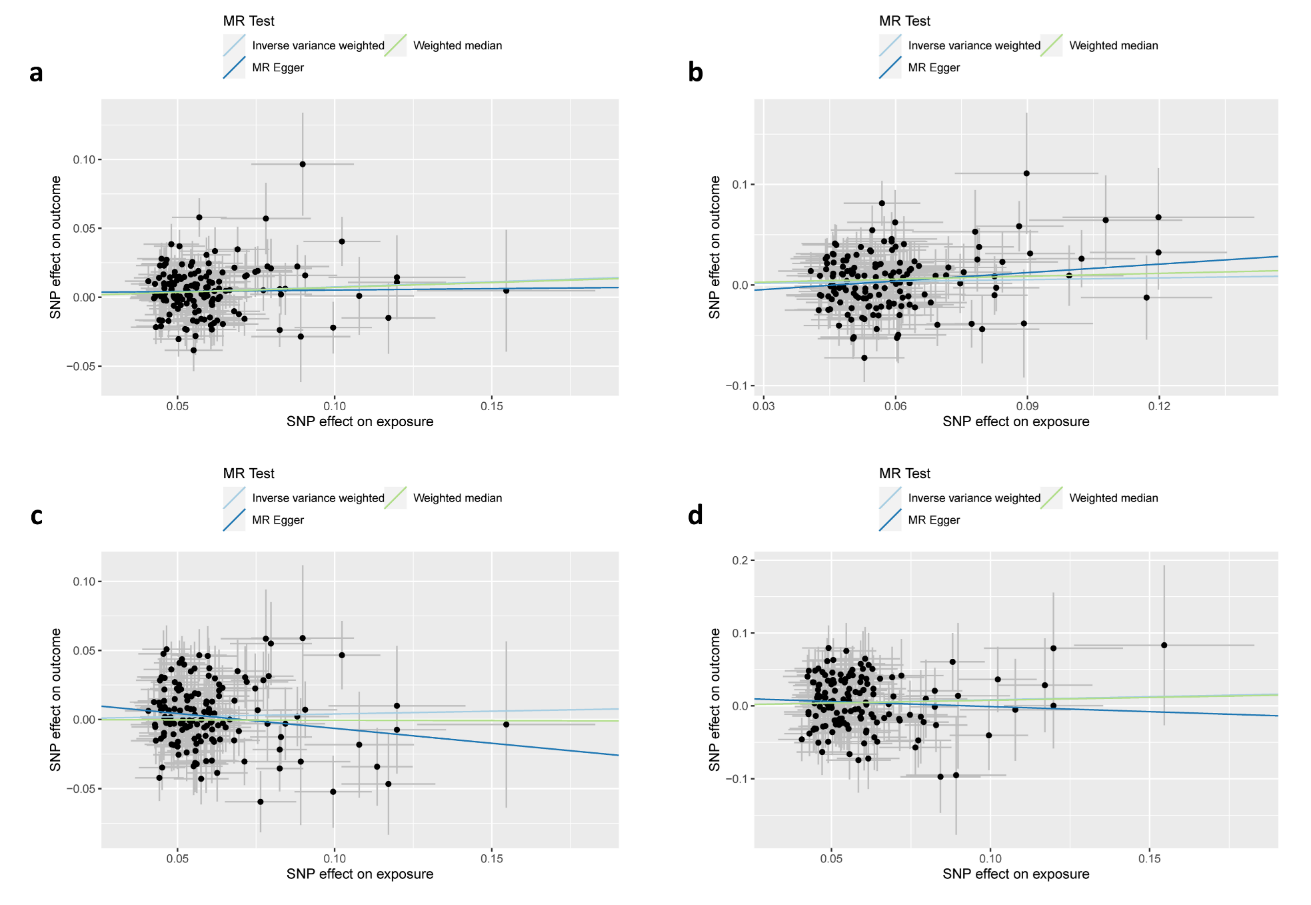


**Figure S2-G.** Scatter plots from genetically predicted ADHD on the risk of (a) LC; (b) LUSC; (c) LUAD; (d) SCLC.


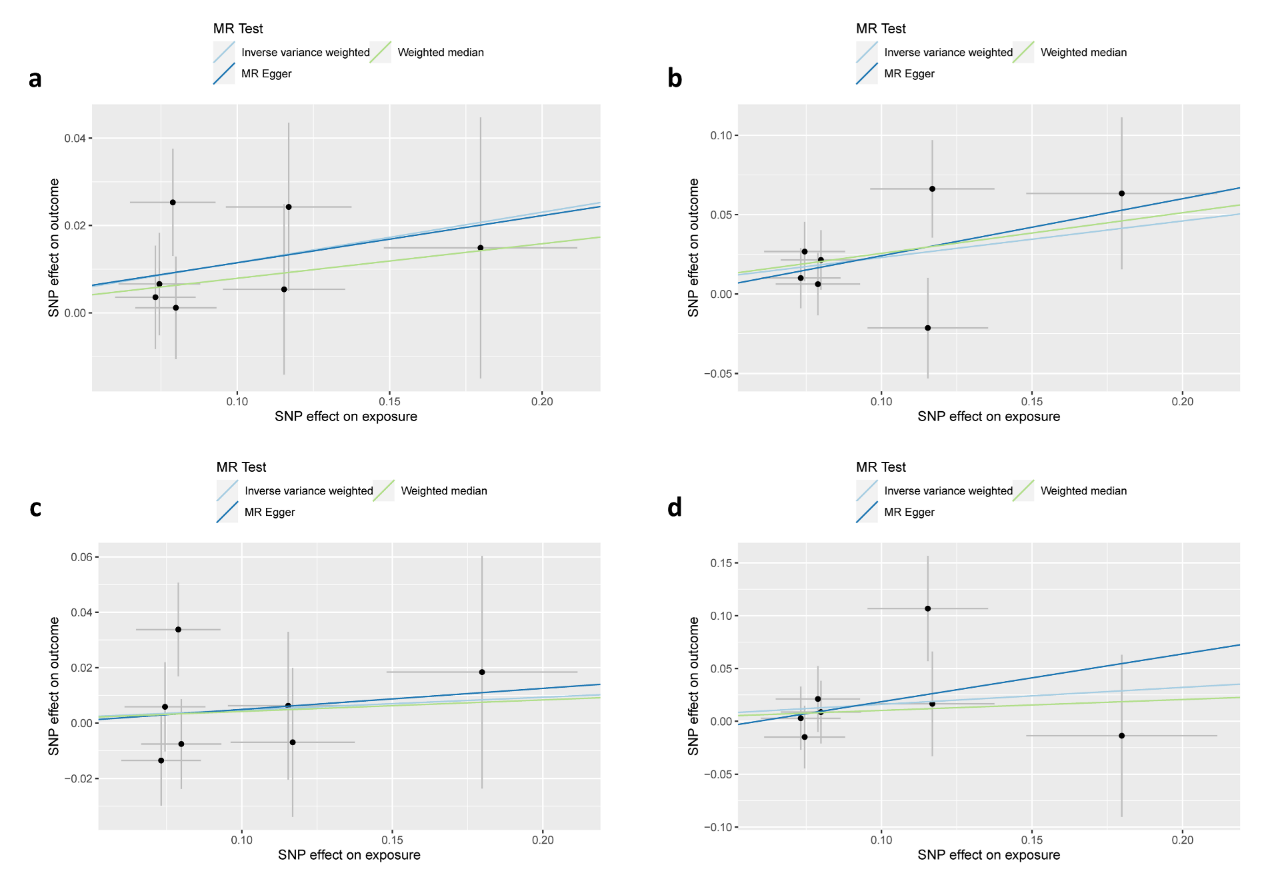


**Figure S2-H.** Scatter plots from genetically predicted MDD on the risk of (a) LC; (b) LUSC; (c) LUAD; (d) SCLC.


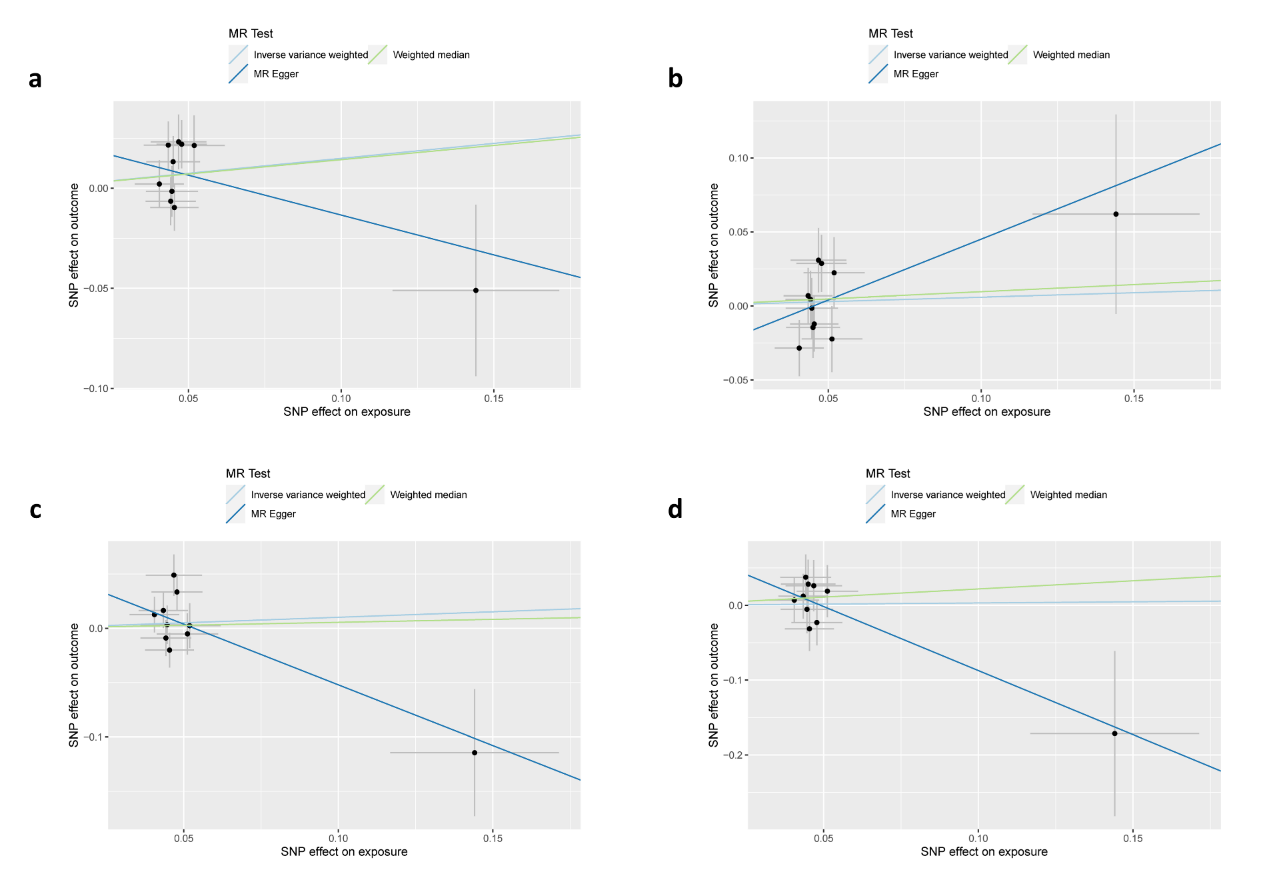


**Figure S2-I.** Scatter plots from genetically predicted ASD on the risk of (a) LC; (b) LUSC; (c) LUAD; (d) SCLC.


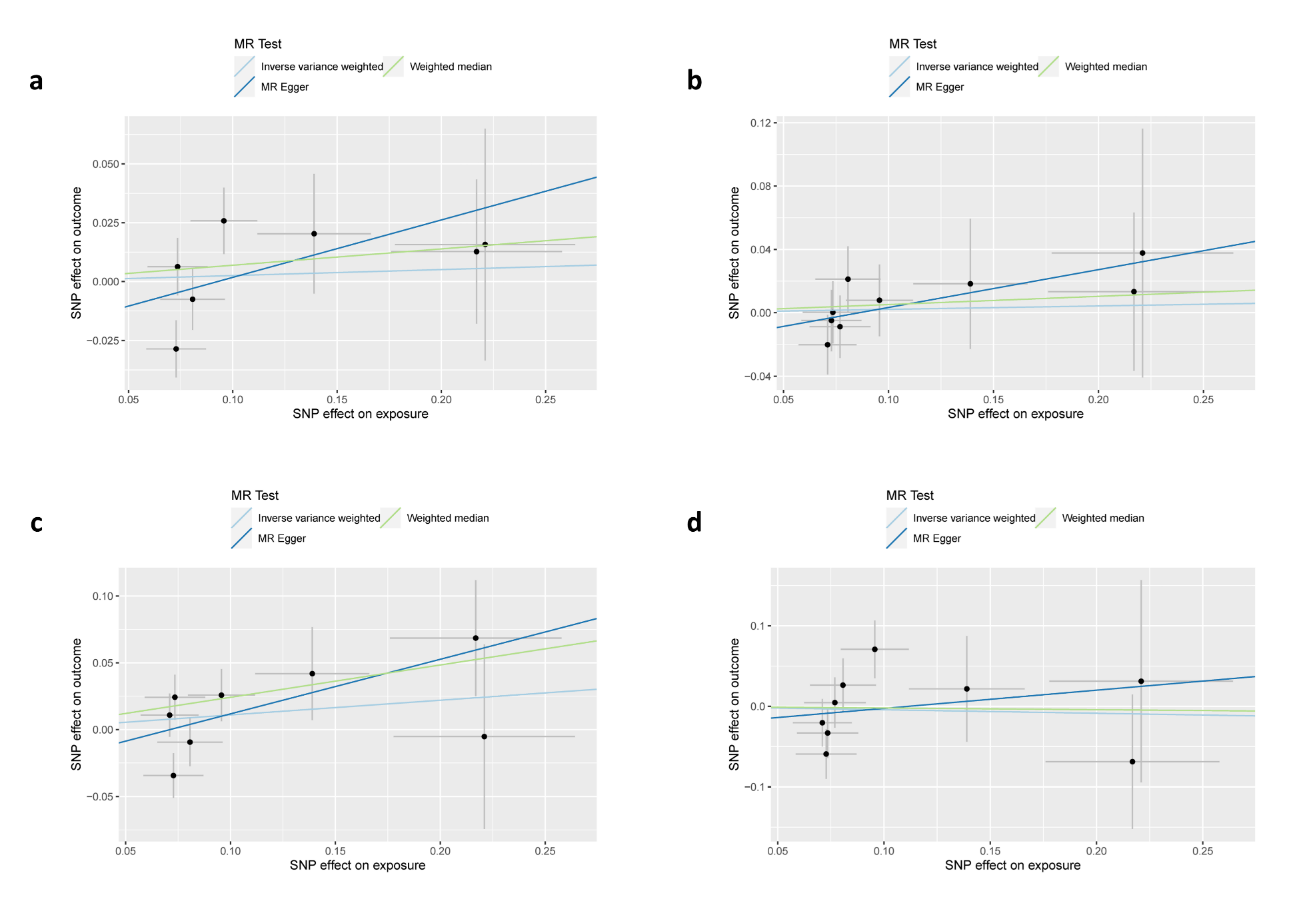


**Figure S2-J.** Scatter plots from genetically predicted BD on the risk of (a) LC; (b) LUSC; (c) LUAD; (d) SCLC.


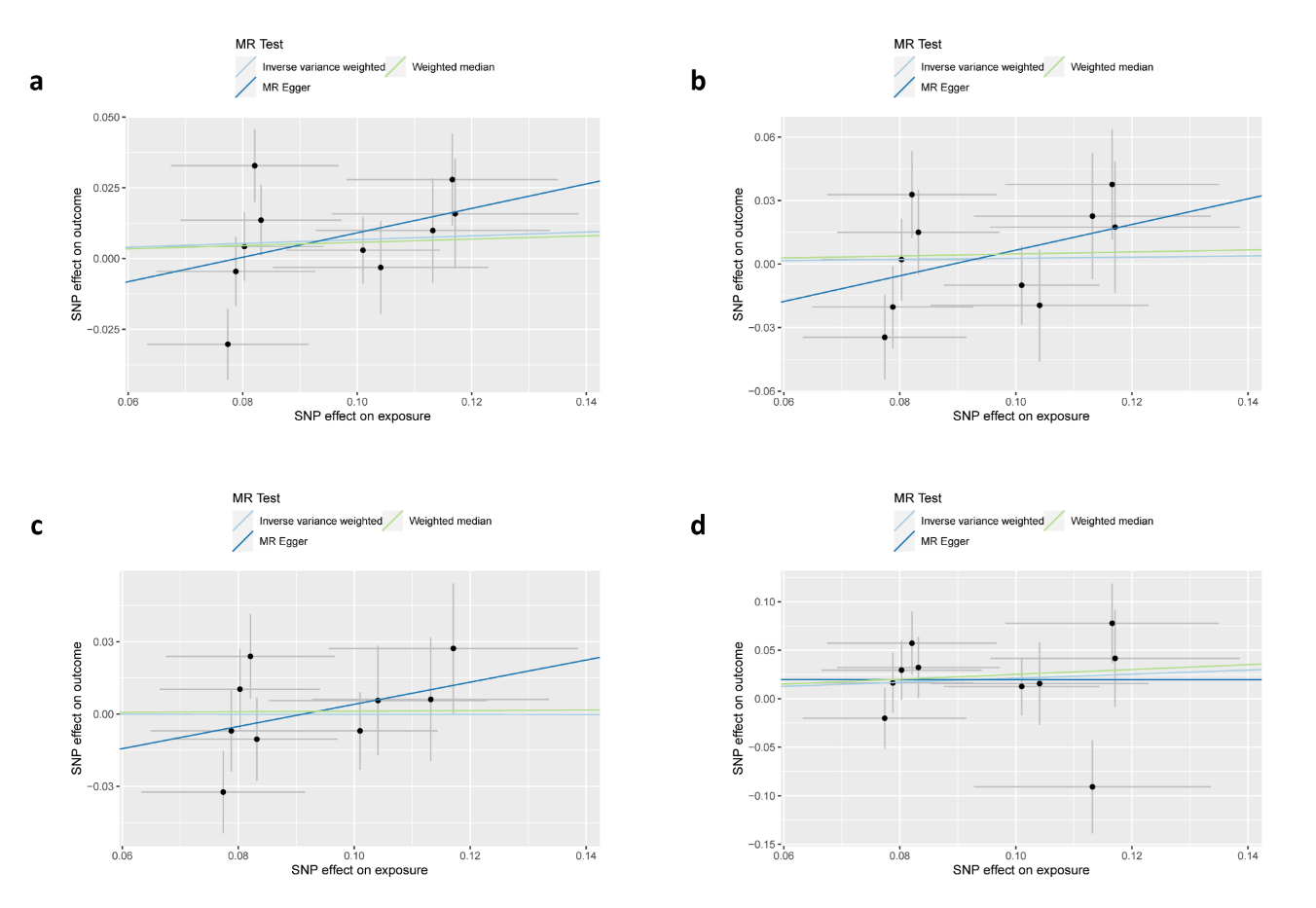


**Figure S2-K.** Scatter plots from genetically predicted insomnia on the risk of (a) LC; (b) LUSC; (c) LUAD; (d) SCLC.


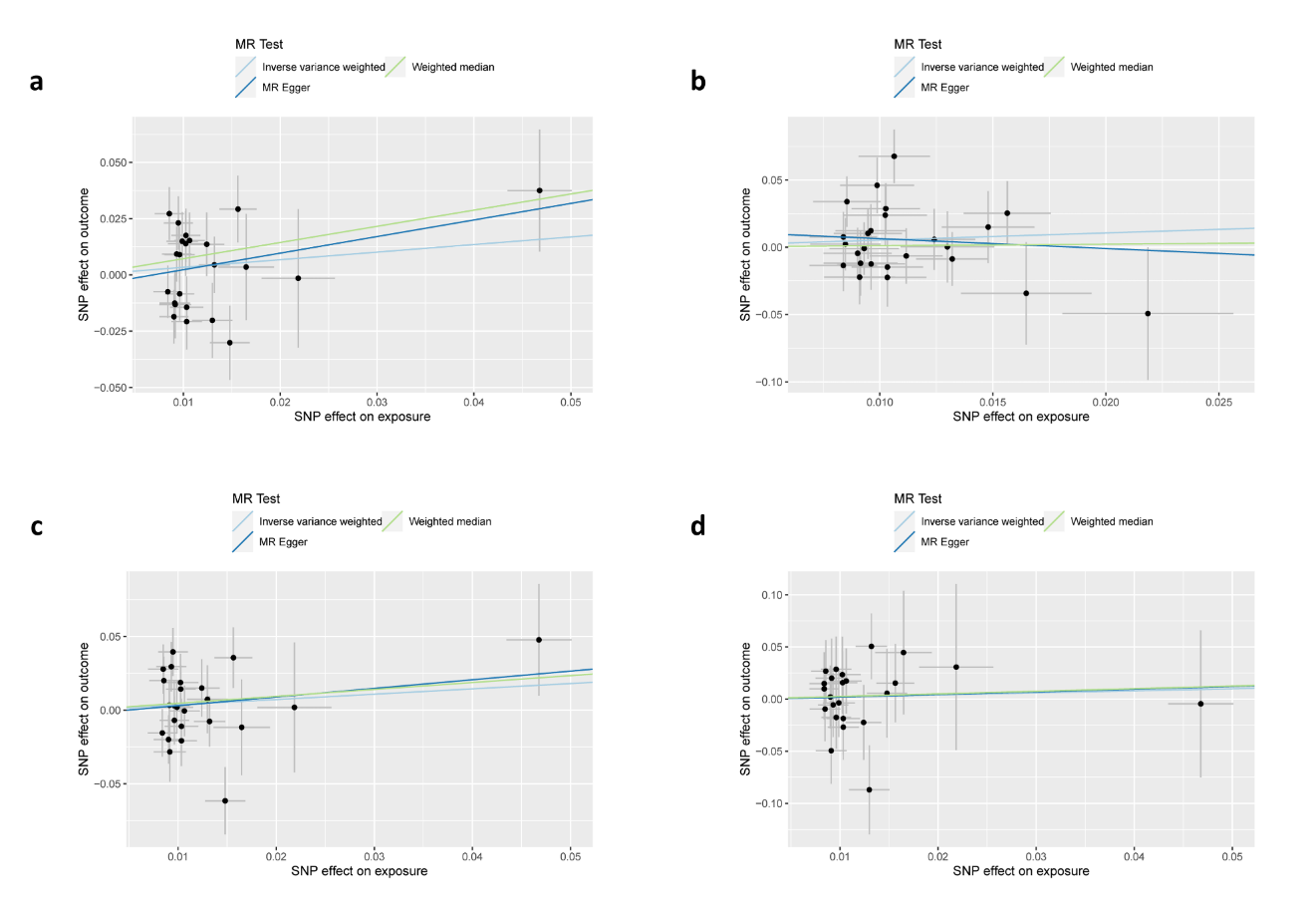


**Figure S2-L.** Scatter plots from genetically predicted anxiety on the risk of (a) LC; (b) LUSC; (c) LUAD; (d) SCLC.

**
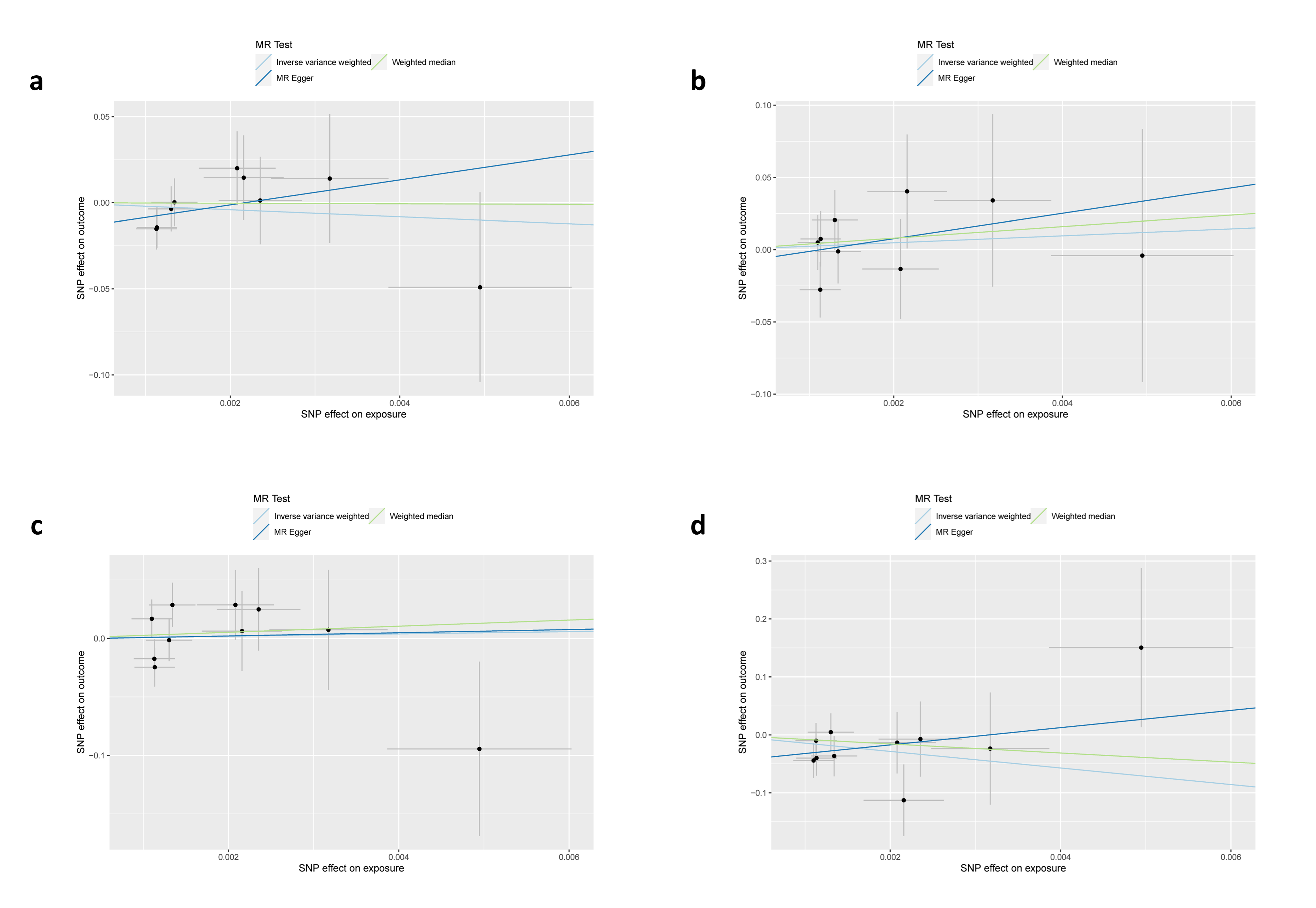
**

**Figure S3.** Leave-one-out plots of forward MR analysis from genetically predicted personality/psychiatric traits on LC/subtypes risk.

**Figure S3-A.** Leave-one-out plots from genetically predicted neuroticism on the risk of (a) LC; (b) LUSC; (c) LUAD; (d) SCLC.


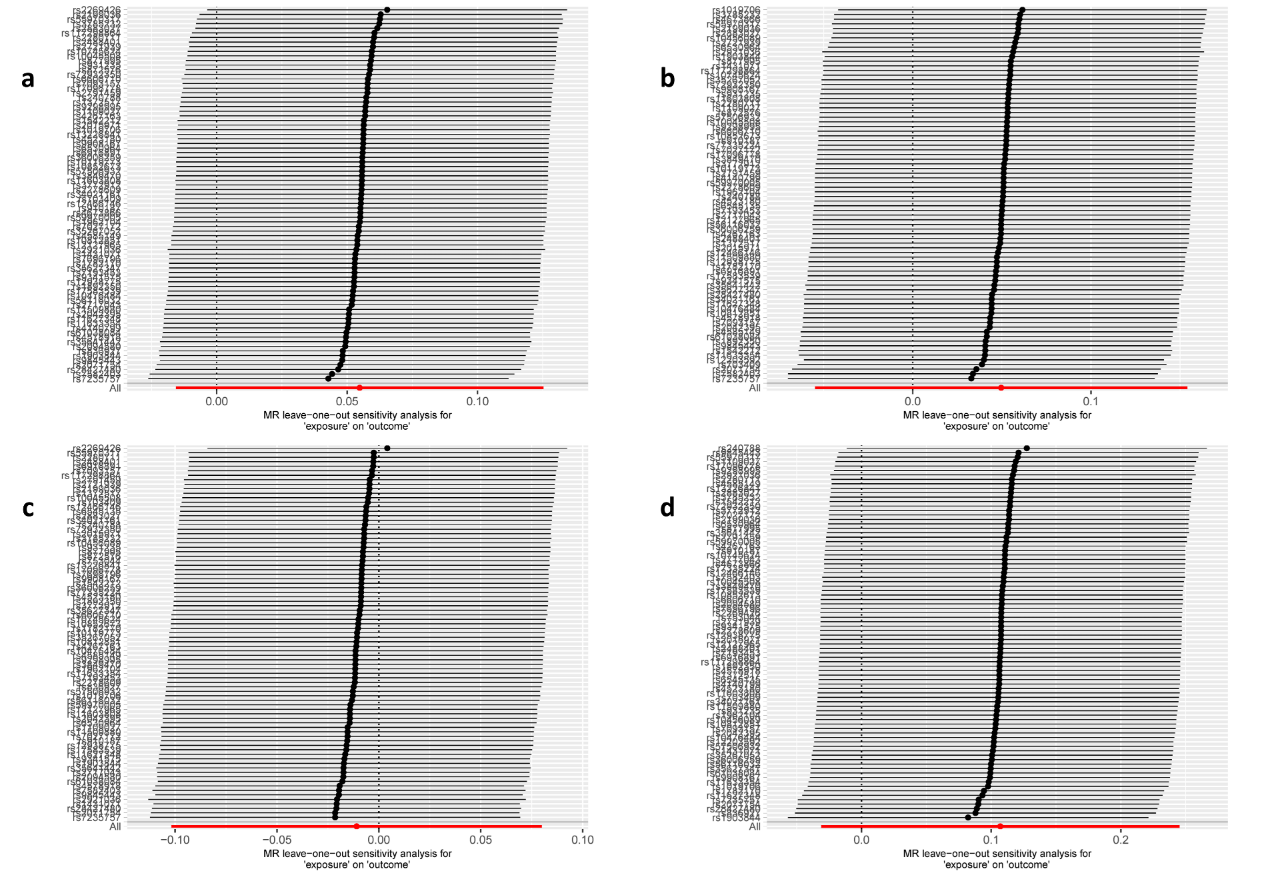


**Figure S3-B.** Leave-one-out plots from genetically predicted extraversion on the risk of (a) LC; (b) LUSC; (c) LUAD; (d) SCLC.


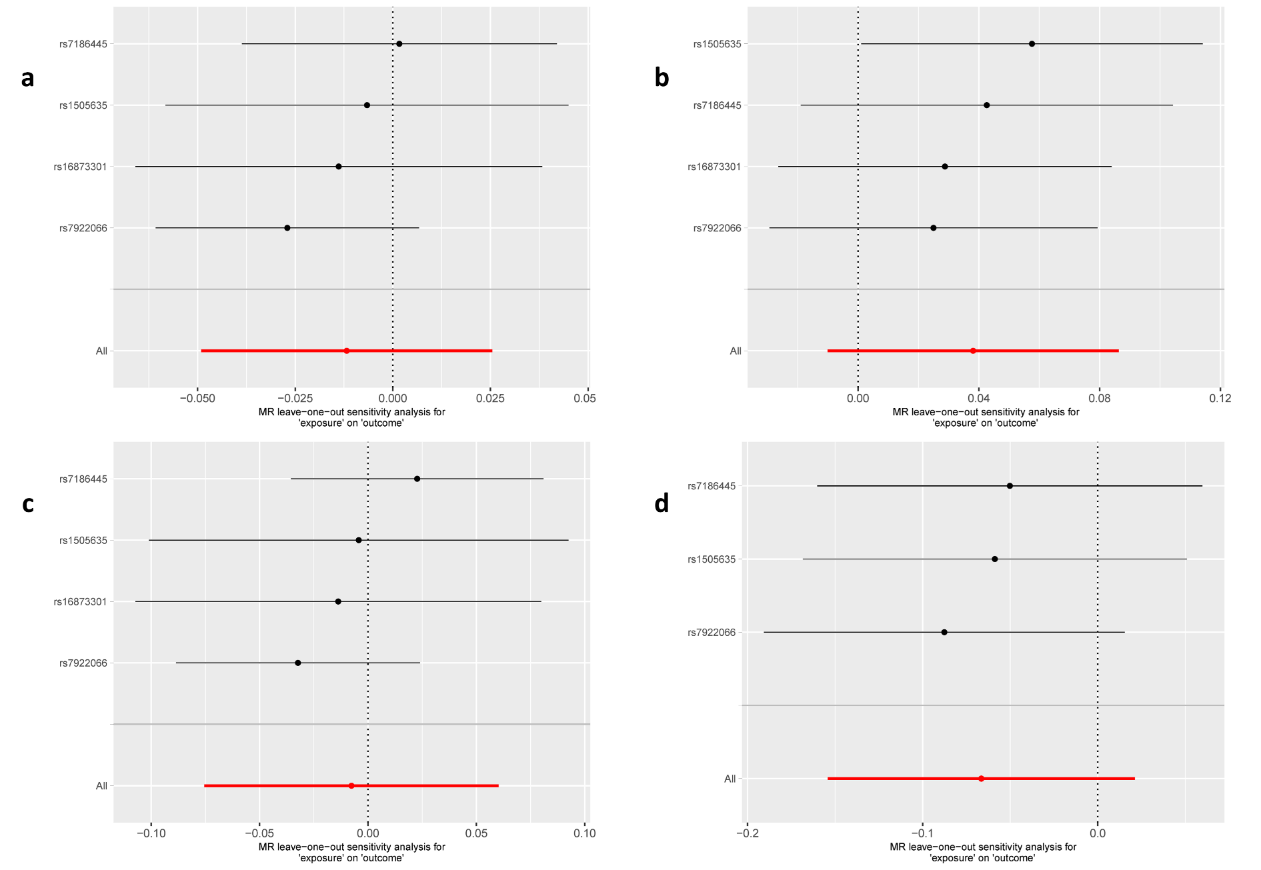


**Figure S3-C.** Leave-one-out plots from genetically predicted conscientiousness on the risk of (a) LC; (b) LUSC; (c) LUAD; (d) SCLC.


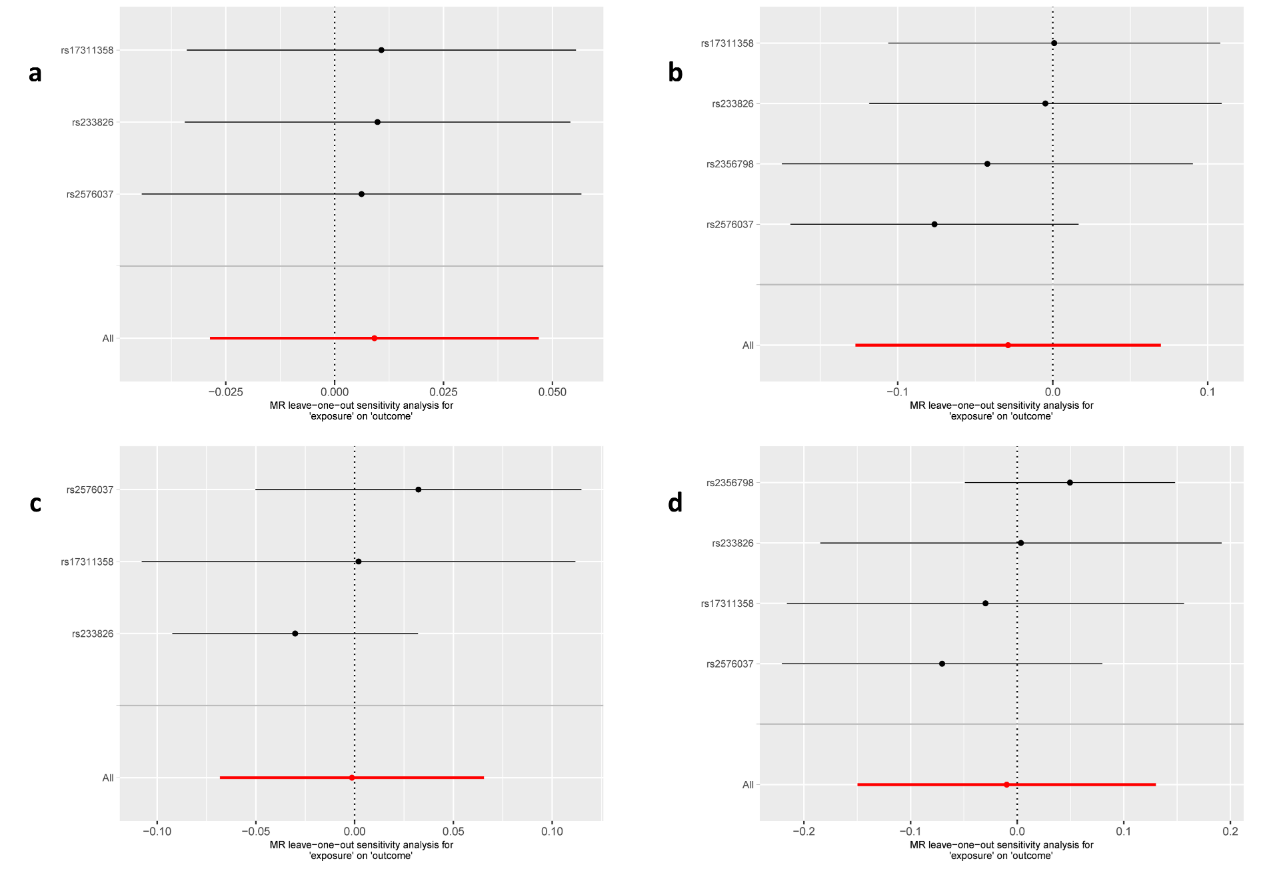


**Figure S3-D.** Leave-one-out plots from genetically predicted openness on the risk of (a) LC; (b) LUSC; (c) LUAD; (d) SCLC.


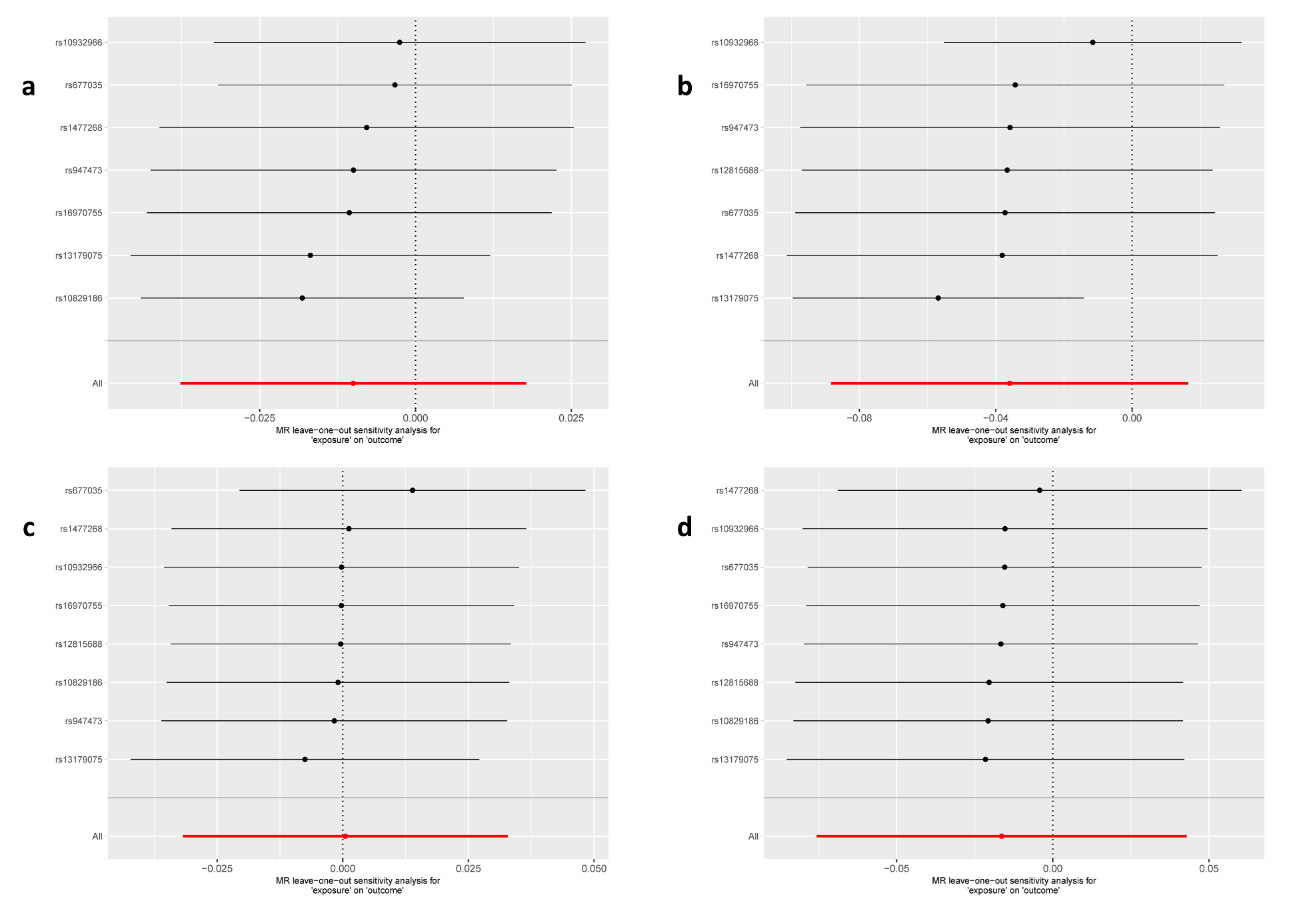


**Figure S3-E.** Leave-one-out plots from genetically predicted schizophrenia on the risk of (a) LC; (b) LUSC; (c) LUAD; (d) SCLC.


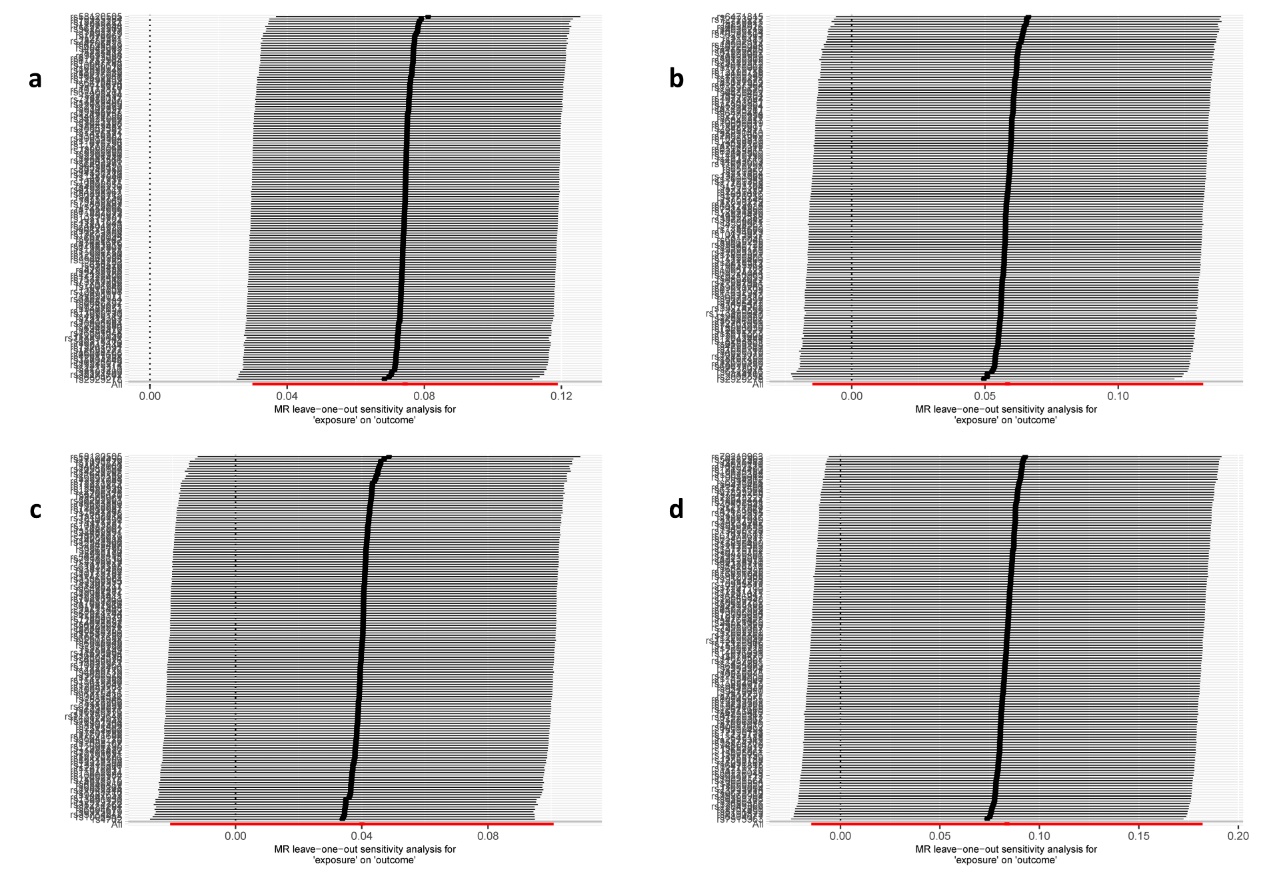


**Figure S3-F.** Leave-one-out plots from genetically predicted ADHD on the risk of (a) LC; (b) LUSC; (c) LUAD; (d) SCLC.


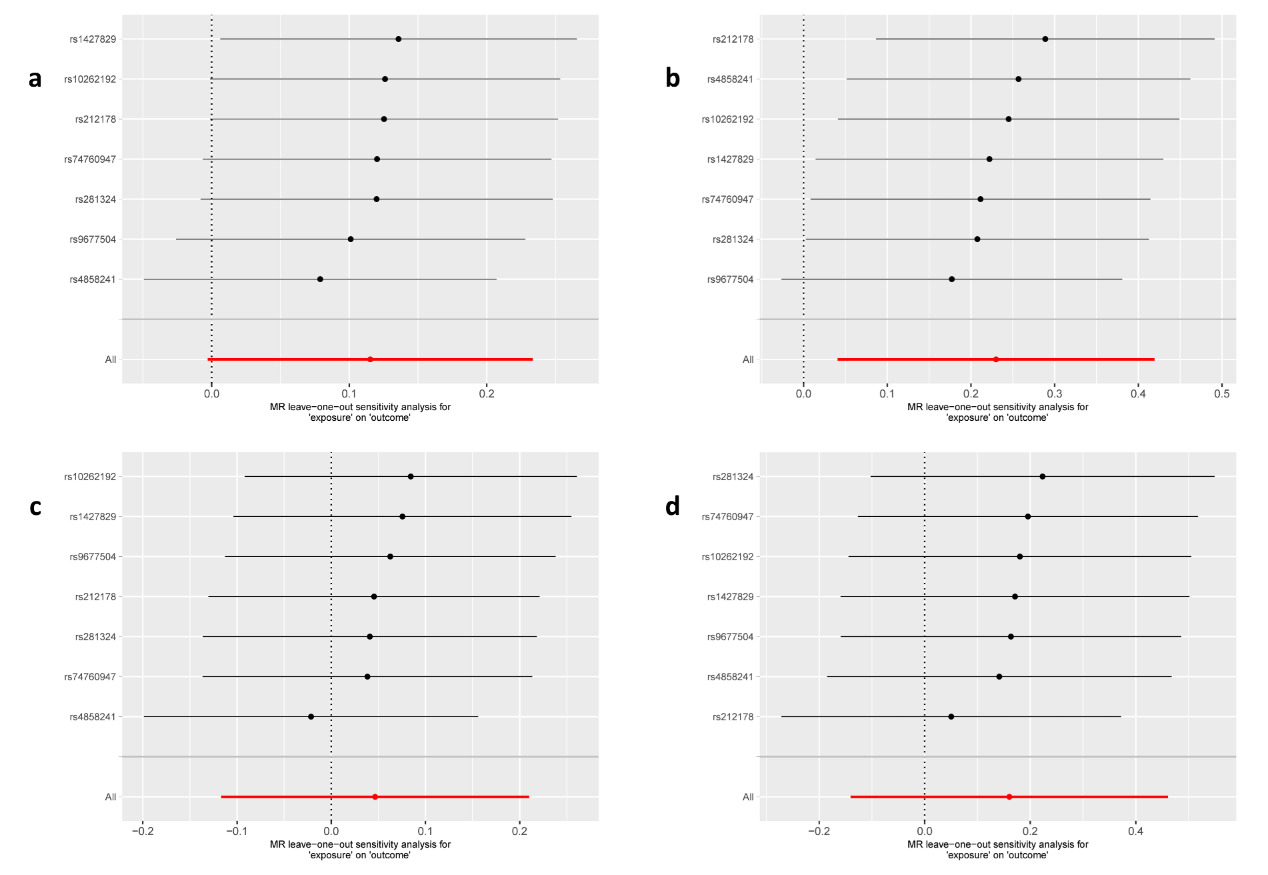


**Figure S3-G.** Leave-one-out plots from genetically predicted MDD on the risk of (a) LC; (b) LUSC; (c) LUAD; (d) SCLC.


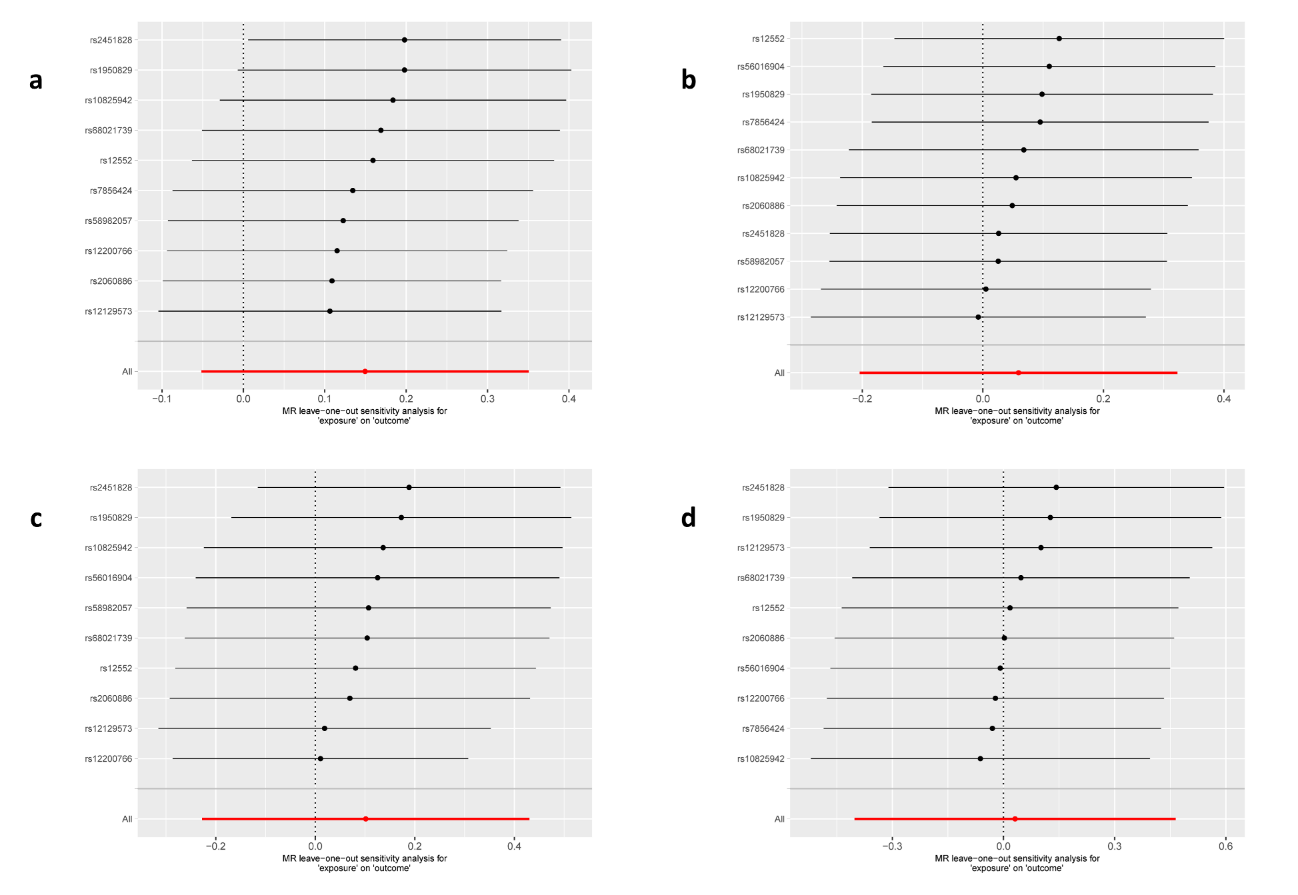


**Figure S3-H.** Leave-one-out plots from genetically predicted ASD on the risk of (a) LC; (b) LUSC; (c) LUAD; (d) SCLC.


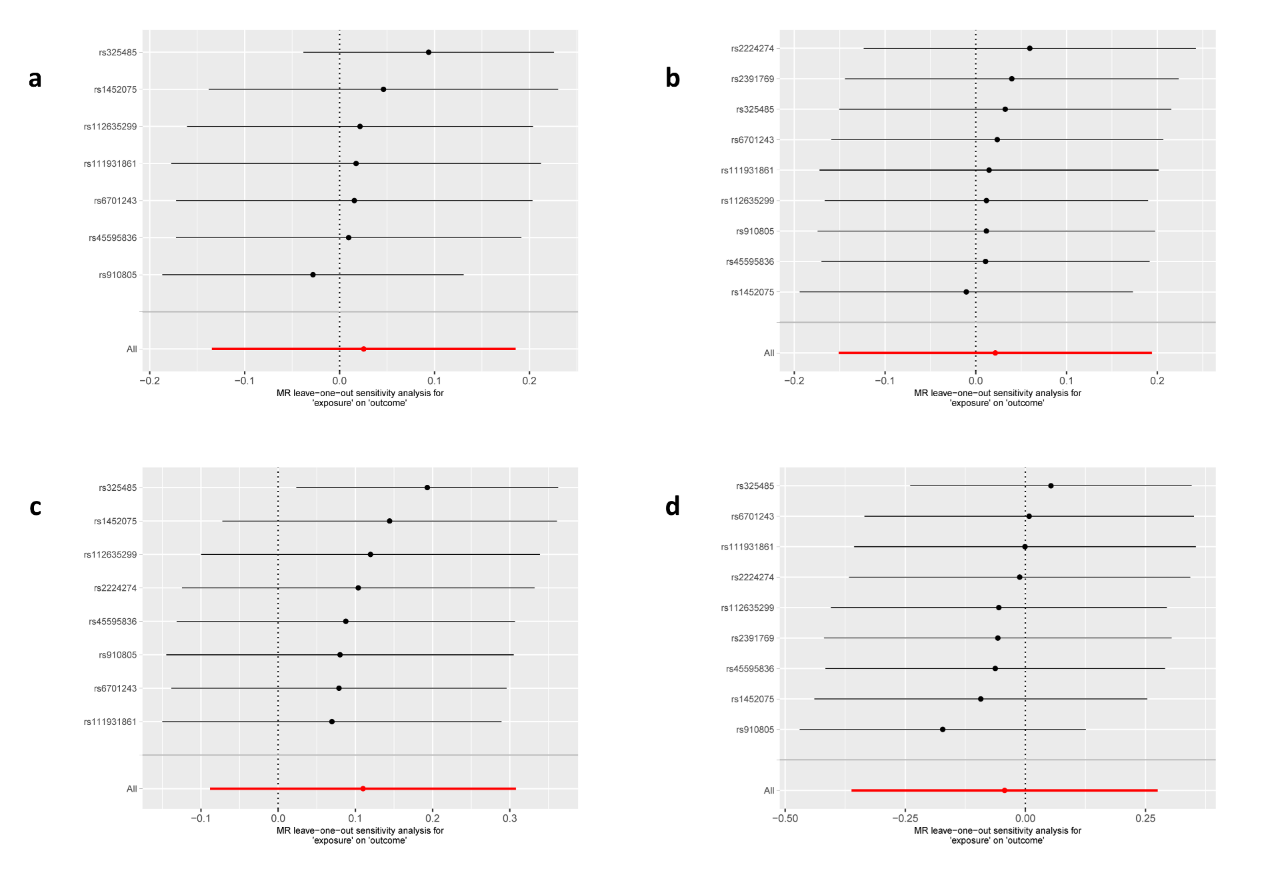


**Figure S3-I.** Leave-one-out plots from genetically predicted BD on the risk of (a) LC; (b) LUSC; (c) LUAD; (d) SCLC.


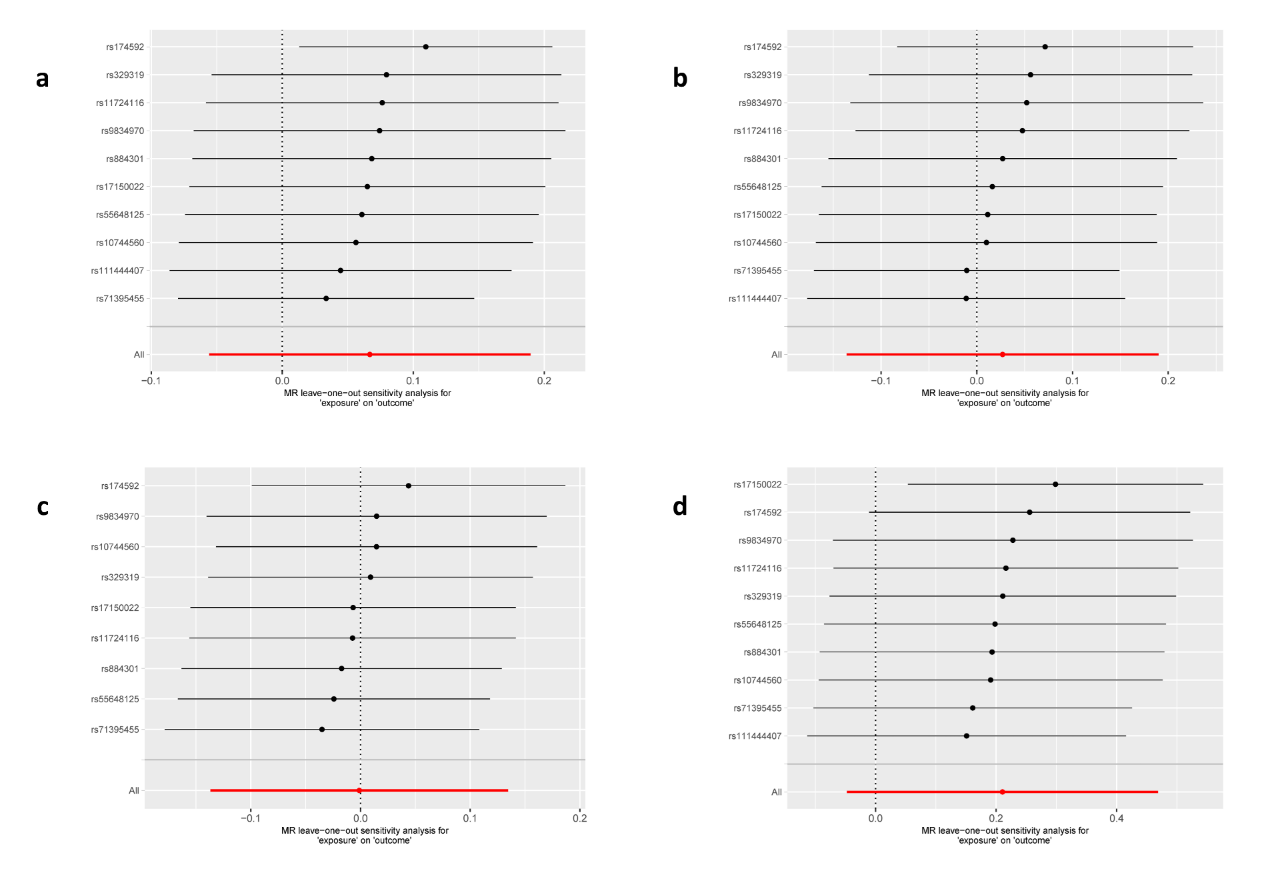


**Figure S3-J.** Leave-one-out plots from genetically predicted insomnia on the risk of (a) LC; (b) LUSC; (c) LUAD; (d) SCLC.


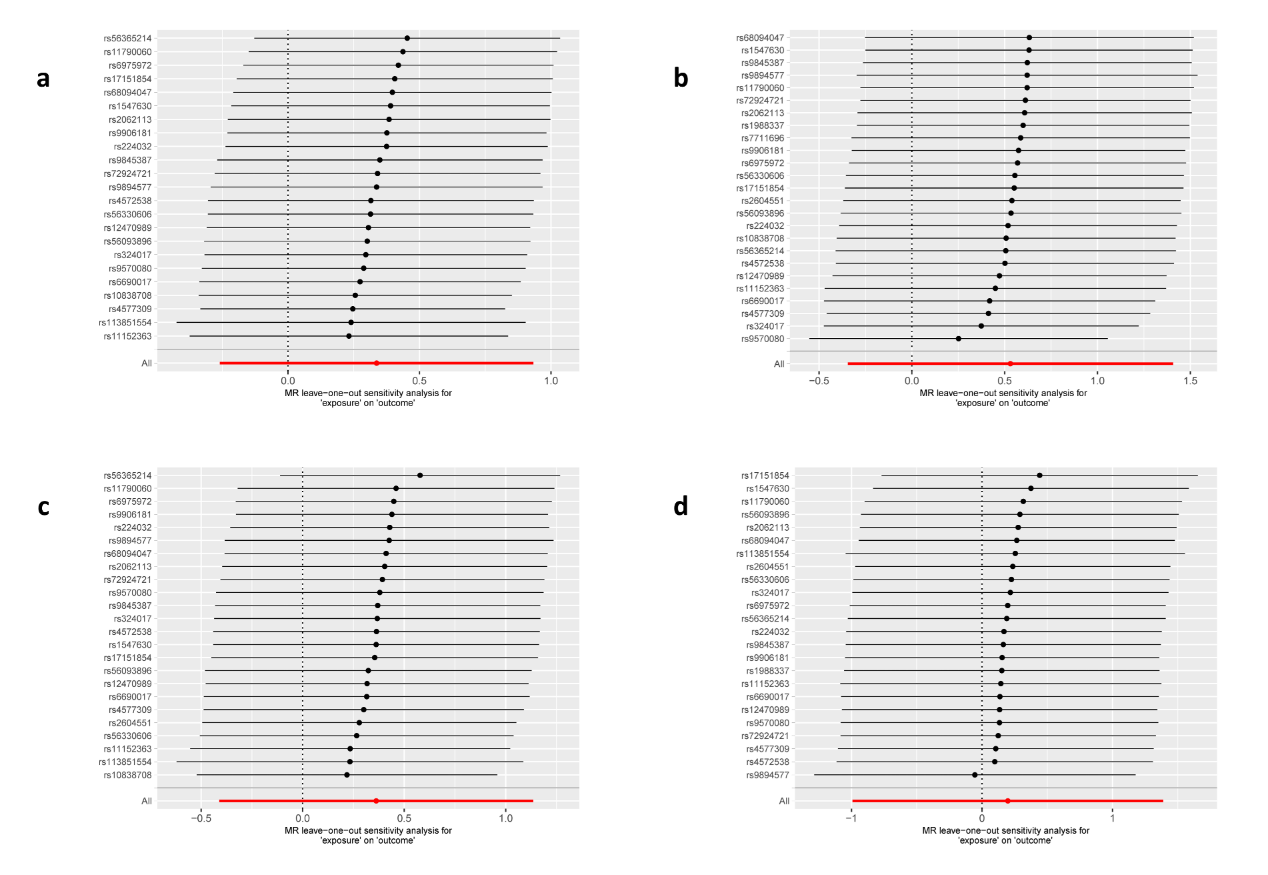


**Figure S3-K.** Leave-one-out plots from genetically predicted anxiety on the risk of (a) LC; (b) LUSC; (c) LUAD; (d) SCLC.


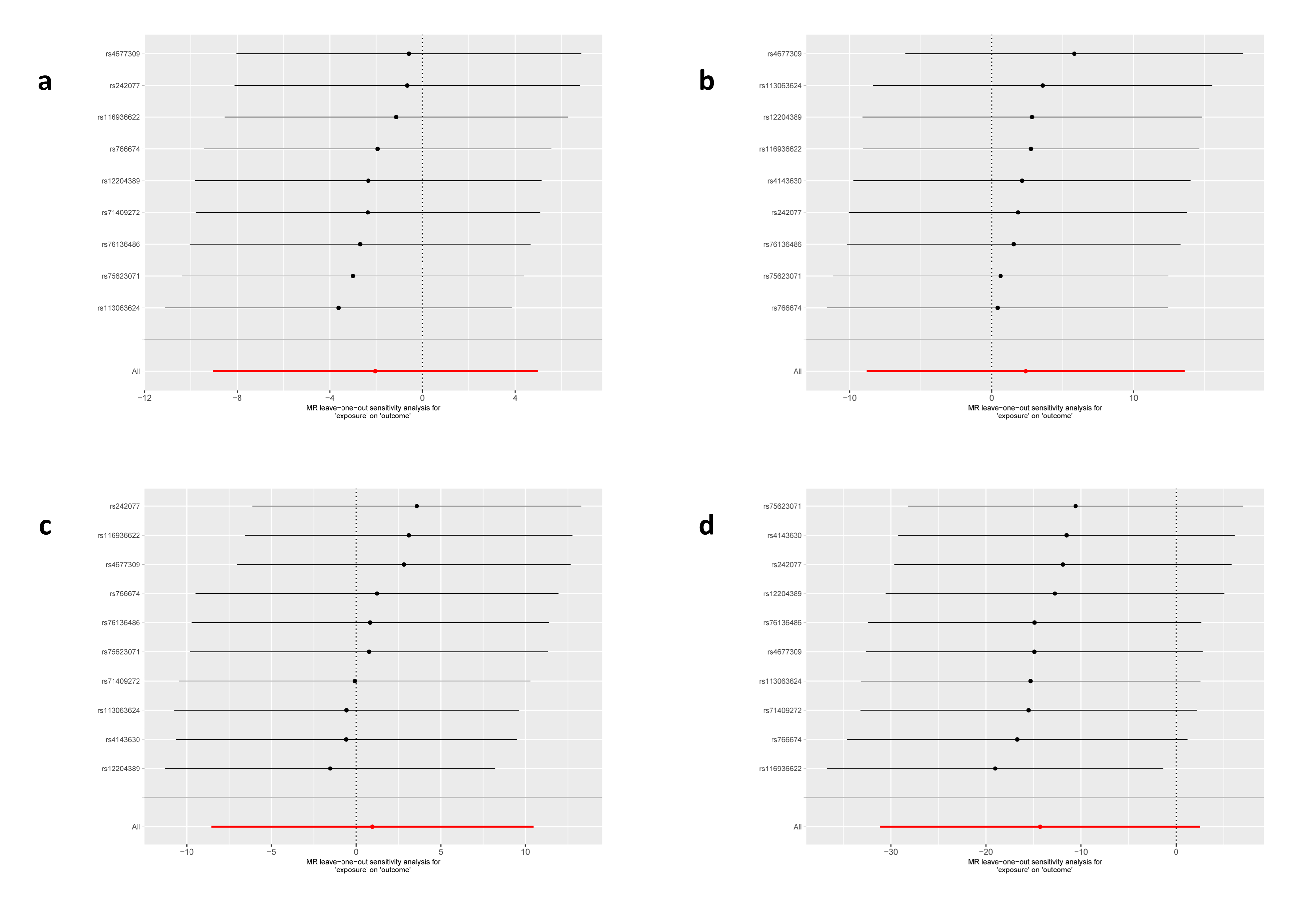


**Figure S4.** Funnel plots of forward MR analysis from genetically predicted personality/psychiatric traits on LC/subtypes risk.

**Figure S4-A.** Funnel plots from genetically predicted neuroticism on the risk of (a) LC; (b) LUSC; (c) LUAD; (d) SCLC.


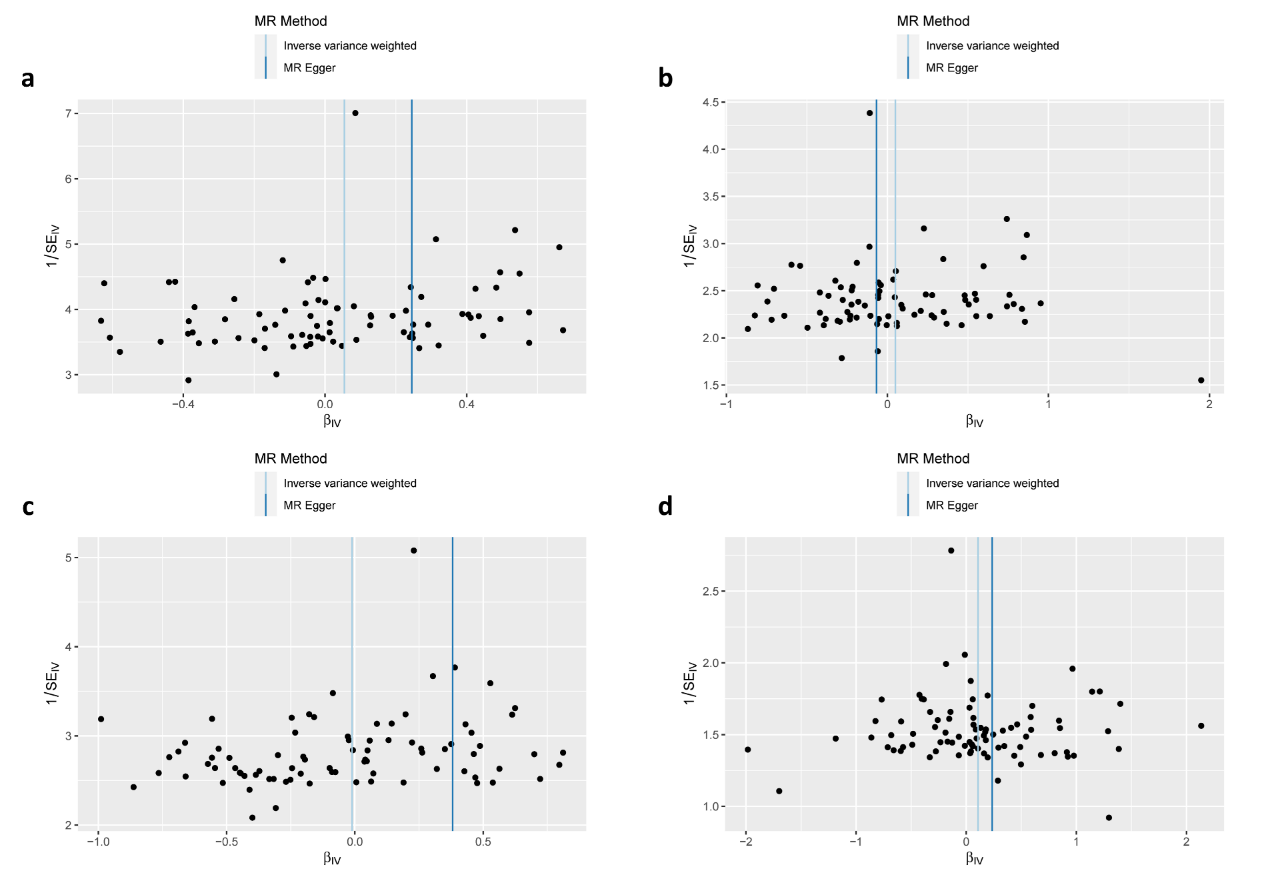


**Figure S4-B.** Funnel plots from genetically predicted extraversion on the risk of (a) LC; (b) LUSC; (c) LUAD; (d) SCLC.


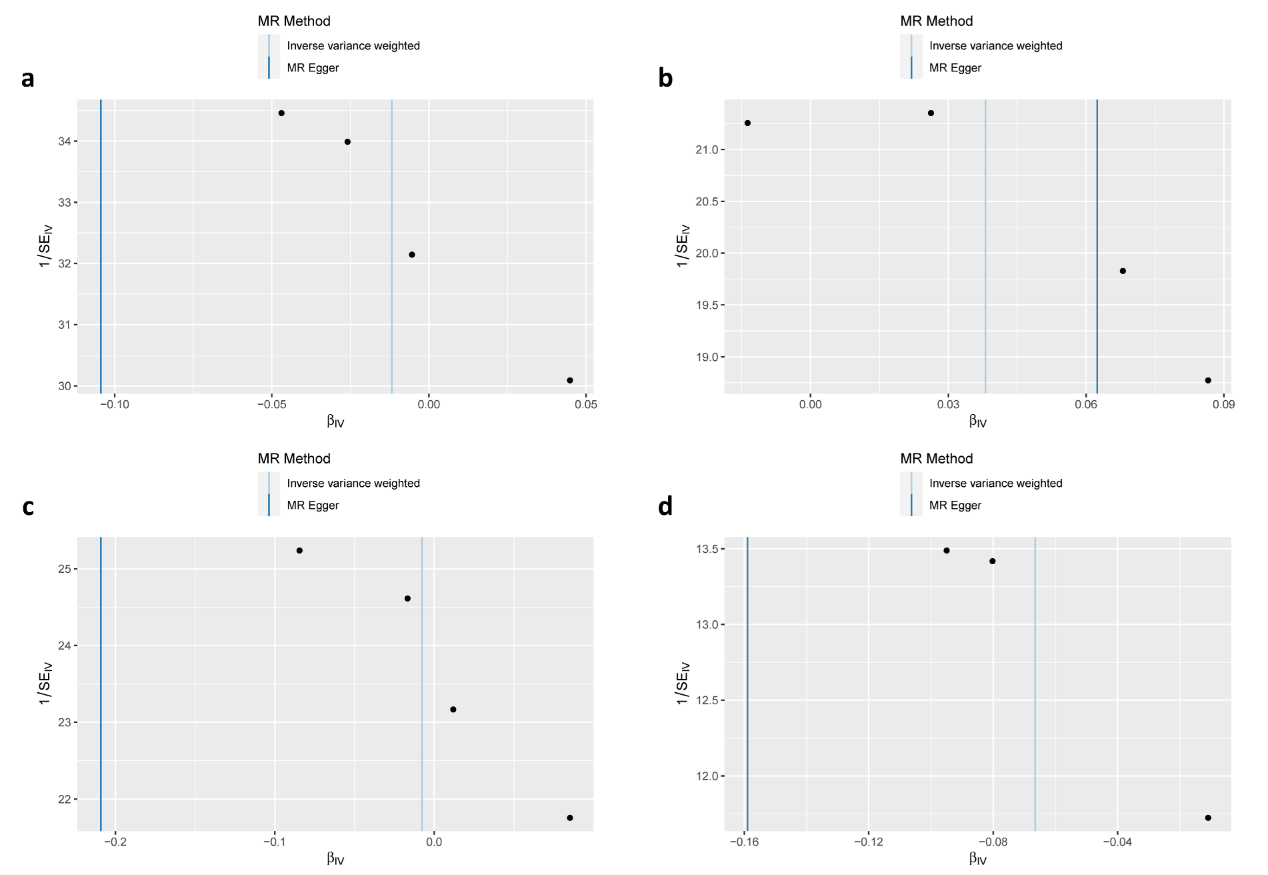


**Figure S4-C.** Funnel plots from genetically predicted agreeableness on the risk of (a) LC; (b) LUSC; (c) LUAD; (d) SCLC.


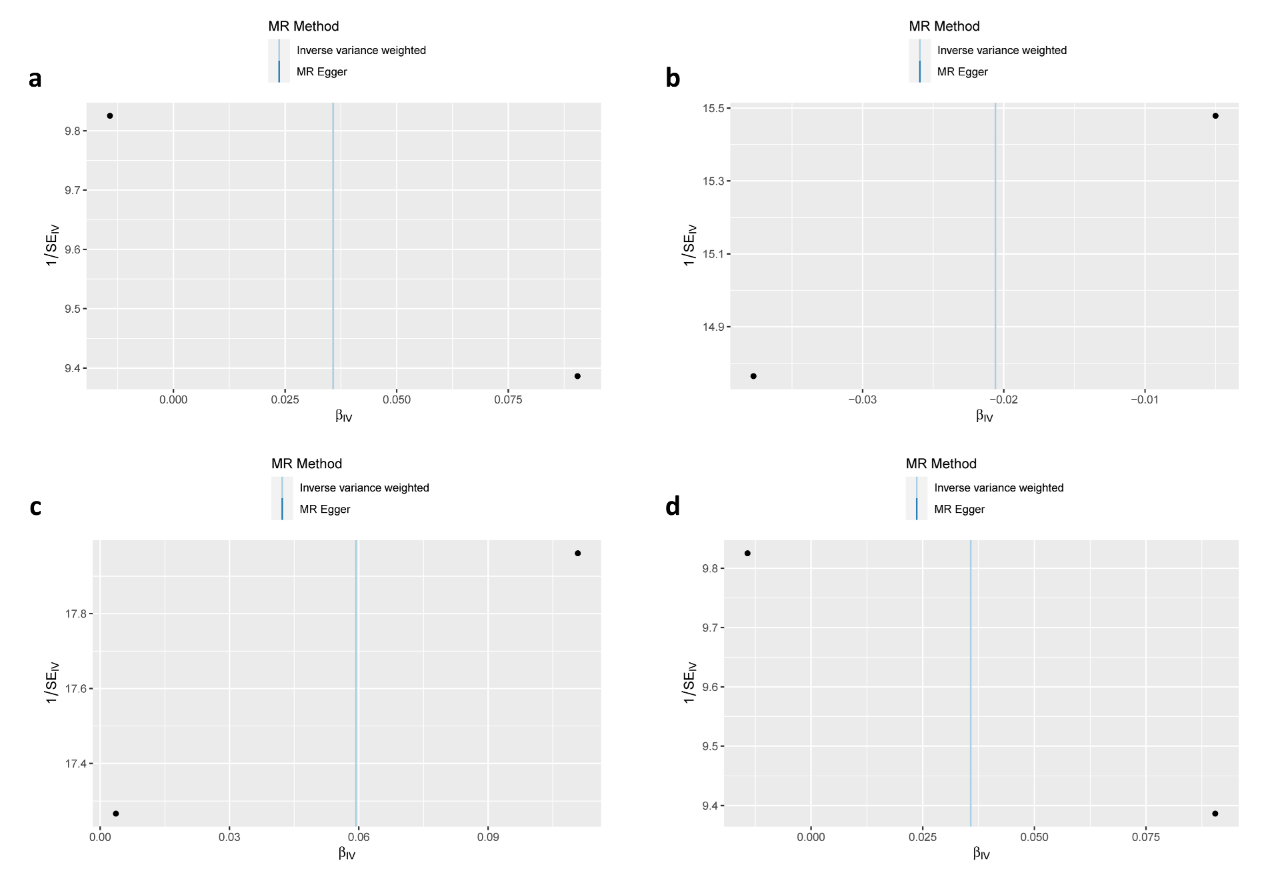


**Figure S4-D.** Funnel plots from genetically predicted conscientiousness on the risk of (a) LC; (b) LUSC; (c) LUAD; (d) SCLC.


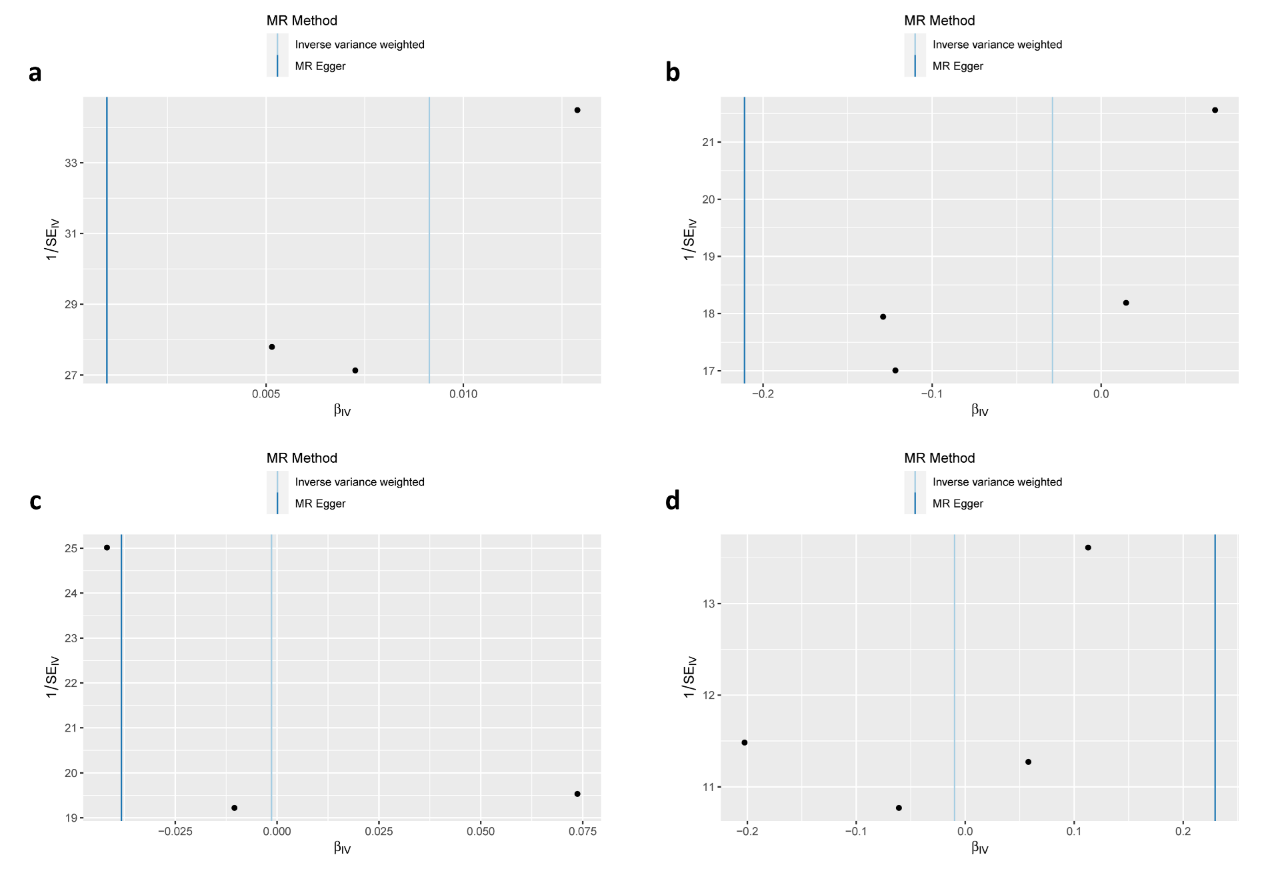


**Figure S4-E.** Funnel plots from genetically predicted openness on the risk of (a) LC; (b) LUSC; (c) LUAD; (d) SCLC.


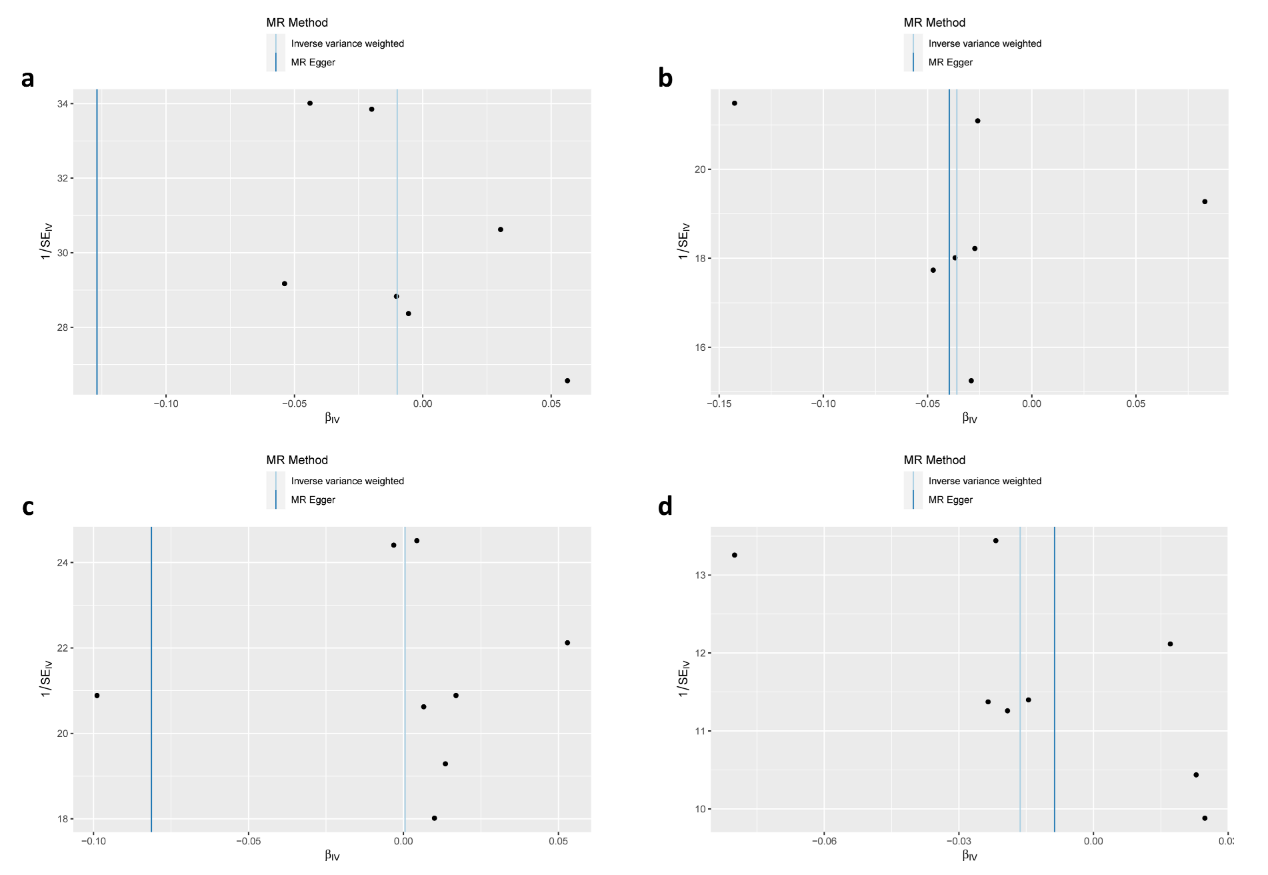


**Figure S4-F.** Funnel plots from genetically predicted schizophrenia on the risk of (a) LC; (b) LUSC; (c) LUAD; (d) SCLC.


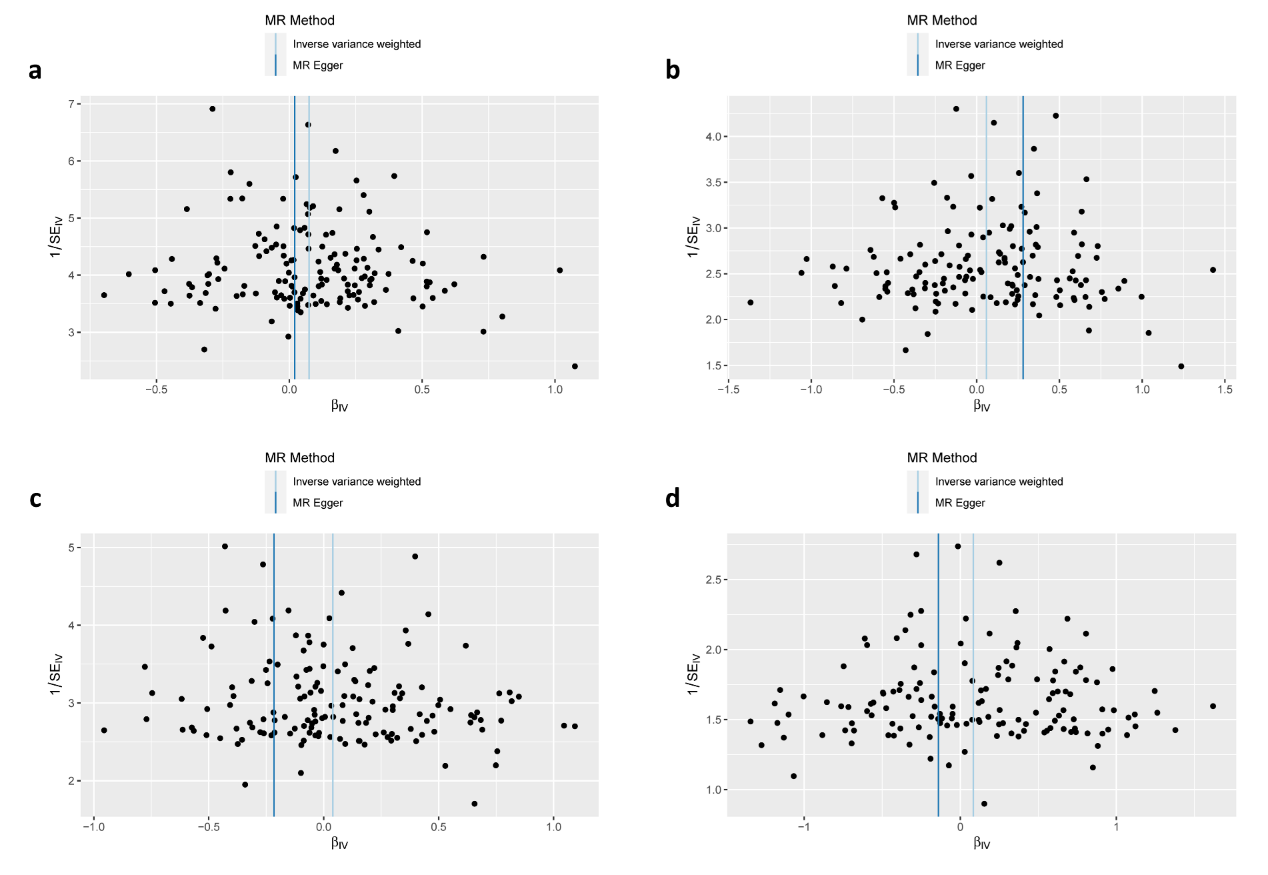


**Figure S4-G.** Funnel plots from genetically predicted ADHD on the risk of (a) LC; (b) LUSC; (c) LUAD; (d) SCLC.


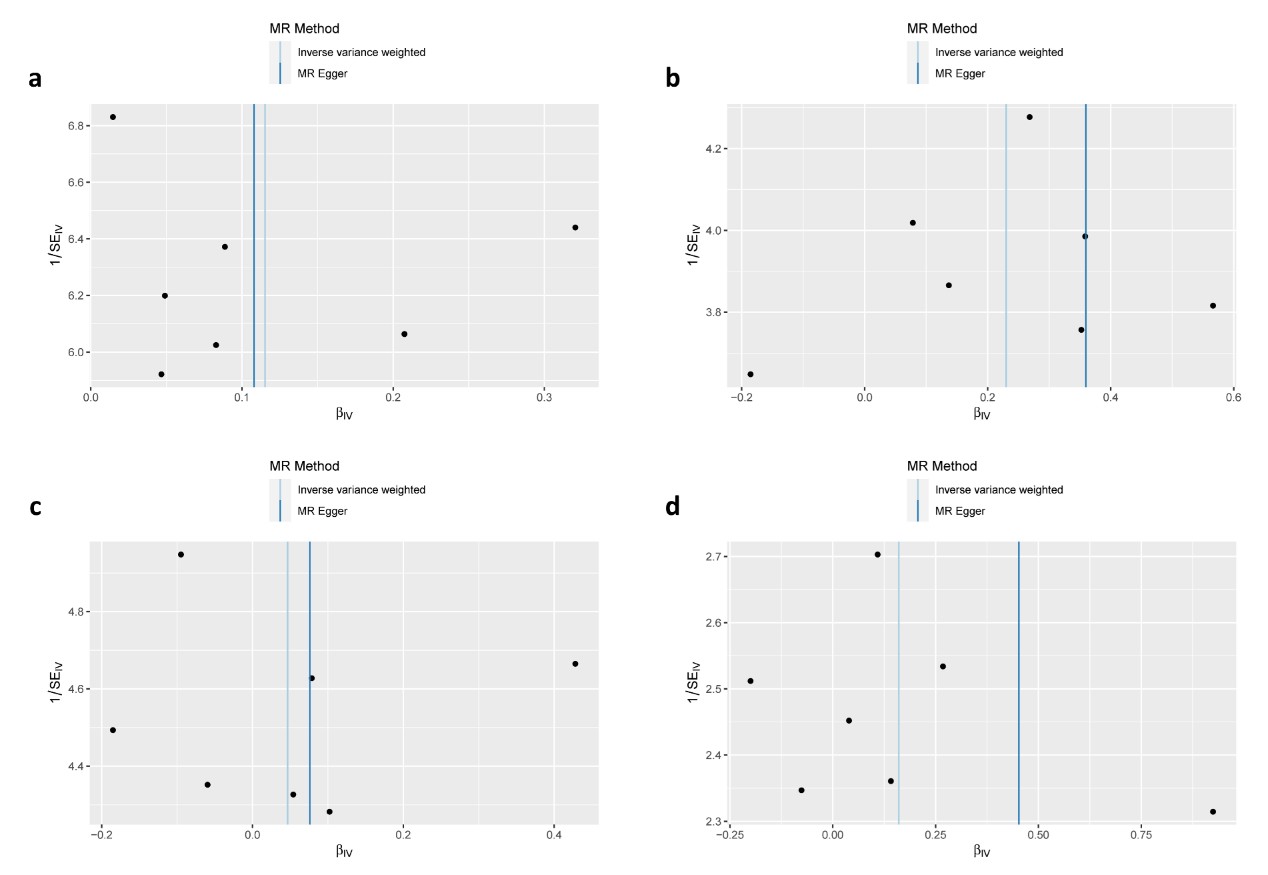


**Figure S4-H.** Funnel plots from genetically predicted MDD on the risk of (a) LC; (b) LUSC; (c) LUAD; (d) SCLC.


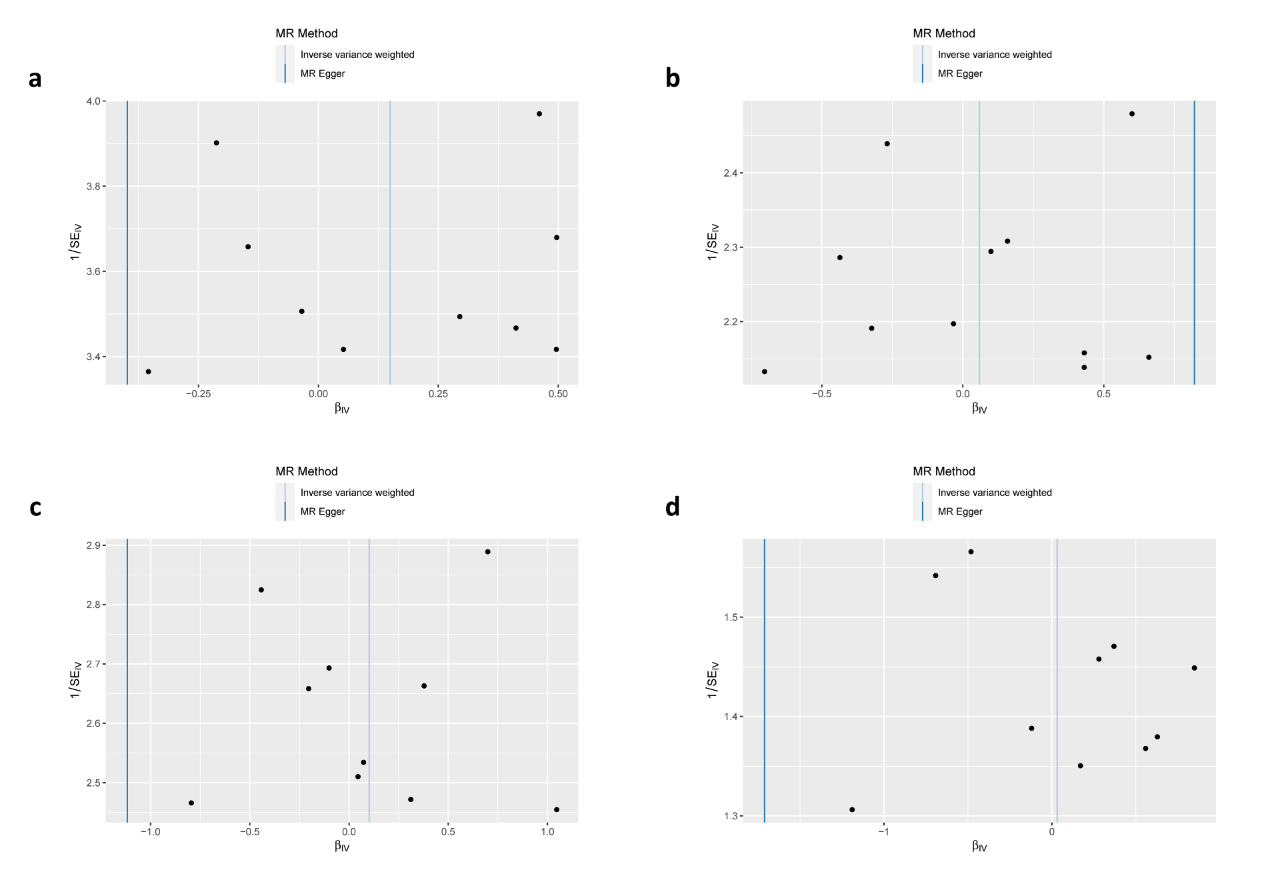


**Figure S4-I.** Funnel plots from genetically predicted ASD on the risk of (a) LC; (b) LUSC; (c) LUAD; (d) SCLC.


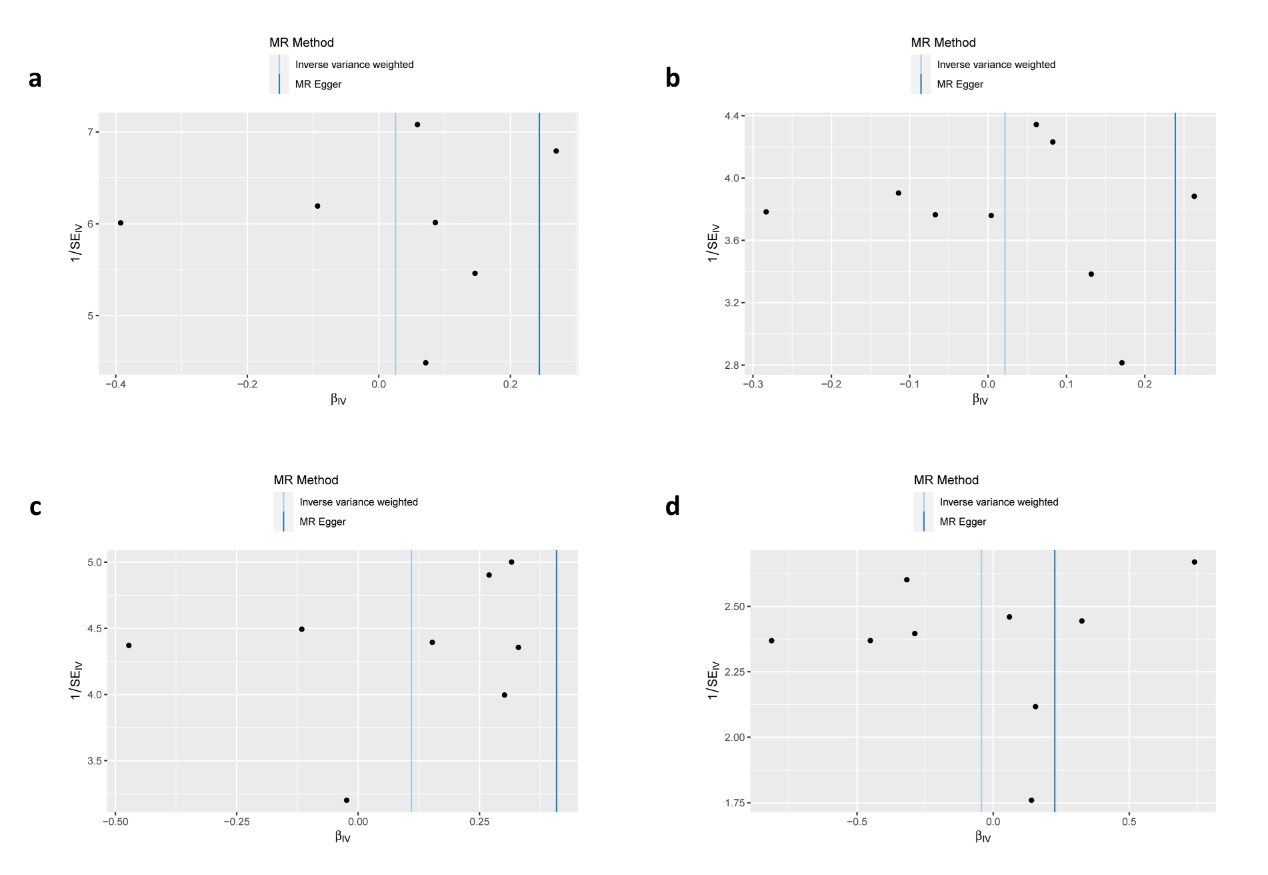


**Figure S4-J.** Funnel plots from genetically predicted BD on the risk of (a) LC; (b) LUSC; (c) LUAD; (d) SCLC.


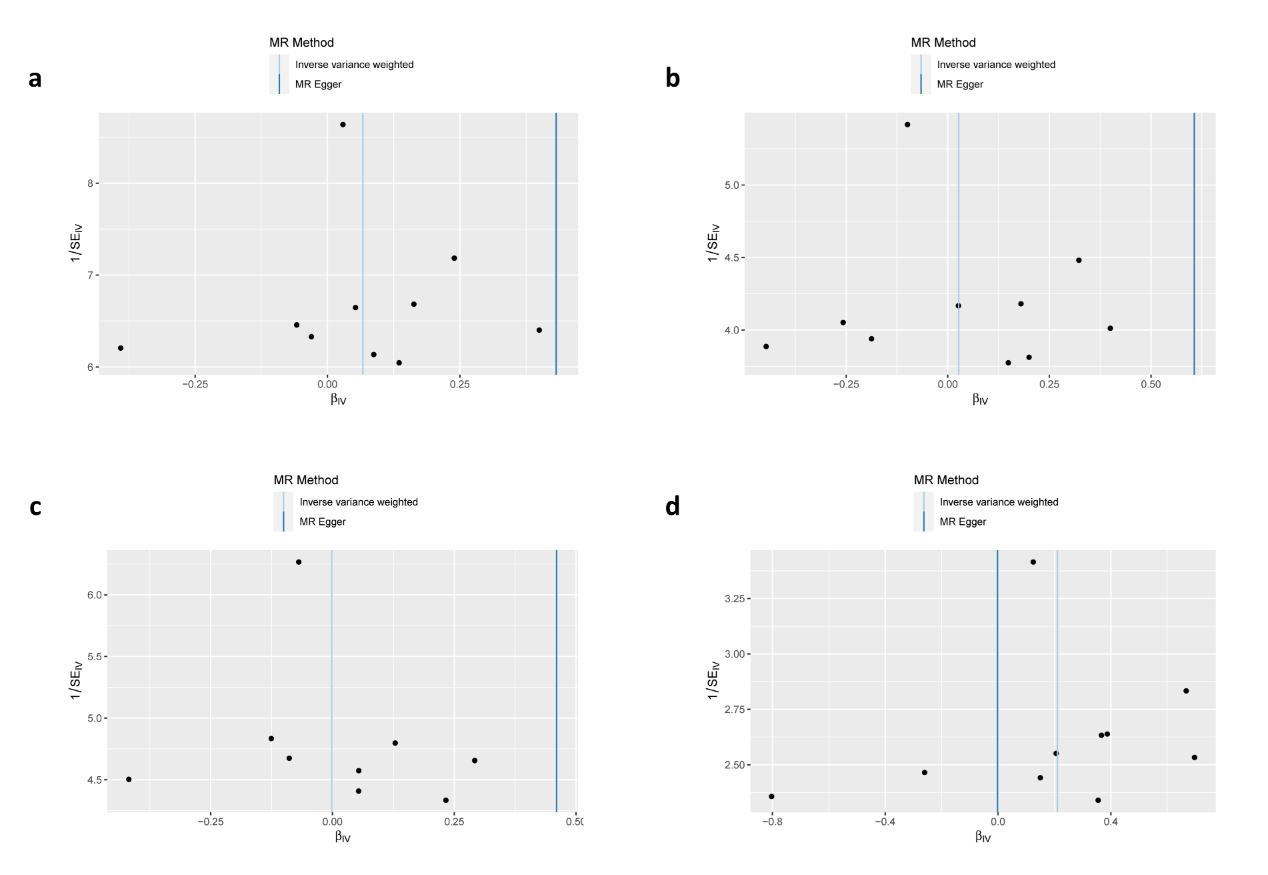


**Figure S4-K.** Funnel plots from genetically predicted insomnia on the risk of (a) LC; (b) LUSC; (c) LUAD; (d) SCLC.


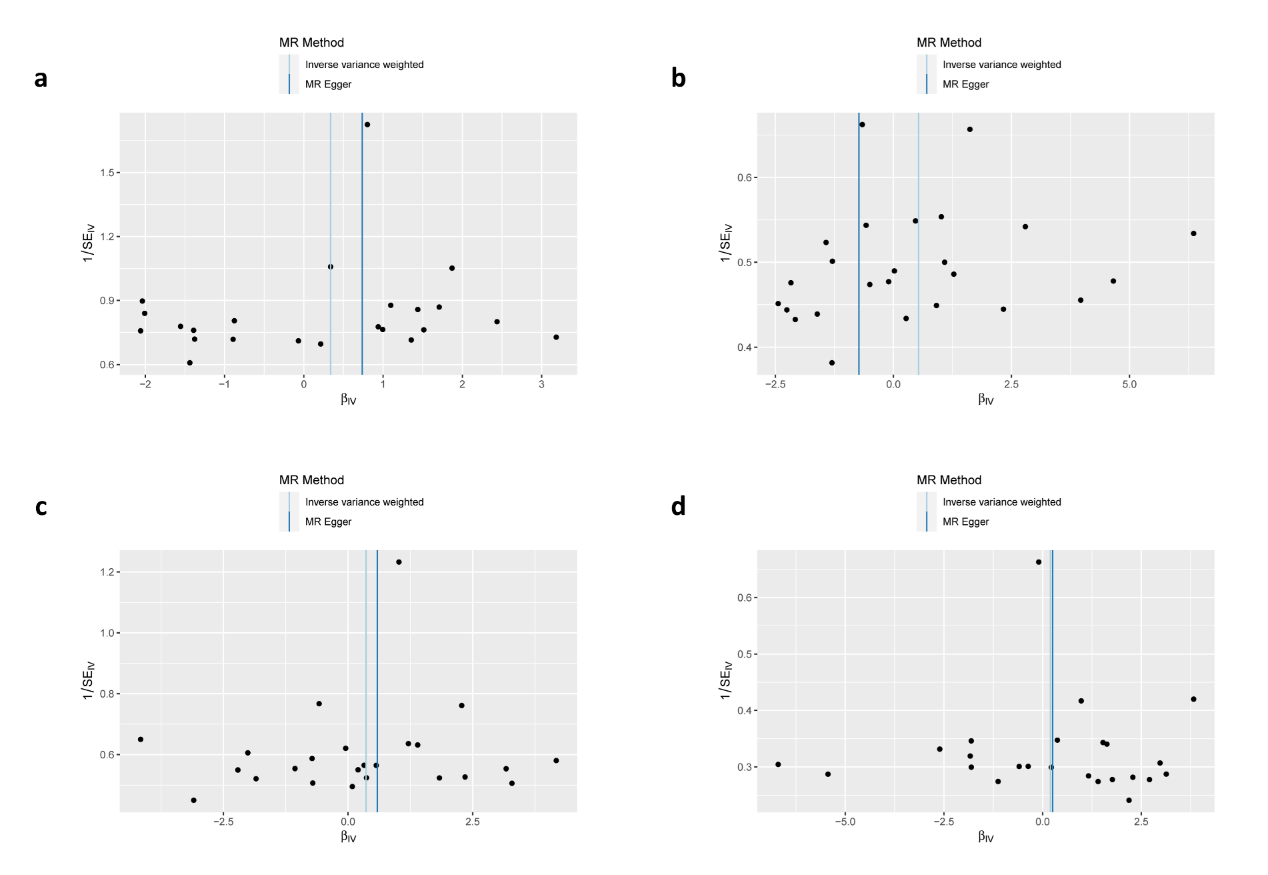


**Figure S4-L.** Funnel plots from genetically predicted anxiety on the risk of (a) LC; (b) LUSC; (c) LUAD; (d) SCLC.


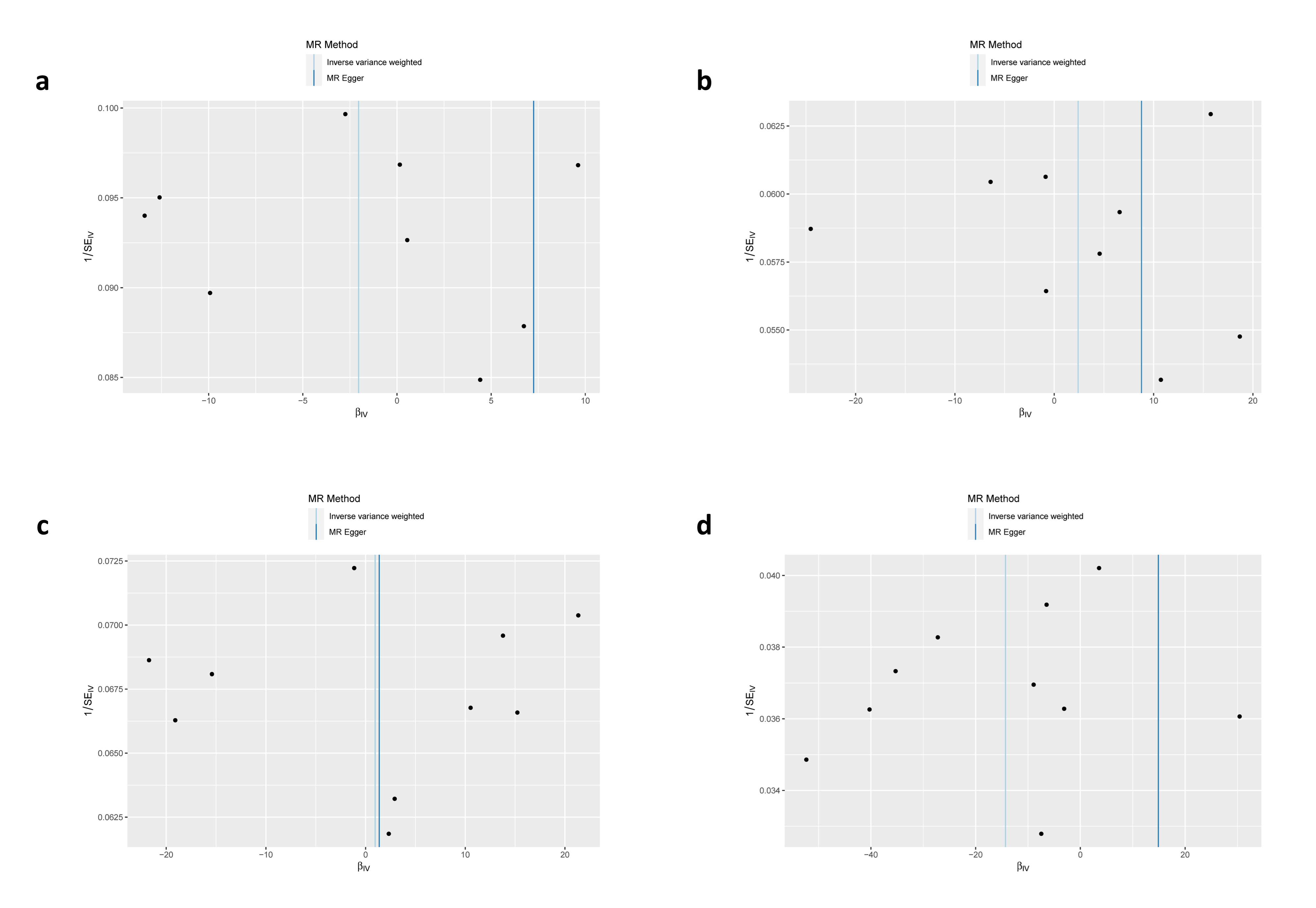


**Figure S5.** Scatter plots of reverse MR analysis from genetically predicted LC/subtypes on personality/psychiatric traits risk.

**Figure S5-A.** Scatter plots from genetically predicted LC on the risk of (a) neuroticism; (b) extraversion; (c) agreeableness; (d) conscientiousness; (e) openness; (f) schizophrenia; (g) ADHD; (h) MDD; (i) ASD; (j) BD; (k) insomnia; (l) anxiety.

**
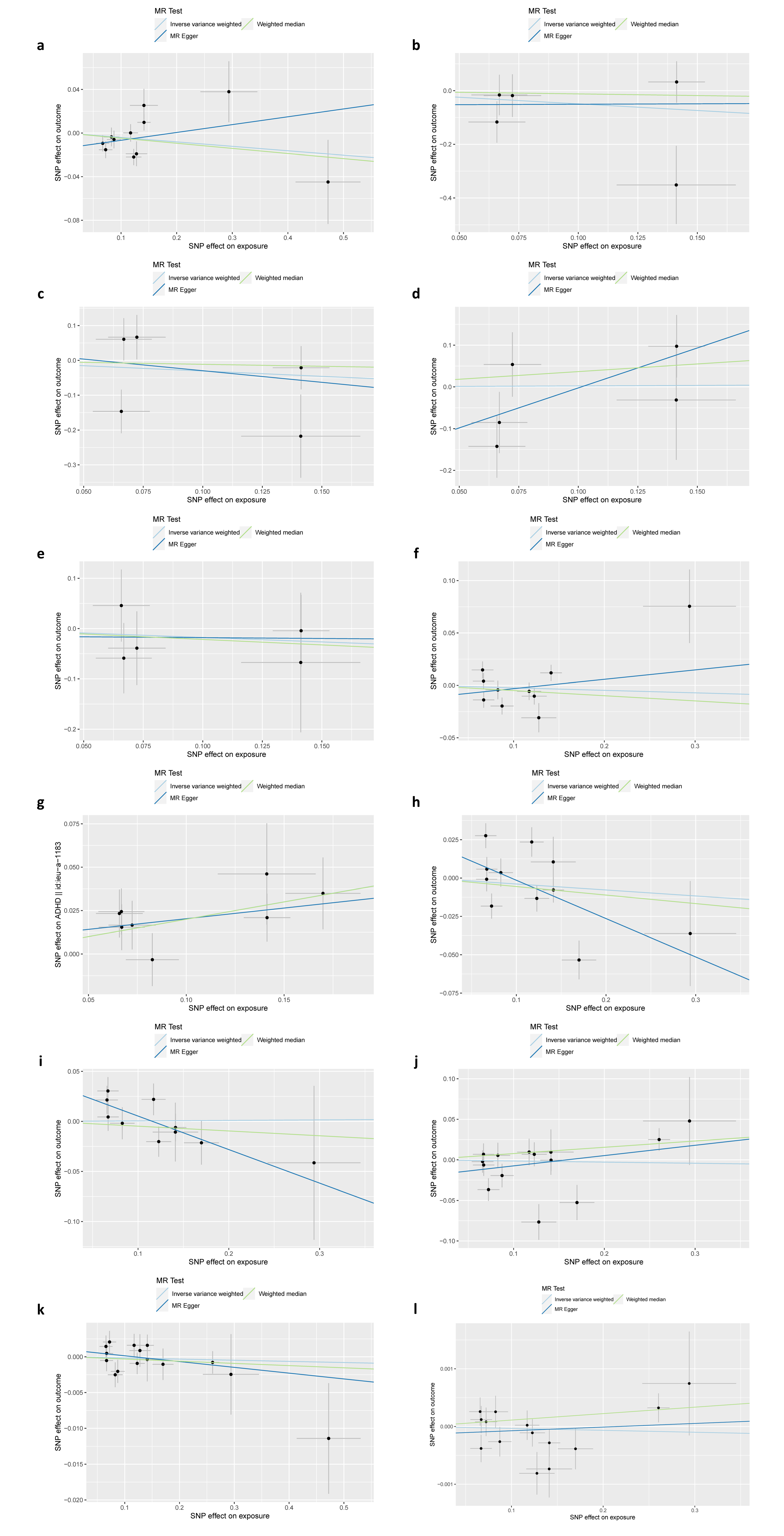
**

**Figure S5-B.** Scatter plots from genetically predicted LUSC on the risk of (a) neuroticism; (b) extraversion; (c) agreeableness; (d) conscientiousness; (e) openness; (f) schizophrenia; (g) ADHD; (h) MDD; (i) ASD; (j) BD; (k) insomnia; (l) anxiety.


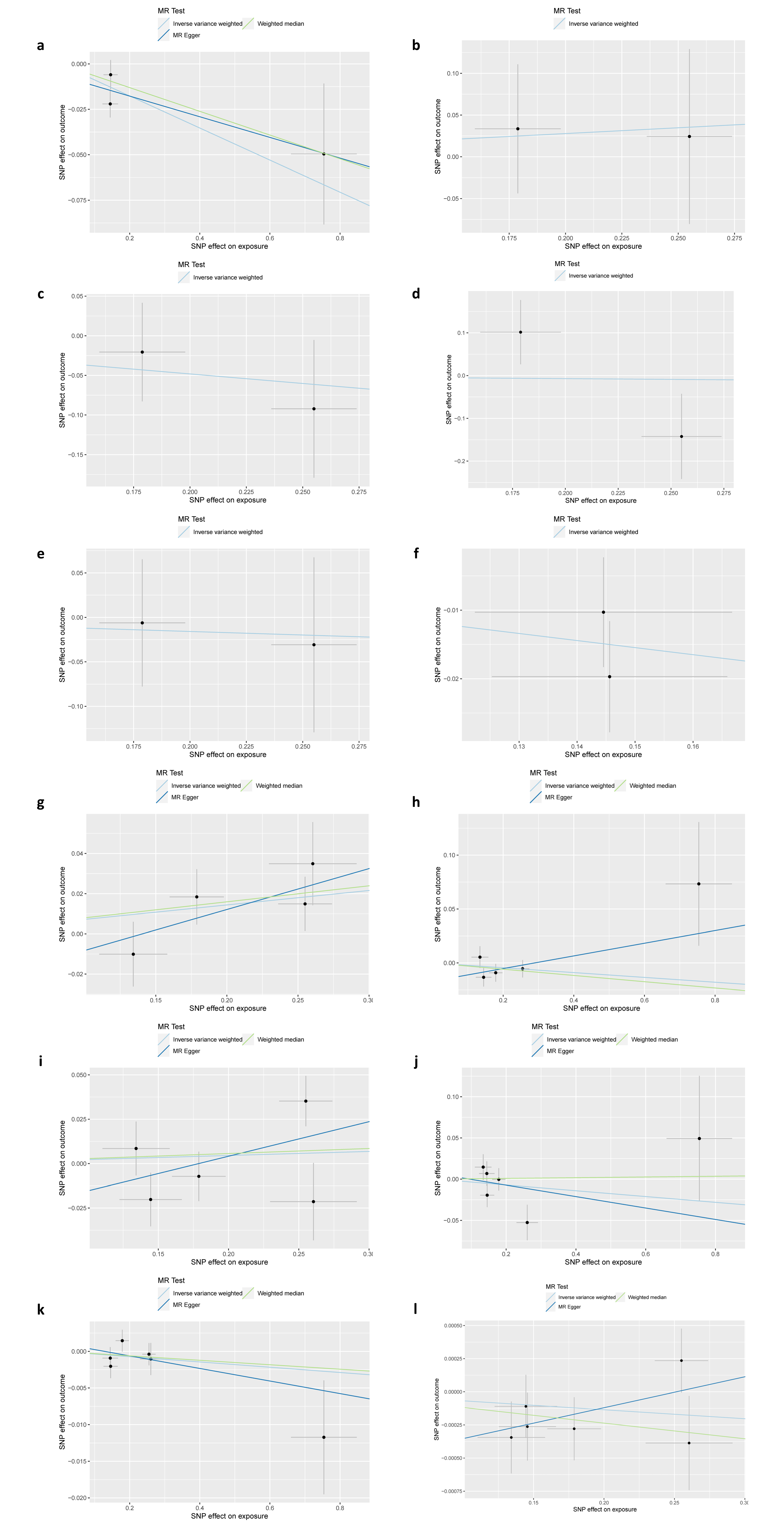


**Figure S5-C.** Scatter plots from genetically predicted LUAD on the risk of (a) neuroticism; (b) extraversion; (c) agreeableness; (d) conscientiousness; (e) openness; (f) schizophrenia; (g) ADHD; (h) MDD; (i) ASD; (j) BD; (k) insomnia; (l) anxiety.


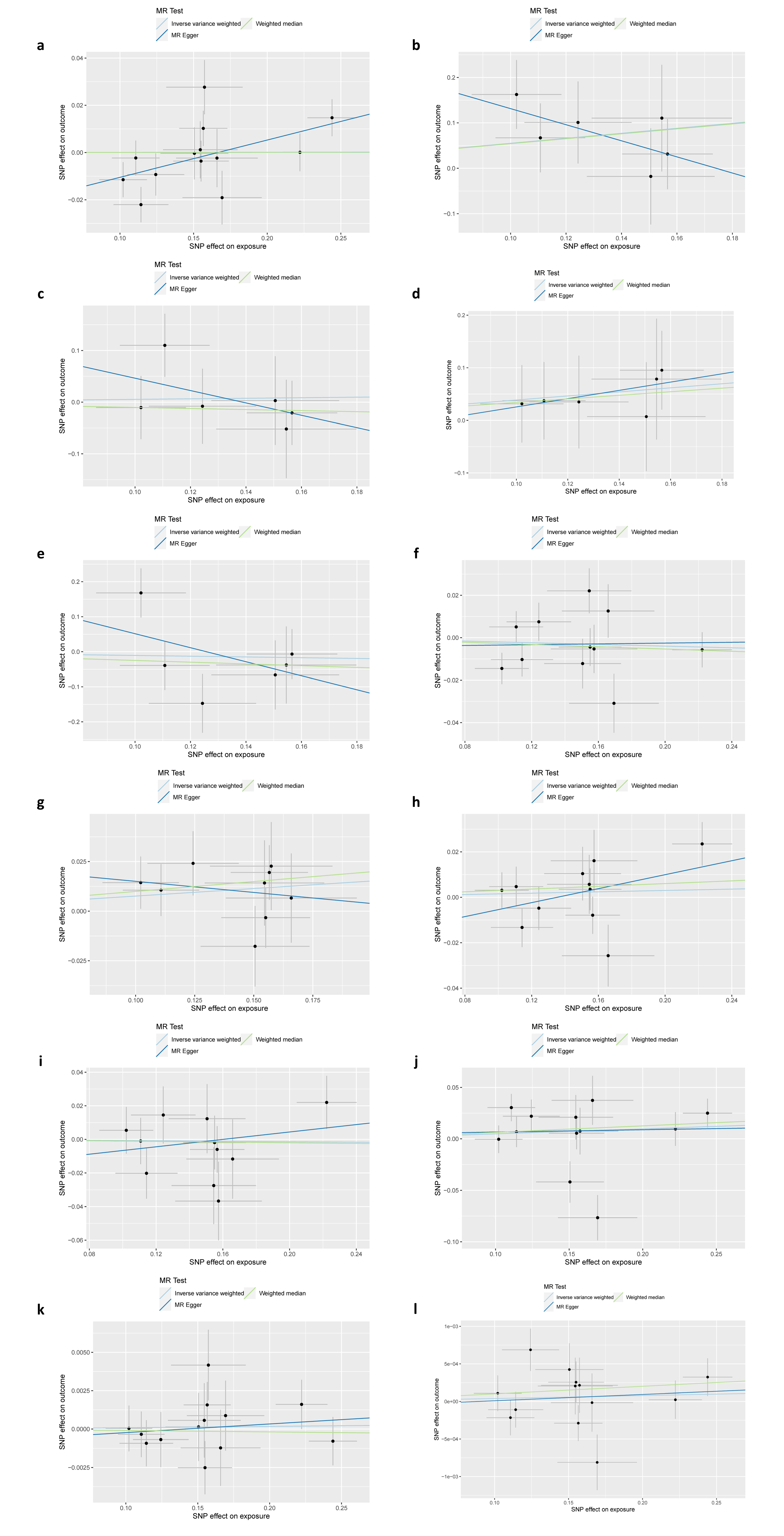


**Figure S5-D.** Scatter plots from genetically predicted SCLC on the risk of (a) neuroticism; (b) insomnia.


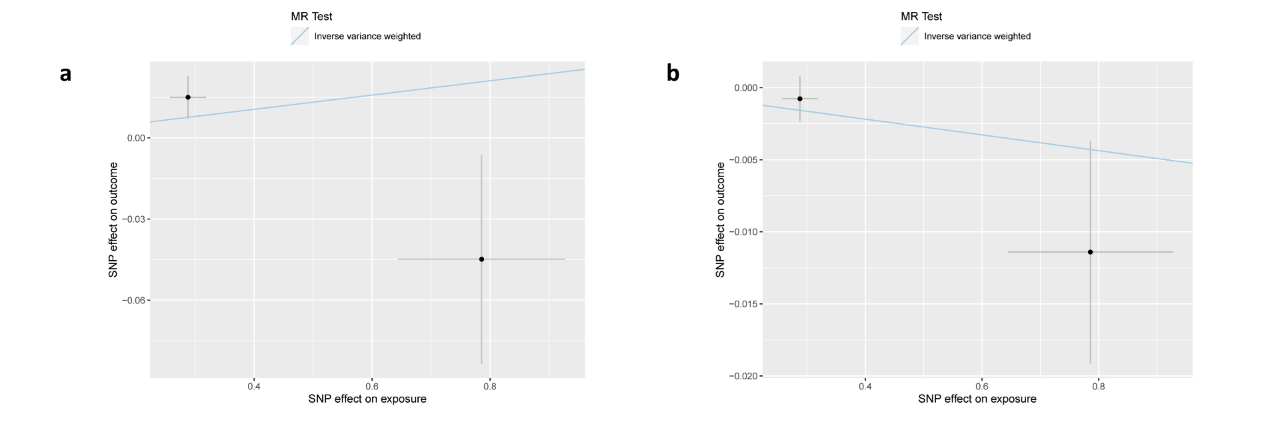


**Figure S6.** Leave-one-out plots of reverse MR analysis from genetically predicted LC/subtypes on personality/psychiatric traits risk.

**Figure S6-A.** Leave-one-out plots from genetically predicted LC on the risk of (a) neuroticism; (b) extraversion; (c) agreeableness; (d) conscientiousness; (e) openness; (f) schizophrenia; (g) ADHD; (h) MDD; (i) ASD; (j) BD; (k) insomnia; (l) anxiety.


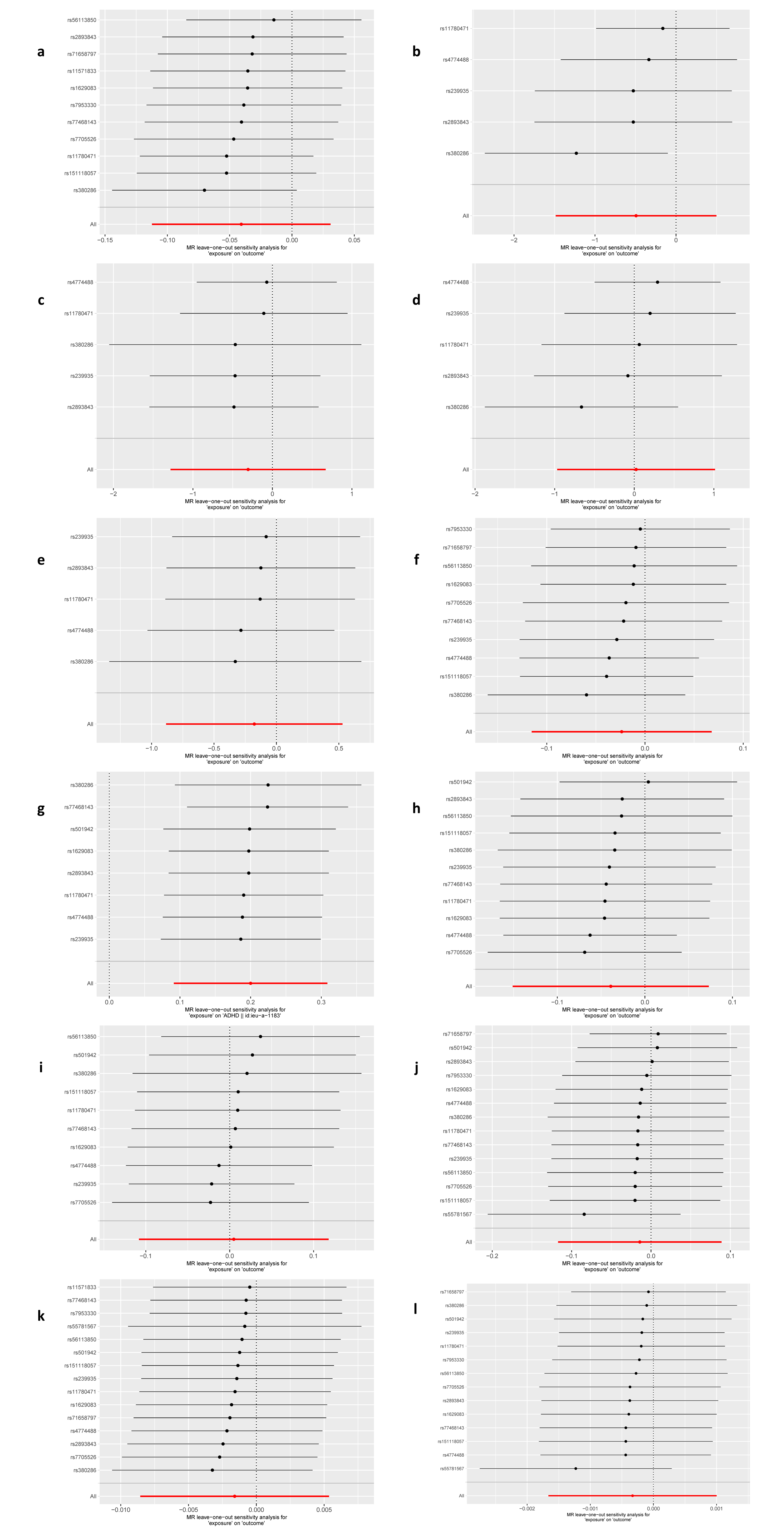


**Figure S6-B.** Leave-one-out plots from genetically predicted LUSC on the risk of (a) neuroticism; (b) ADHD; (c) MDD; (d) ASD; (e) BD; (f) insomnia; (g) anxiety.


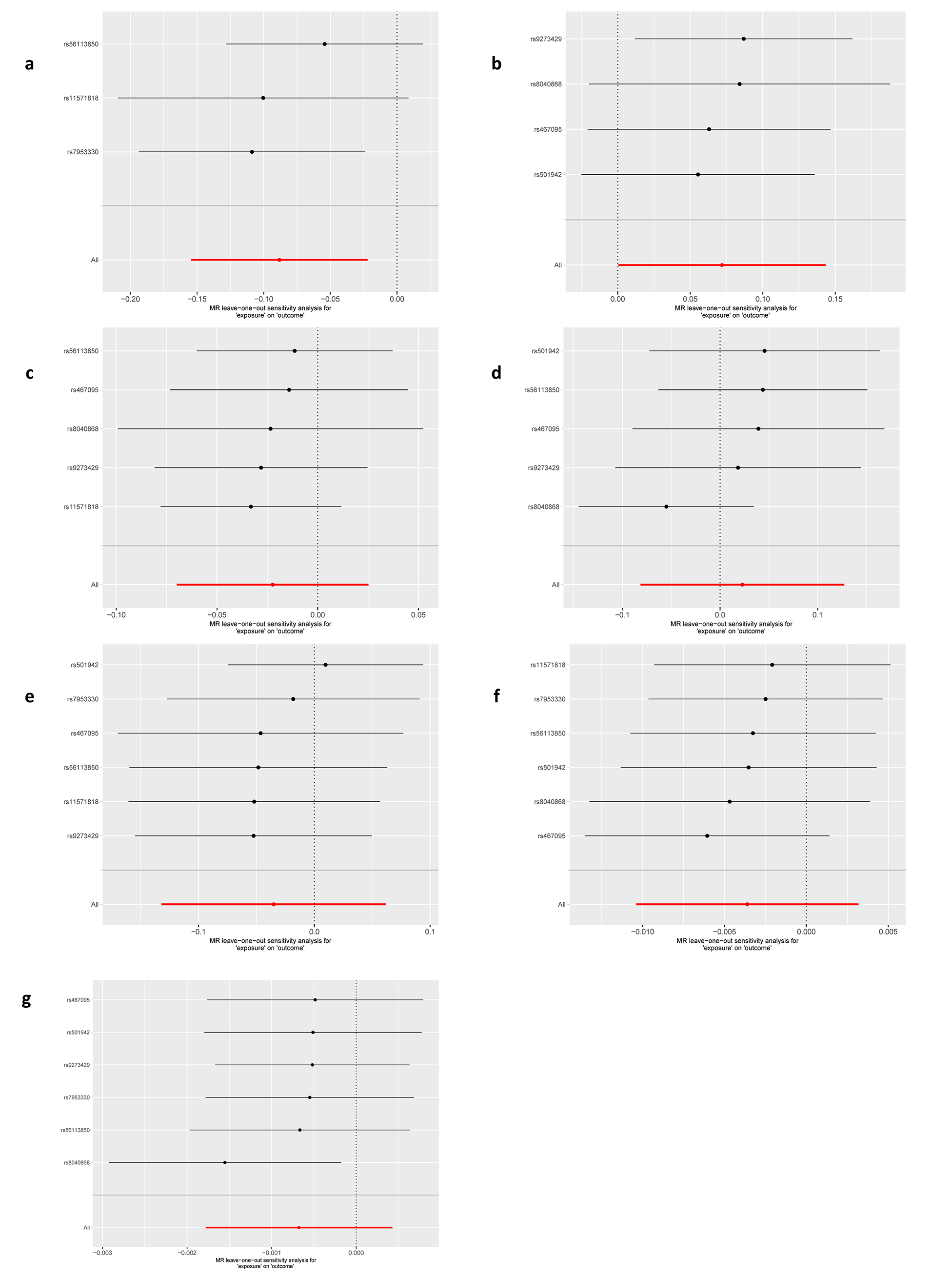


**Figure S6-C.** Leave-one-out plots from genetically predicted LUAD on the risk of (a) neuroticism; (b) extraversion; (c) agreeableness; (d) conscientiousness; (e) openness; (f) schizophrenia; (g) ADHD; (h) MDD; (i) ASD; (j) BD; (k) insomnia; (l) anxiety.


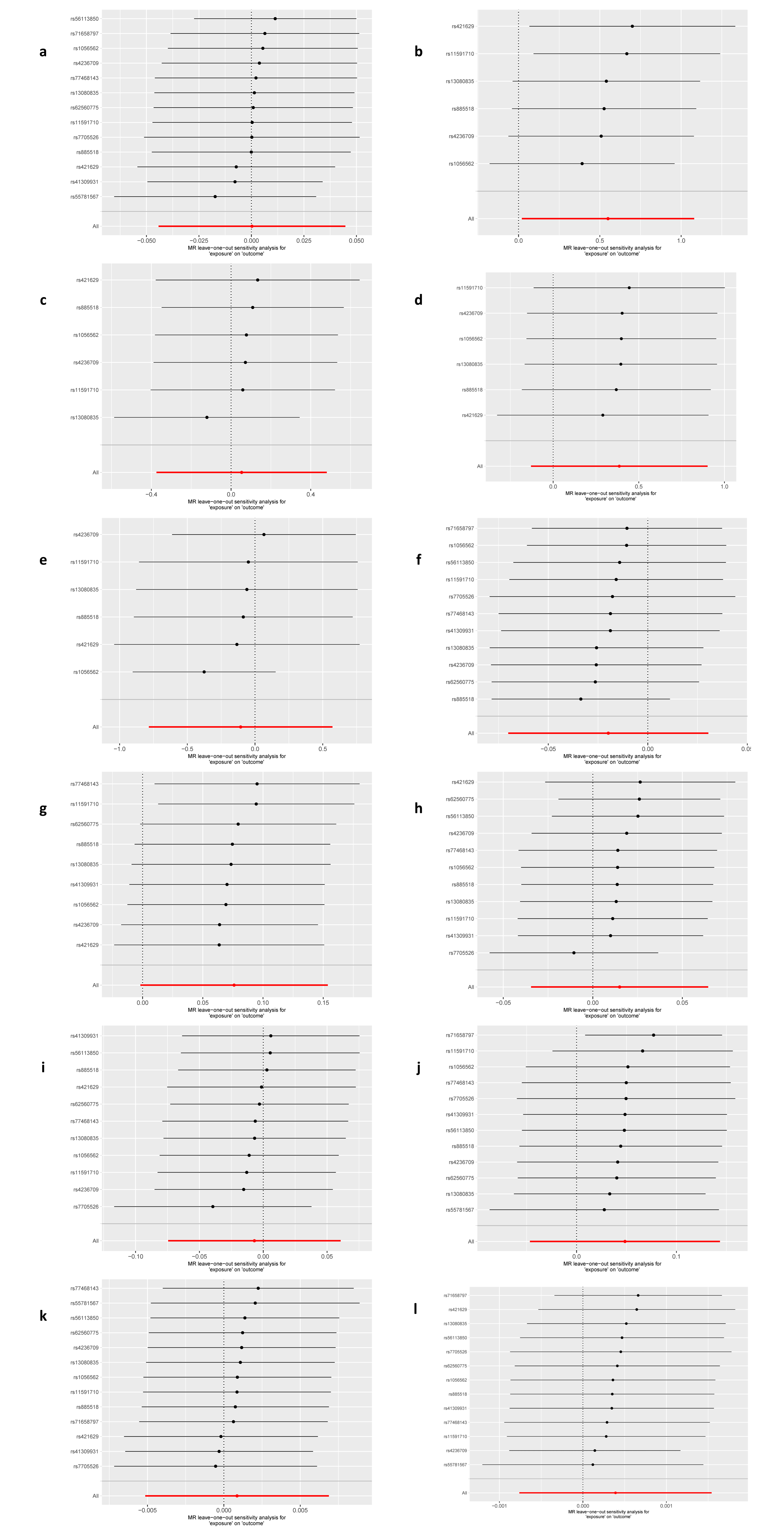


**Figure S7.** Funnel plots of reverse MR analysis from genetically predicted LC/subtypes on personality/psychiatric traits risk.

**Figure S7-A.** Funnel plots from genetically predicted LC on the risk of (a) neuroticism; (b) extraversion; (c) agreeableness; (d) conscientiousness; (e) openness; (f) schizophrenia; (g) ADHD; (h) MDD; (i) ASD; (j) BD; (k) insomnia; (l) anxiety.


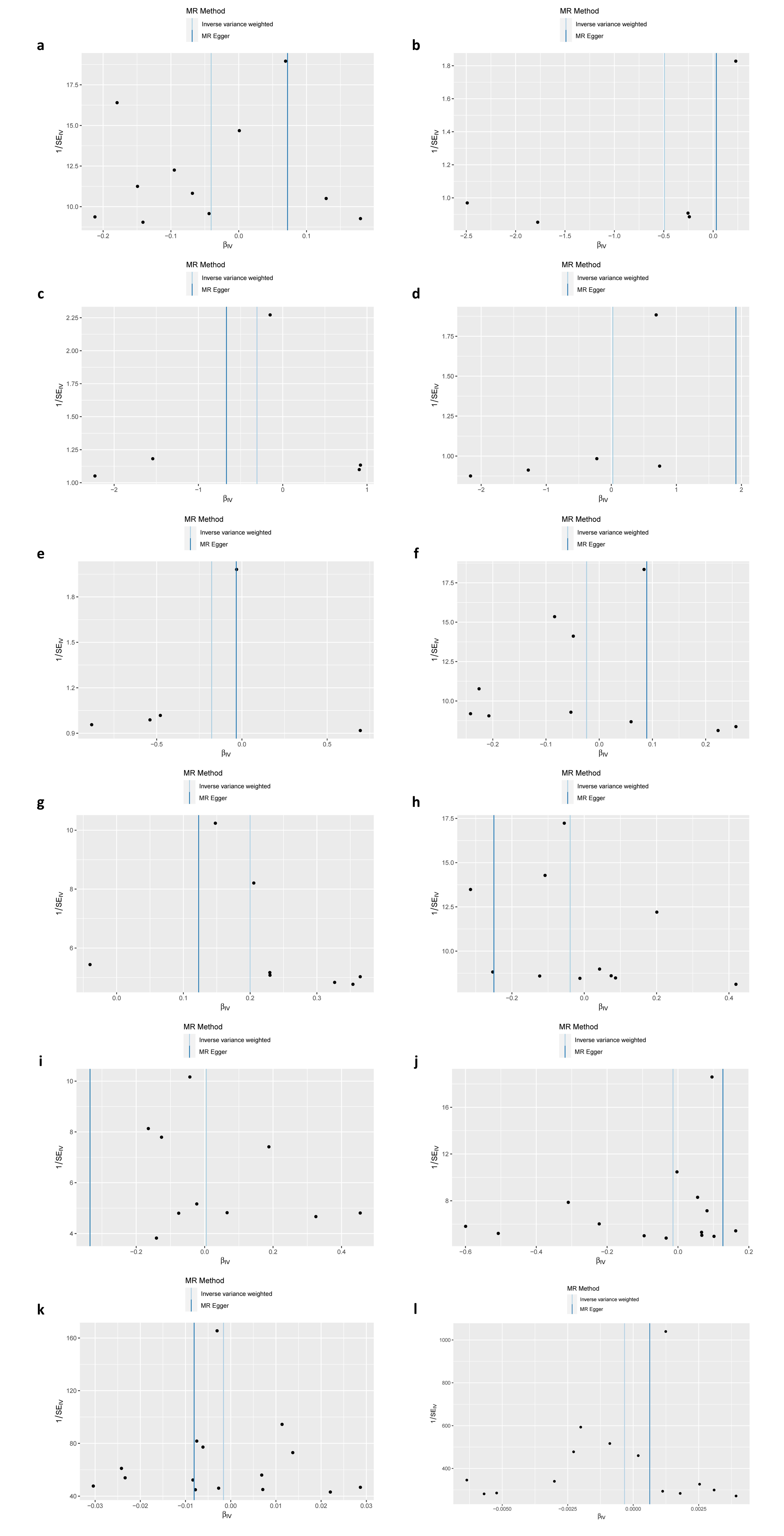


**Figure S7-B.** Funnel plots from genetically predicted LUSC on the risk of (a) neuroticism; (b) extraversion; (c) agreeableness; (d) conscientiousness; (e) openness; (f) schizophrenia; (g) ADHD; (h) MDD; (i) ASD; (j) BD; (k) insomnia; (l) anxiety.


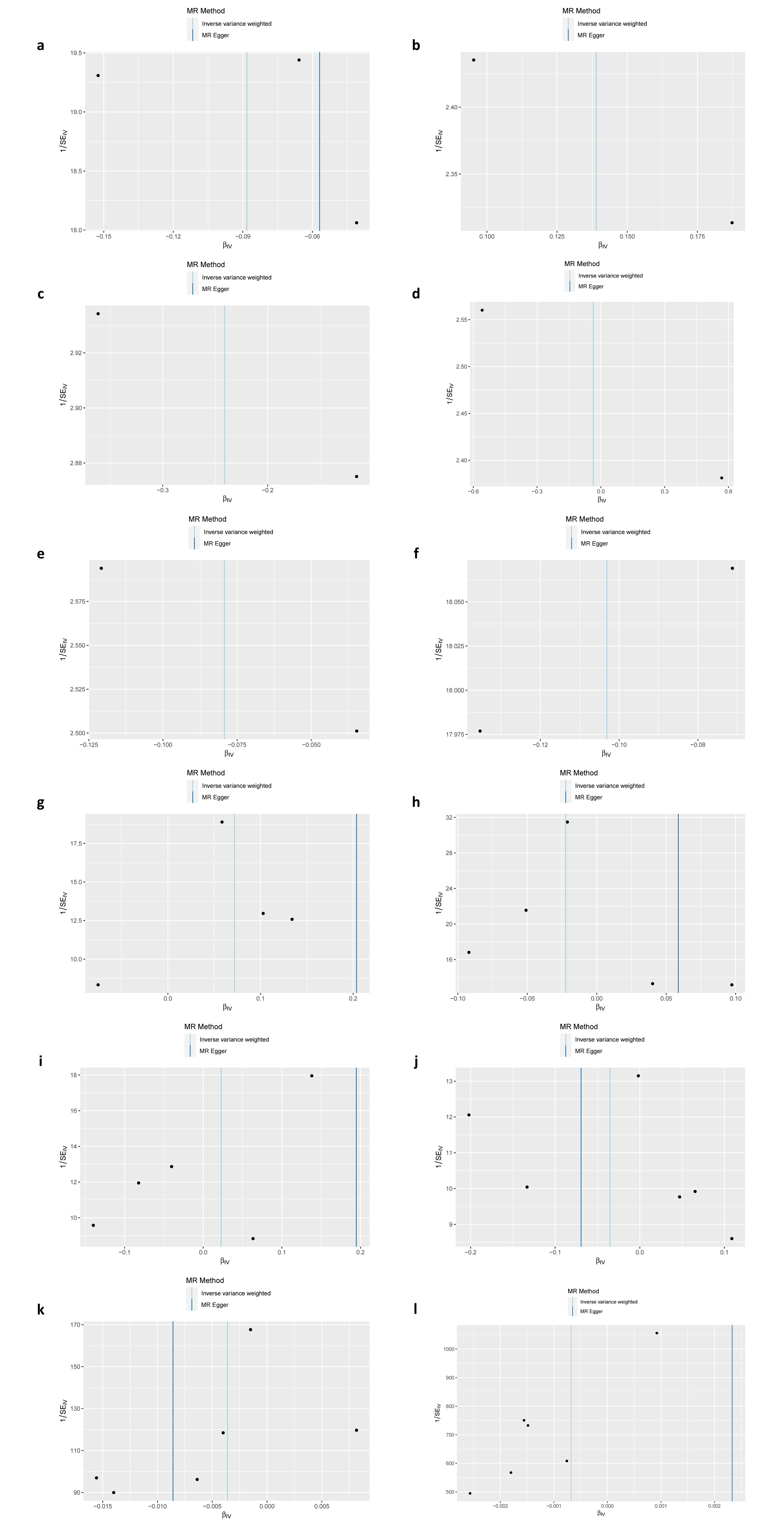


**Figure S7-C.** Funnel plots from genetically predicted LUAD on the risk of (a) neuroticism; (b) extraversion; (c) agreeableness; (d) conscientiousness; (e) openness; (f) schizophrenia; (g) ADHD; (h) MDD; (i) ASD; (j) BD; (k) insomnia; (l) anxiety.


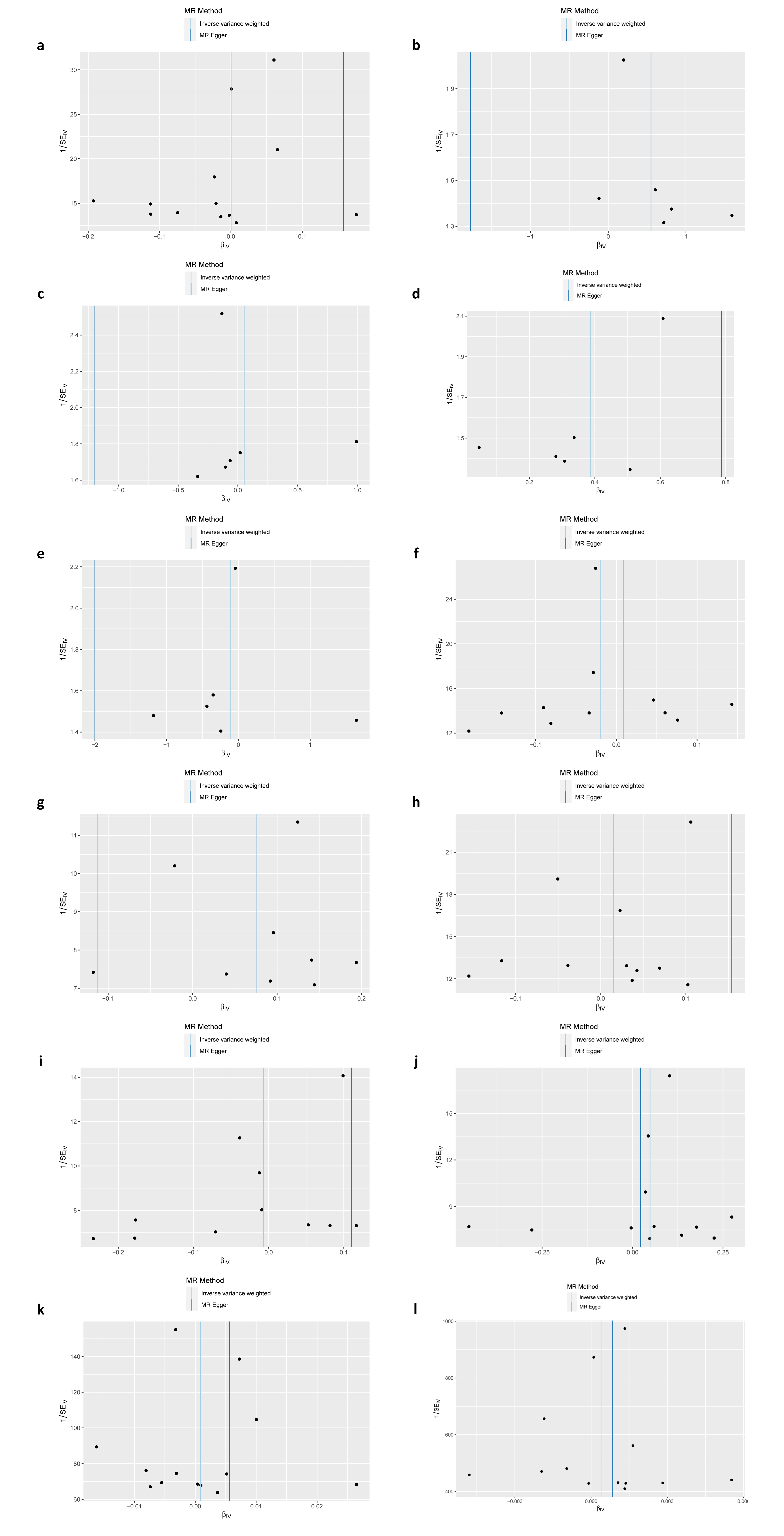


**Figure S7-D.** Funnel plots from genetically predicted SCLC on the risk of (a) neuroticism; (b) insomnia.


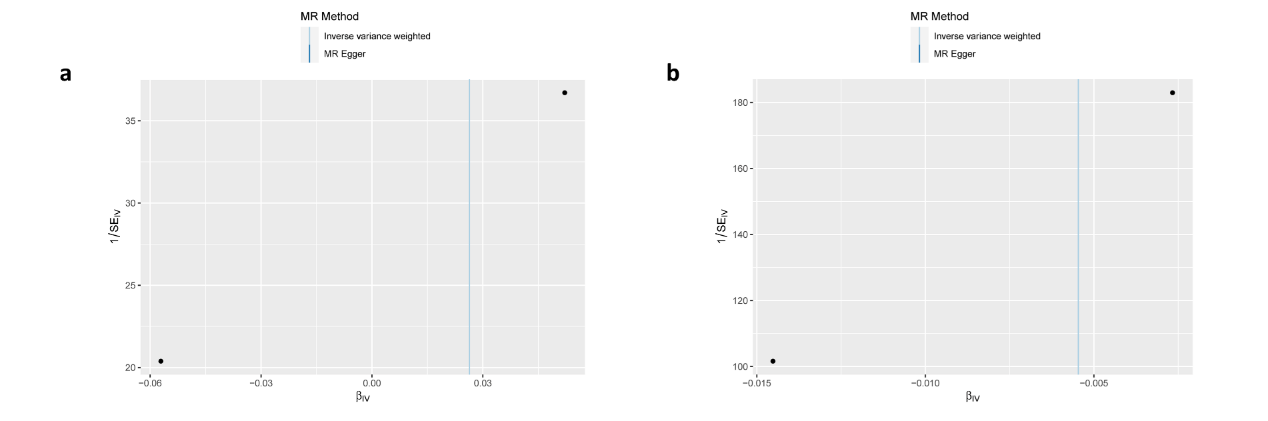


**Figure S8.** Supplementary science mapping using Scopus and PubMed databases**.**

**Figure S8-A:** Overlay network of keyword co-occurrence (a) Scopus; (b) PubMed. Each node represents a keyword. The node and font sizes are proportional to the frequency of keywords and the line thickness reflects the co-occurrence frequency. Purple nodes represent the early focus on the topic, whereas yellow nodes indicate current attention.


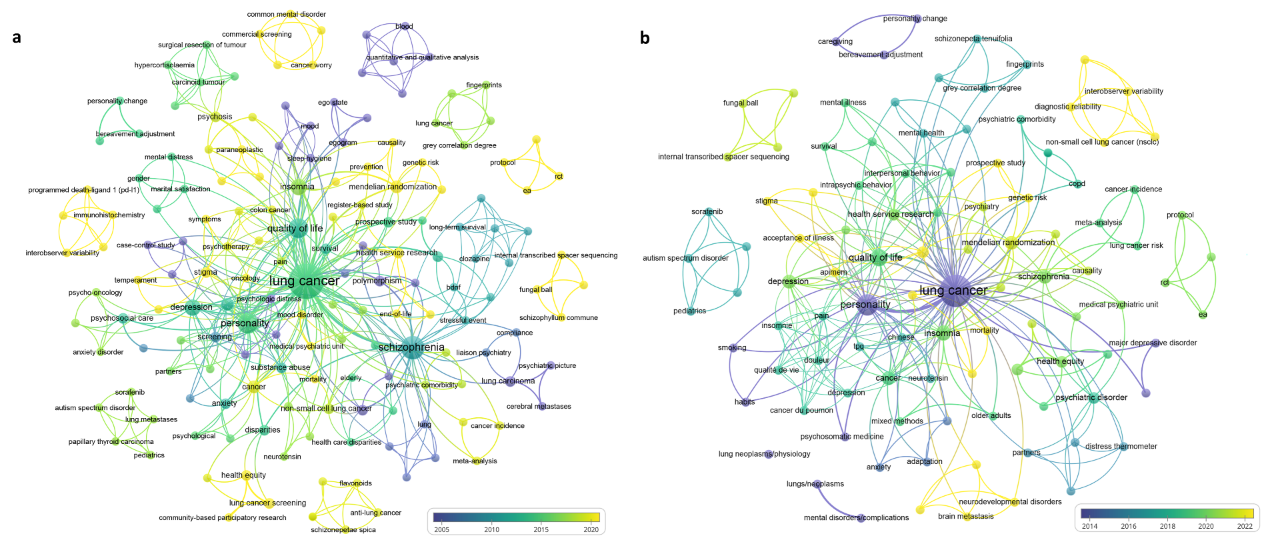


**Figure S8-B.** The plot of the articles’ growth trend (a) Scopus; (b) PubMed.


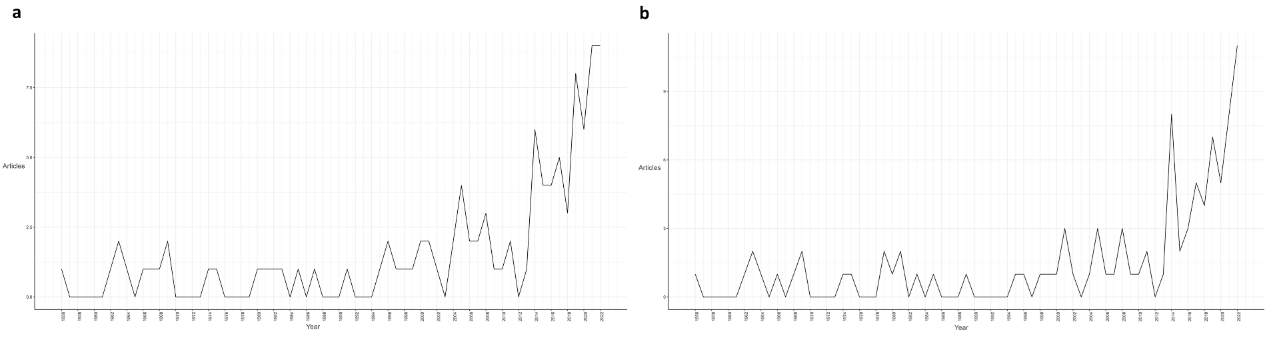


**Figure S8-C.** Overlay visualization network map of the major articles (Scopus). Each node means an article. The size of circles and fonts is proportional to TC. The more purple the node, the earlier the year of publication, and the yellower the node, the more recent the publication date. The node name shows the first author and the publication year of a paper.


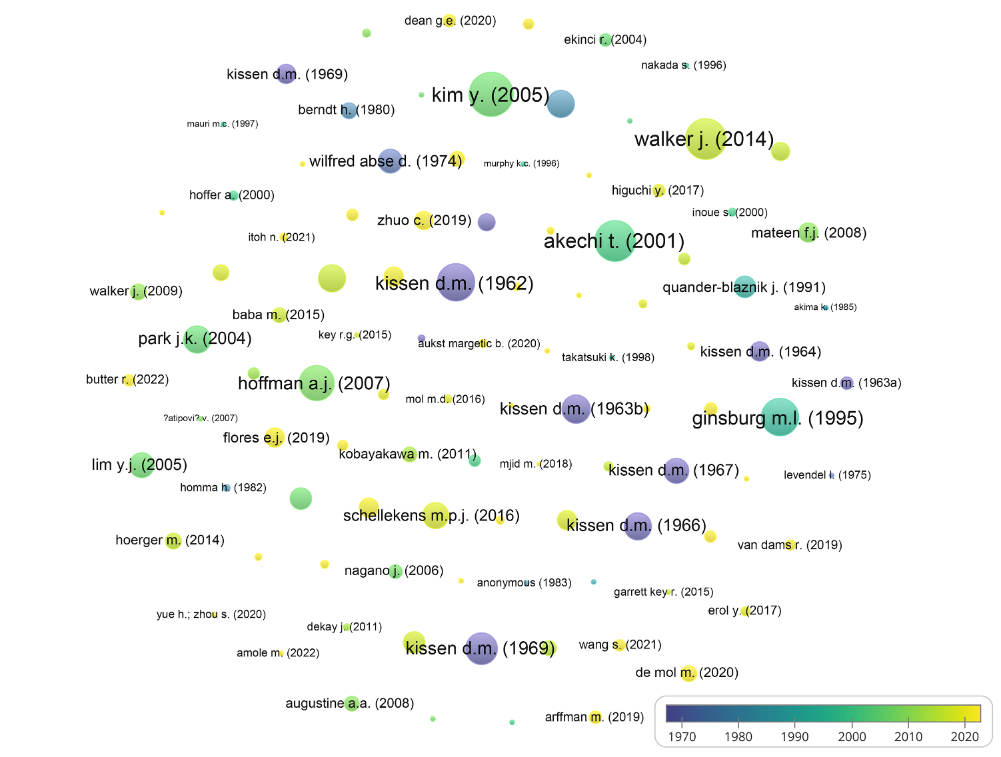


**Figure S8-D.** Country collaboration network map (Scopus). Each node stands for a country. The size of circles and fonts reflects the number of papers in certain countries/regions. The thickness of the connection line between countries/regions signifies the frequency of collaborations. The distance between the two circles demonstrates the relatedness of their link.

**
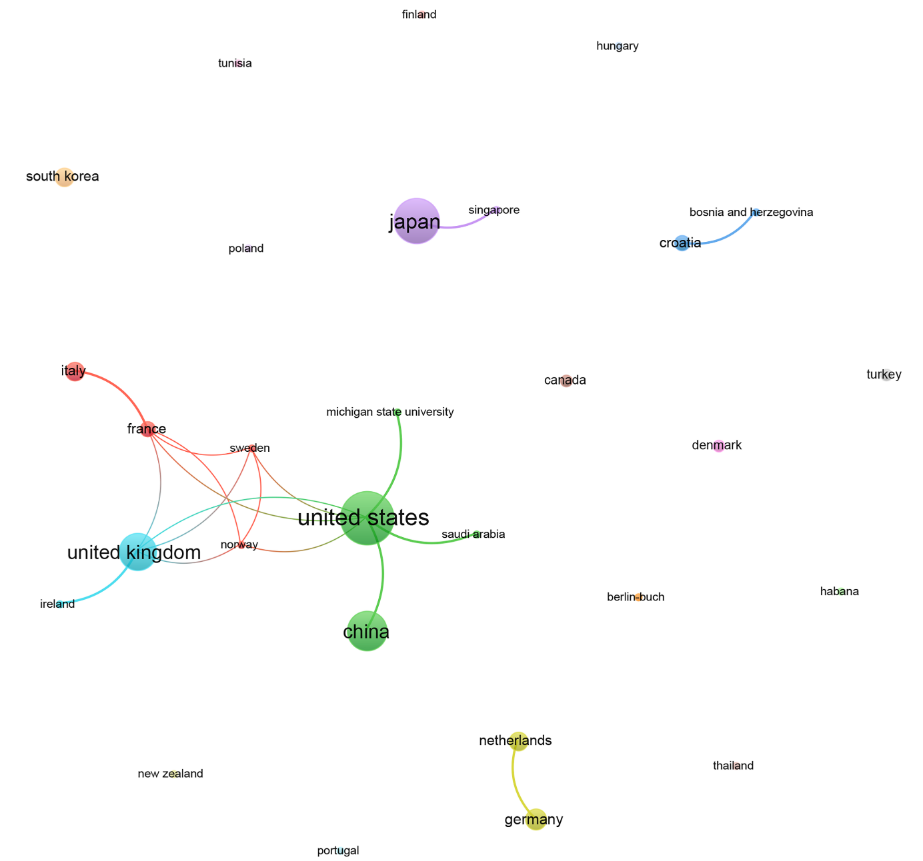
**

**Figure S8-E.** Institution collaboration network map (a) Scopus; (b) PubMed. Each node represents an institution. The larger the size of the circles and fonts, the more papers the institution is involved in. The thickness of the connection line between institutions signifies the frequency of collaborations. Several clusters indicate different institution collaborative networks.

Note: Scopus and PubMed data on institutions may not have been harmonized. Institution names may not have a consistent format. The completeness and reliability were inferior to those of the WoSCC.

**
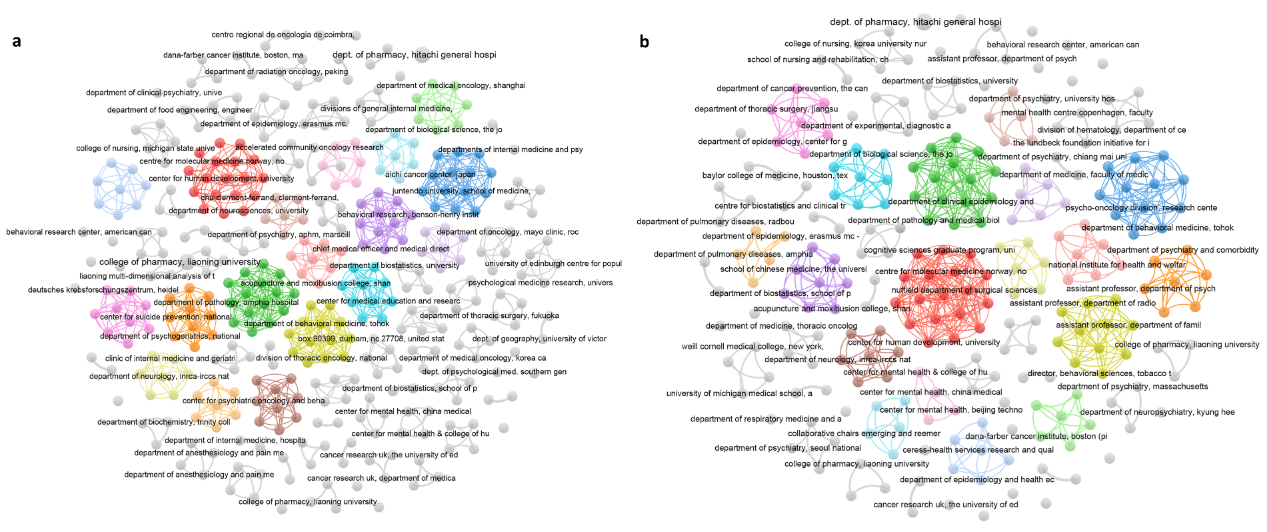
**

**Figure S8-F.** Author collaboration network map (a) Scopus; (b) PubMed. Each node represents authors. The size of circles and fonts demonstrates the number of papers of an author. The thickness of the connecting line between authors shows the frequency of collaborations. Several clusters indicate different author collaborative networks.

Note: The completeness and reliability of the data in Scopus were relatively poor.

**
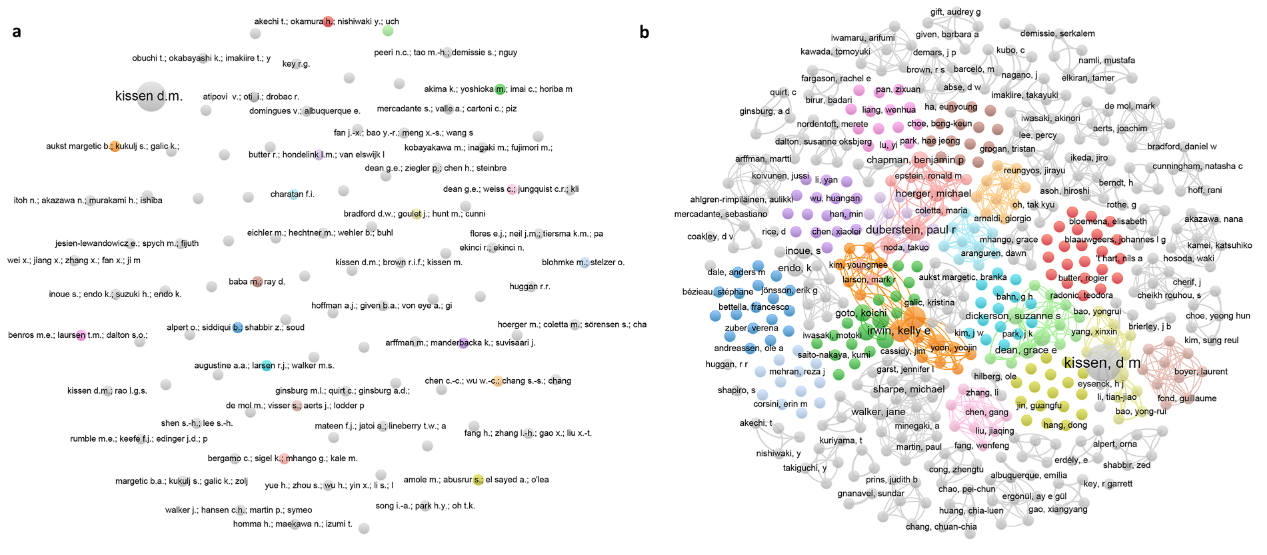
**

**Figure S8-G.** Co-citation network of the cited references (Scopus). Each node represents a reference that was cited at least 2 times. The size of circles and fonts demonstrates the number of citations of a reference. The thickness of the connecting line between references shows the frequency of co-citation.

Note: Scopus data on cited references may not have been harmonized. Reference strings may not have a consistent format.

**
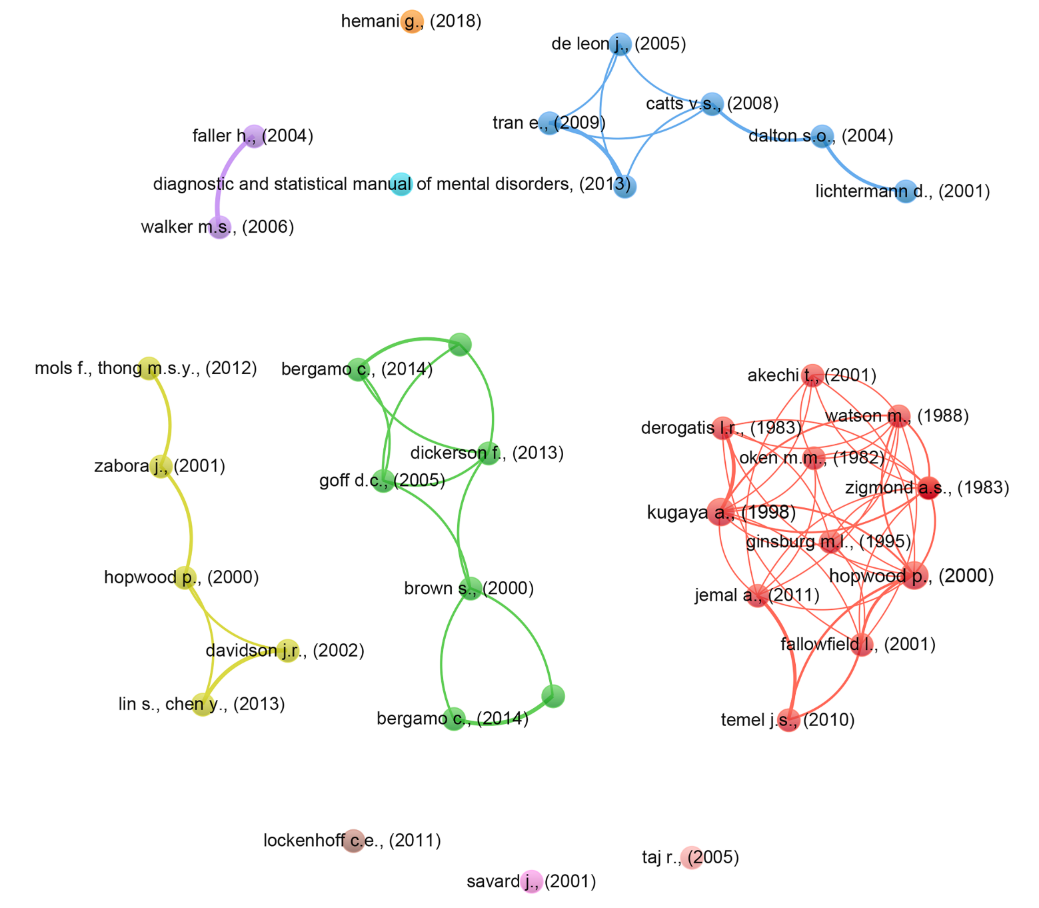
**
