# Supplementary material for: Causal effects between personality and psychiatric traits and lung cancer: a bidirectional two-sample Mendelian randomization and bibliometric study
Source: Front Psychiatry. 2024 Sep 12;15:1338481. doi: 10.3389/fpsyt.2024.1338481 (PMC11424467; doi:10.3389/fpsyt.2024.1338481)
Supplement: Supplementary file 2 [file Table2.docx]

(i) Search terms for Web of Science Core Collection (WoSCC)

#1: lung

#2: cancer or tumor or neoplasm or carcinoma

#3: personality or neuroticism or extraversion or agreeableness or conscientiousness or openness or psychi* or psychosis or psychoses or psychotic* or mental illness or mental disorder or mental patient* or schizo* or attention deficit or hyperactivity disorder or major depress* or autis* spectrum disorder or bipolar disorder or insomnia or sleepless*

#4: #1 AND #2 AND #3

Search field = Title

Indexes = Science Citation Index Expanded and the Social Sciences Citation Index.

Timespan = From January 1900 to May 2023.

Note: 153 results were retrieved, and 84 articles and 2 reviews were further filtered out for bibliometric analysis.

(ii) Search terms for Scopus

(TITLE(lung) AND TITLE(cancer or tumor or neoplasm or carcinoma) AND TITLE(personality or neuroticism or extraversion or agreeableness or conscientiousness or openness or psychi* or psychosis or psychoses or psychotic* or “mental illness" or "mental disorder" or "mental patient*" or schizo* or "attention deficit" or "hyperactivity disorder" or "major depress*" or "autis* spectrum disorder" or "bipolar disorder" or insomnia or sleepless*)) AND PUBYEAR < 2024

Timespan = From before 1960 to 2023.

Note: 116 results were retrieved and reviewed, and 101 articles and 1 review were further filtered out for supplementary bibliometric analysis. The completeness and reliability of the data in this database were inferior to those of the WoSCC.

(iii) Search terms for PubMed

((lung[Title]) AND (cancer[Title] OR tumor[Title] OR neoplasm[Title] OR carcinoma[Title])) AND (personality[Title] OR neuroticism[Title] OR extraversion[Title] OR agreeableness[Title] OR conscientiousness[Title] OR openness[Title] OR psychi*[Title] OR psychosis[Title] OR psychoses[Title] OR psychotic*[Title] OR mental illness[Title] OR mental disorder[Title] OR mental patient*[Title] OR schizo*[Title] OR attention deficit[Title] OR hyperactivity disorder[Title] OR major depress*[Title] OR autis* spectrum disorder[Title] OR bipolar disorder[Title] OR insomnia[Title] OR sleepless*[Title])

Timespan = From January 1900 to May 2023.

Note: 95 results were retrieved and reviewed, and 92 articles and 3 reviews were used for supplementary bibliometric analysis. The database contained no citation data. The completeness and reliability of bibliometric data were relatively poor.
