# Supplementary material for: Causal effects between personality and psychiatric traits and lung cancer: a bidirectional two-sample Mendelian randomization and bibliometric study
Source: Front Psychiatry. 2024 Sep 12;15:1338481. doi: 10.3389/fpsyt.2024.1338481 (PMC11424467; doi:10.3389/fpsyt.2024.1338481)
Supplement: Supplementary file 4 [file Table4.docx]

**STROBE-MR checklist of recommended items to address in reports of Mendelian randomization studies**^1^ ^2^

| **Item No.** | **Section** | **Checklist item** | **Page No.** | **Relevant text from manuscript** |
| --- | --- | --- | --- | --- |
| 1 | **TITLE and ABSTRACT** | Indicate Mendelian randomization (MR) as the study’s design in the title and/or the abstract if that is a main purpose of the study | 1 | Causal effects between personality and psychiatric traits and lung cancer: A bidirectional two-sample Mendelian randomization and bibliometric study |
|  | **INTRODUCTION** |  |  |  |
| 2 | **Background** | Explain the scientific background and rationale for the reported study. What is the exposure? Is a potential causal relationship between exposure and outcome plausible? Justify why MR is a helpful method to address the study question | 2-3 | Personality and psychiatric traits are involved in carcinogenesis. LC risk is associated with neuroticism and extraversion.  Patients with schizophrenia may have a higher LC risk than the general population. A previous meta-analysis supported the protective effect of schizophrenia on LC. However, another meta-analysis concluded that the association lacked certainty.  Mendelian randomization (MR) is an epidemiological methodology that uses genetic variants, including single-nucleotide polymorphisms (SNPs), as instrumental variables (IVs) to estimate the causality between exposure and outcome. MR analysis constitutes a natural randomized controlled trial that assesses causality between exposure and outcome at the genetic level while excluding reverse causality. |
| 3 | **Objectives** | State specific objectives clearly, including pre-specified causal hypotheses (if any). State that MR is a method that, under specific assumptions, intends to estimate causal effects | 3 | Notably, previous MR studies have investigated the causal effects of neuroticism and insomnia on LC and found that both are positively associated with an increased LC risk. However, previous findings reported in rigorously designed prospective cohort studies did not draw that conclusion. Thus, we conducted this study which aimed to elucidate whether there is a forward or reverse causal association between personality and psychological traits and LC and its subtypes and to further elucidate the nature of this potential association. We also present an overview of the research dynamics and landscape using science mapping.  The causal inference of MR relies on the following three core assumptions: relevance, independence, and exclusion restriction (Figure S1). |
|  | **METHODS** |  |  |  |
| 4 | **Study design and data sources** | Present key elements of the study design early in the article. Consider including a table listing sources of data for all phases of the study. For each data source contributing to the analysis, describe the following: |  |  |
|  | a) | Setting: Describe the study design and the underlying population, if possible. Describe the setting, locations, and relevant dates, including periods of recruitment, exposure, follow-up, and data collection, when available. | 3-4 | The overall study design is presented in Figure 1.  SNPs for neuroticism were obtained from the UK Biobank and the MRC Integrative Epidemiology Unit (MRC-IEU) …... The summarized statistics analyzed in this study were approved by the ethics committees of the original studies. |
|  | b) | Participants: Give the eligibility criteria, and the sources and methods of selection of participants. Report the sample size, and whether any power or sample size calculations were carried out prior to the main analysis | 3-4 | SNPs for neuroticism were obtained from the UK Biobank and the MRC Integrative Epidemiology Unit (MRC-IEU), which included 374,323 individuals of European ancestry …... The participants were patients with LC with European ancestry (n = 85,716; 29,266 cases and 56,450 controls).  The participants in this GWAS were LC patients with European ancestry (n = 85,716; 29,266 cases and 56,450 controls; the demographic characteristics are listed in Additional file 2: Table S1) …… Additionally, the IVs for insomnia were acquired from the MRC-IEU database, comprising data from 462,341 individuals of European ancestry. |
|  | c) | Describe measurement, quality control and selection of genetic variants | 4 | Typically, we selected instrumental SNPs using a *P*-value < 5 × 10^-8^ and linkage disequilibrium (LD) r^2^ < 0.001 within a 10,000-kb window. …… Therefore, we utilized SNPs with a more relaxed threshold, and this set the minimum extraction criteria to P < 5 × 10-6 and LD r2 < 0.01 within a 5,000-kb window. |
|  | d) | For each exposure, outcome, and other relevant variables, describe methods of assessment and diagnostic criteria for diseases | 4 | The diagnosis of LC requires pathological or cytological confirmation.  Detailed information, such as recruitment process and genetic data quality control, can be found in the original studies. |
|  | e) | Provide details of ethics committee approval and participant informed consent, if relevant | 4 | The use of the summarized statistics analyzed in this study was approved by the ethics review committees of the original studies. |
| 5 | **Assumptions** | Explicitly state the three core IV assumptions for the main analysis (relevance, independence and exclusion restriction) as well assumptions for any additional or sensitivity analysis | 3 | The causal inference of MR relies on the following three core assumptions: relevance, independence, and exclusion restriction (Figure S1) |
| 6 | **Statistical methods: main analysis** | Describe statistical methods and statistics used |  |  |
|  | a) | Describe how quantitative variables were handled in the analyses (i.e., scale, units, model) | NA |  |
|  | b) | Describe how genetic variants were handled in the analyses and, if applicable, how their weights were selected | 4 | We calculated the F-statistics for each genetic instrument to assess the strength of the genetic variants, considering an F-statistic > 10 indicative of a strong IV ……We investigated whether these SNPs were linked to LC risk factors, including tobacco smoking， environmental tobacco smoke exposure， obesity, and alcohol consumption, and excluded SNPs that exhibited genome-wide associations with these confounding factors. |
|  | c) | Describe the MR estimator (e.g. two-stage least squares, Wald ratio) and related statistics. Detail the included covariates and, in case of two-sample MR, whether the same covariate set was used for adjustment in the two samples | 4 | We used random-effects inverse variance weighting (IVW), MR-Egger, and the weighted median as MR models.  Detailed information, such as recruitment process and genetic data quality control, can be found in the original studies. |
|  | d) | Explain how missing data were addressed | 4 | Detailed information, such as recruitment process and genetic data quality control, can be found in the original studies. |
|  | e) | If applicable, indicate how multiple testing was addressed | 5 | A Bonferroni-corrected P-value < 0.05/48 (48 is the product of the number of exposures and the number of outcome events) = 0.001 was considered statistically significant for multiple comparisons. A P-value threshold of 0.001 was deemed reliable. |
| 7 | **Assessment of assumptions** | Describe any methods or prior knowledge used to assess the assumptions or justify their validity | 5 | Horizontal pleiotropy was assessed using the MR-Egger intercept test, with P-values < 0.05 suggesting a high level of pleiotropic bias that could influence causal estimation. |
| 8 | **Sensitivity analyses and additional analyses** | Describe any sensitivity analyses or additional analyses performed (e.g. comparison of effect estimates from different approaches, independent replication, bias analytic techniques, validation of instruments, simulations) | 5 | If the estimates were inconsistent across different MR models, the P-value threshold would need to be tightened, followed by re-running the MR analysis.  Cochran's Q test was used to estimate heterogeneity, with P < 0.05 indicating significance ……Moreover, funnel plots were used to evaluate probable directional pleiotropy. |
| 9 | **Software and pre-registration** |  |  |  |
|  | a) | Name statistical software and package(s), including version and settings used | 5 | Statistical analyses for the MR analysis were performed using the TwoSampleMR (version 0.5.6) package in R (version 4.2.3). |
|  | b) | State whether the study protocol and details were pre-registered (as well as when and where) | NA |  |
|  | **RESULTS** |  |  |  |
| 10 | **Descriptive data** |  |  |  |
|  | a) | Report the numbers of individuals at each stage of included studies and reasons for exclusion. Consider use of a flow diagram | 4 | Detailed information, such as recruitment process and genetic data quality control, can be found in the original studies.  Table S1. |
|  | b) | Report summary statistics for phenotypic exposure(s), outcome(s), and other relevant variables (e.g. means, SDs, proportions) | 6-7 | To genetically predict neuroticism, we used a total of 116 SNPs. Additionally, we used four, two, five, and eight SNPs for extraversion, agreeableness, conscientiousness, and openness, respectively (Tables S2–S6). Overall, 217 SNPs were used to predict schizophrenia, including 12, 18, 10, 16, 42, and 13 for ADHD, MDD, ASD, BD, insomnia, and anxiety, respectively (Tables S7–S13).  Fifteen SNPs were used to genetically predict overall LC, whereas 7, 13, and 2 SNPs were used to predict LUSC, LUAD, and SCLC, respectively (Tables S27–S30). |
|  | c) | If the data sources include meta-analyses of previous studies, provide the assessments of heterogeneity across these studies | 4 | Detailed information, such as recruitment process and genetic data quality control, can be found in the original studies.  Table S1. |
|  | d) | For two-sample MR:  i.  Provide justification of the similarity of the genetic variant-exposure associations between the exposure and outcome samples  ii.  Provide information on the number of individuals who overlap between the exposure and outcome studies | 3-4 | SNPs for neuroticism were obtained from the UK Biobank and the MRC Integrative Epidemiology Unit (MRC-IEU), which included 374,323 individuals of European ancestry …… Detailed information, such as recruitment process and genetic data quality control, can be found in the original studies.  Table S1. |
| 11 | **Main results** |  |  |  |
|  | a) | Report the associations between genetic variant and exposure, and between genetic variant and outcome, preferably on an interpretable scale |  | Tables S2-S25, Tables S27-S34. |
|  | b) | Report MR estimates of the relationship between exposure and outcome, and the measures of uncertainty from the MR analysis, on an interpretable scale, such as odds ratio or relative risk per SD difference | 6-7 | The analysis revealed a significant association between genetically proxied schizophrenia and increased risk of overall LC (odds ratio [OR] = 1.077, 95% CI = 1.030–1.126, P = 0.001).  The analysis revealed a significant causal relationship between genetically predicted overall LC and increased ADHD risk (OR = 1.221, 95% CI = 1.096–1.362, P < 0.001).  Figure 2, Figure 3, Table S26, Table S35. |
|  | c) | If relevant, consider translating estimates of relative risk into absolute risk for a meaningful time period | NA |  |
|  | d) | Consider plots to visualize results (e.g. forest plot, scatterplot of associations between genetic variants and outcome versus between genetic variants and exposure) |  | Figure 2, Figure 3, Figure S2-S7. |
| 12 | **Assessment of assumptions** |  |  |  |
|  | a) | Report the assessment of the validity of the assumptions | 6-7 | Furthermore, the MR-Egger intercept test did not identify any pleiotropy (P > 0.05), indicating the absence of pleiotropic bias in certain contexts of heterogeneity.  The MR-Egger intercept was < 0.05 only when examining the impact of overall LC on ASD; this suggests the potential presence of pleiotropy. The remaining MR-Egger intercept calculations yielded P-values > 0.05, indicating no significant horizontal pleiotropy. |
|  | b) | Report any additional statistics (e.g., assessments of heterogeneity across genetic variants, such as *I^2^*, Q statistic or E-value) | 6-7 | Cochran's Q test revealed heterogeneity in the causal inference between schizophrenia and LC risk (P < 0.05); however, this heterogeneity was acceptable because we used the IVW random effect model to obtain the main result.  Cochran's Q test detected no significant heterogeneity in the causal inference between overall LC and ADHD risk (P > 0.05). |
| 13 | **Sensitivity analyses and additional analyses** |  |  |  |
|  | a) | Report any sensitivity analyses to assess the robustness of the main results to violations of the assumptions | 6-7 | Cochran's Q test revealed heterogeneity in the causal inference between schizophrenia and LC risk (P < 0.05); however, this heterogeneity was acceptable because we used the IVW random effect model to obtain the main result ……The estimates revealed that the significant causal effect detected was not biased by specific SNPs.  Cochran's Q test detected no significant heterogeneity in the causal inference between overall LC and ADHD risk (P > 0.05) ……The significant causal effect did not rely on a lone SNP. |
|  | b) | Report results from other sensitivity analyses or additional analyses | 6-7 | The F-statistics of the instrumental SNPs was > 10, indicating that the IVs had sufficient predictive strength for these traits.  Similar to forward MR analyses, the F-statistics of the SNPs exceeded the critical value of 10. |
|  | c) | Report any assessment of direction of causal relationship (e.g., bidirectional MR) | 6-7 | Causal effects of personality and psychiatric traits on LC  Causal effects of LC on personality and psychiatric traits |
|  | d) | When relevant, report and compare with estimates from non-MR analyses | 7-8 | Bibliometric analysis |
|  | e) | Consider additional plots to visualize results (e.g., leave-one-out analyses) |  | Figure S2-S7. |
|  | **DISCUSSION** |  |  |  |
| 14 | **Key results** | Summarize key results with reference to study objectives | 8 | The results demonstrated a causal effect of genetically predicted schizophrenia on overall LC risk, along with a causal effect of genetically predicted overall LC on ADHD. |
| 15 | **Limitations** | Discuss limitations of the study, taking into account the validity of the IV assumptions, other sources of potential bias, and imprecision. Discuss both direction and magnitude of any potential bias and any efforts to address them | 10 | This study has some limitations. First …… |
| 16 | **Interpretation** |  |  |  |
|  | a) | Meaning: Give a cautious overall interpretation of results in the context of their limitations and in comparison with other studies | 8-9 | To the best of our knowledge ……into the biopsychosocial models. |
|  | b) | Mechanism: Discuss underlying biological mechanisms that could drive a potential causal relationship between the investigated exposure and the outcome, and whether the gene-environment equivalence assumption is reasonable. Use causal language carefully, clarifying that IV estimates may provide causal effects only under certain assumptions | 9 | Protective and behavioral confounding factors in patients with schizophrenia may mask the associated LC risk. Indeed, schizophrenia may affect LC by altering neuroendocrine and immune functions. Cohort studies have demonstrated that patients with schizophrenia have a higher incidence rate of LC. No randomized controlled clinical trials have provided conclusive evidence thus far. However, considering the perspective of biopsychosocial models, schizophrenia may influence physiological health through specific mechanisms, potentially leading to the development of lung cancer. |
|  | c) | Clinical relevance: Discuss whether the results have clinical or public policy relevance, and to what extent they inform effect sizes of possible interventions | 9 | Based on our positive results, we propose that LC screening may be necessary for patients with schizophrenia.  Additionally, healthcare providers should remain vigilant for the potential occurrence of LC in patients with ADHD. |
| 17 | **Generalizability** | Discuss the generalizability of the study results (a) to other populations, (b) across other exposure periods/timings, and (c) across other levels of exposure | 10 | The included populations were overwhelmingly European; therefore, these findings may not apply to other races. |
|  | **OTHER INFORMATION** |  |  |  |
| 18 | **Funding** | Describe sources of funding and the role of funders in the present study and, if applicable, sources of funding for the databases and original study or studies on which the present study is based | 11 | Funding  None. |
| 19 | **Data and data sharing** | Provide the data used to perform all analyses or report where and how the data can be accessed, and reference these sources in the article. Provide the statistical code needed to reproduce the results in the article, or report whether the code is publicly accessible and if so, where | 16 | Data Availability Statement  Tables S2-S25, Tables S27-S34. |
| 20 | **Conflicts of Interest** | All authors should declare all potential conflicts of interest | 10 | The authors declare that the research was conducted in the absence of any commercial or financial relationships that could be construed as a potential conflict of interest. |

This checklist is copyrighted by the Equator Network under the Creative Commons Attribution 3.0 Unported (CC BY 3.0) license.

1. Skrivankova VW, Richmond RC, Woolf BAR, Davies NM, Swanson SA, VanderWeele TJ, et al. Strengthening the Reporting of Observational Studies in Epidemiology using Mendelian Randomisation (STROBE-MR): Explanation and Elaboration. BMJ. 2021;375:n2233.
